# FinTech Adoption and ESG Disclosure in Corporate Valuation: Intellectual Capital and Financial Performance Effects on Dividend Policy and Firm Value

**DOI:** 10.12688/f1000research.178277.2

**Published:** 2026-06-23

**Authors:** Md Qamruzzaman, Abdulrahman Alomair, Mohammed Alomair

**Affiliations:** 1 School of Business and Economics, United international University, Dhaka, 1212, Bangladesh; 2 Accounting Department, , Business School, King Faisal University, Al Ahsa, 31982, Saudi Arabia

**Keywords:** FinTech Adoption, ESG Disclosure, Intellectual Capital, Dividend Policy, Corporate Valuation

## Abstract

**Background:**

This paper examines the complex interconnections among FinTech adoption, intellectual capital, ECG disclosure, and dividend policy, and their impacts on corporate value in financial institutions of emerging economies. Questioning traditional corporate finance theory, which often undervalues non-physical assets, the scholarship is propelled by the growing gap between market and book value, which, in turn, is enhanced by the process of digital transformation and the strategic importance of data and intellectual property.

**Method:**

Our hypothesis is formulated as an opportunity to explain and confirm the full effect of these variables on a company’s value through a complex framework that will successfully fill a gap in the existing literature on the synergistic synthesis of these variables in the so-called Digital-ESG-Value nexus. Using a sample of DSE-listed financial institutions from 2015 to 2023, we employ rigorous econometric methods, including fixed-effects models, dynamic panel GMM, CS-ARDL, quantile regressions, and deep neural network models, to ensure our conclusions are sound and valid. Findings The empirical findings clearly show that FinTech adoption, intellectual capital, ESG disclosure, and dividend policy, when combined with various proxies, have a significant positive influence on firm value. ESG disclosure is observed to have a substantial moderating effect on the dividend policy and firm value, strengthening the plausibility of dividend payments. Financial performance also serves as a moderating factor between ESG and the dividend policy. Quantile regressions also help understand heterogeneity, showing that better-performing companies reap disproportionately from these strategic features.

**Findings:**

The empirical findings clearly show that FinTech adoption, intellectual capital, ESG disclosure, and dividend policy, when combined with various proxies, have a significant positive influence on firm value. ESG disclosure is observed to have a substantial moderating effect on the dividend policy and firm value, strengthening the plausibility of dividend payments. Financial performance also serves as a moderating factor between ESG and the dividend policy. Quantile regressions also help understand heterogeneity, showing that better-performing companies reap disproportionately from these strategic features.

**Conclusion:**

These results have significant theoretical implications by applying the assumptions of Signaling Theory, the Resource-Based View, and Stakeholder Theory in an evolving digital environment. In practice, the research provides practical guidance to managers who need to determine how to allocate resources to maximise the sustainability of value creation between technology and human resource.

## I. Introduction

This paradigm shift requires a re-evaluation of traditional theories of corporate finance, which have conventionally predicted that physical capital is a more accurate predictor of the intrinsic determinants of firm valuation in present-day markets. It can be seen in the platform economy, where Uber is one such business whose value is almost entirely derived from customer and driver networks rather than physical resources ( Beaumont, 2019). The increasing visibility of digital transformation, artificial intelligence, and big data has only enhanced the strategic importance of intangible assets such as data, brand equity, and intellectual property as the tangibility of assets globally declines ( Vengesai, 2023). The example of this dynamic is the increasing difference between the markets and the book valuation, which is caused by the growing significance of intangible capital investments in driving corporate innovation and long-term sustainability; to be more precise, the book equity has recorded systematic decrease in the U.S. firms as the importance of intangible capital investments in the form of knowledge and human capital investment increases ( Luo, 2020). Those phenomena challenge established valuation models, i.e., the ones propounded by Fama and French, which are largely based on physical book values and therefore factor into the recent underperformance of the value factor, often causing the misrepresentation of the actual value of data-based companies like Facebook and Twitter and any other entities with large amounts of intangible capital ( Eisfeldt et al., 2020; Shi, 2024). As such, it is urgently required to understand the economic implications of these intangible assets, specifically, their impact on the returns of equity, the cost of capital, and the valuation of the firm overall as an investor ( Dong and Doukas, 2025).

The use of FinTech through the focus on intangible assets in the spectrum of data and artificial intelligence ( Vengesai, 2023), and the introduction of emerging technologies, including AI, blockchain, and big-data analytics has had a significant effect on reconfiguring the traditional financial models and, in turn, spawned innovation and efficient work of the capital market ( Lăzăroiu et al., 2023). This integrative technological paradigm enhances the effectiveness, transparency, and cost-efficiency of financial transactions through blockchain-based data provisioning and AI-intuited analytics, thereby transforming the competitive landscape of both established financial institutions and new market entrants ( Beaumont, 2019; Hasan et al., 2024). The implication of FinTech goes beyond operational improvements, whereby corporate investment direction and ultimately the value of the firm have been impacted through more secure, efficient, and cost-reduced financial transactions. In addition, strong sustainability reporting in FinTech companies correlates with high valuations and better market performance ( Yuan et al., 2024). This transformational ability refers to shifting the paradigm from products to clients and requires a flexible, technology-driven solution to remain relevant in the market ( Ahangar and Salman, 2020). The implications of its extensive implementation of artificial intelligence in FinTech activities, specifically, are that the value generation changes its form: no longer to traditional revenue-generating models, but to investor confidence instead, and the importance of AI as a transformative force ( Visconti, 2024). The issues posed by these new technological paradigms necessitate reevaluating existing financial and economic paradigms through the prism of sustainability and machine learning, thereby allowing the formulation of new frameworks of analysis ( Berrou, 2021).

This focus on intellectual capital 3 both human expertise, organisational procedures and customer relations points to an even larger shift towards recognising these aspects as key sources of competitive edge, as well as drivers of economic growth in the knowledge economy. Corporate innovation and long-term survival are increasingly dependent on intangible investments in human capital and knowledge ( Luo, 2020). Companies with high levels of intangible assets are more likely to focus on the quality of financial reporting to enhance market transparency, thereby allowing stakeholders to benefit from more accurate price changes ( Dong and Doukas, 2025). This kind of transparency is also supported by FinTech developments that advance the reporting of Environmental, Social, and Governance (ESG) matters and promote financial inclusion by democratising the influence of green investments ( Hasan et al., 2024). The development of FinTech, in particular the use of AI, the Internet of Things, big data analytics, and blockchain, plays a crucial role in advancing green and sustainable financial services and products, as evidenced by the development of consistent climate-risk assessment systems. Simultaneously, strong sustainability metrics in FinTech firms are associated with high valuations and better market results ( Lăzăroiu et al., 2023; Merello et al., 2021). Its nature is driven by the convergence of sustainable finance values and ESG concerns, and it is key to supporting the growth trend of inclusive growth and stability in global financial markets during times of volatility, as evidenced by the ability to reduce risks and enhance the stability of interconnected financial markets ( Arnone and Leogrande, 2024; Mani, 2024).

This urgency highlights why corporations must not only establish financial performance but also strive to impact the environment and society positively, including the aspect of ethical governance, and openly convey these efforts to the stakeholders, which makes them more resilient and appealing to investors ( Arfaoui-Masmoudi and Hazami-Ammar, 2024; Merello et al., 2021). Continuing the ESG transparency and financial inclusion innovations in the FinTech industry ( Hasan et al., 2024), the ubiquitous integration of ESG factors into the investment process represents a vital paradigm shift, making these aspects inseparable from full-fledged investment strategies and decision-making ( Shah, 2024). This evolution highlights the substantial role of enhanced analytics, i.e. AI, big data, and blockchain, in the evaluation and incorporation of ESG performance ( Lăzăroiu et al., 2023; Olanrewaju et al., 2024). Global regulators are subjecting companies to wider and more comprehensive disclosure requirements on sustainability/environmental, social, and labour (ESL) practices; to give one example, the European Union Corporate Sustainability Reporting Directive focuses on ensuring that large and small companies engage in comprehensive reporting on their sustainability practices (environmental, social, and labour) ( Shirai, 2023). This is the regulatory wave, which, along with an increasing interest in sustainable investments among investors, strengthens the inextricable connection between strong ESG performance and long-term financial sustainability, including high valuation, strong market performance, and elevated shareholder returns ( Ahangar and Salman, 2020; Arnone and Leogrande, 2024; Merello et al., 2021).

This difficulty lies in the very nature of the intellectual capital, which is intangible and subjective in nature - an area where there is no consensus on its definition, measurement and an appropriate framework - and is made difficult by the fact that even valuation processes of AI-driven technologies in the financial sector are nascent and non-standardised ( Ammar and Kamaruddin, 2025). The lack of uniform valuation and accounting frameworks for technology and information assets in FinTech adds to this challenge, preventing their consistent placement on balance sheets and undermining the overall reliability of the market ( Bayón and Vega, 2018). Further, artificial intelligence has a significant transformative impact on the value-creation process, altering operational patterns, revenues, and investor trust in organisations and enterprises operating in the FinTech sphere. However, its exact contribution to firm value is still elusive, as it can either be a factor that improves the other intangibles or the result of investments in intellectual capital, either way depending on the presence or absence of various institutional settings such as market efficiency and regulatory preparedness ( Elkmash and Mohamed, 2025; Visconti, 2024). Indeed, in a recent example, innovation has a role of great complexity and subsidence in capital structure; a decline in the intensity of R&D could be an indication of competitive disadvantages or growth constraints, which would subsequently drive negative adjustments in investor valuation ( Kruglov and Shaw, 2024). On the contrary, although intellectual capital has the potential to add value, it is not always a good predictor of firm value due to model constraints that fail to account for mediating variables, such as profitability, and to capture the financial impact of investments on intellectual capital ( Appah et al., 2023). This disparity also extends to the performance reporting of intellectual capital, with most companies not clearly disclosing its impact on firm value in annual or sustainability reports, making it difficult for investors to assess its impact amid an unresolved measurement dilemma ( Kruglov and Shaw, 2024). Also, this effect of evidence is particularly strong in less developed markets, where institutional and structural peculiarities, such as insufficient integration of ESG and methodological flaws (e.g., endogeneity), further moderate these dynamics ( AlQudah et al., 2025). Also, little has been done in the area of optimal balance and synergistic integration of diverse knowledge assets with corporate social responsibility to optimise market value ( Christofi et al., 2024). Finally, the complex interdependence of these intangibles, including strategy, ownership, management, CSR, and external factors, and how they are transformed into sustainable competitive advantage and shareholder returns, is an issue that will require stringent empirical investigation to guide and improve valuation models ( Campo and Calvo, 2025).

The financial world is undergoing a broad paradigm shift that casts aside the old assumptions of corporate finance, which historically favoured physical capital in firm valuation. The rise of digital transformation, artificial intelligence, and big data has elevated intangible assets, including data reserves, brand value, and intellectual property, to a higher level of strategic significance, increasing the disparity between market and book values. A notable example of a company in which customer networks generate more value than material resources underscores the urgency of revaluing valuation models, which often underestimate the real value of data-centric, or intangible-intensive, organisations. FinTech, with its emphasis on data analytics and AI, has simplified traditional financial approaches, enabling innovation and efficiency across capital markets. Such technological development cannot be thought of without the contemporary pressure on corporations to deliver fiscal results while, at the same time, providing favourable environmental and social outcomes, anchored in ethical control. However, even though there is unanimous agreement on the applicability of intellectual capital and ESG, a definitive consensus on definitions and measurement remains a distant reality, especially regarding the exact effect of AI-based technologies on valuation. As a result, the gap in understanding how to utilise synergies between knowledge assets and corporate social responsibility to maximise market value is significant. Thus, this research aims to shed light on the combined effects of FinTech adoption, intellectual capital, and ESG disclosure on corporate valuation and dividend policy, thereby filling a gap in the literature by exploring these complex relationships among financial institutions. The guidelines that guide the research are as follows:

RQ1: Does the FinTech Adoption and Intellectual Capital have a significant positive impact on the quality of ESG Disclosures?

RQ2: Does increased ESG Disclosure have any effect on the Dividend Policy of the firm?

RQ3: Does the Financial Performance of firms mediate the relationship between ESG practices and the Dividend Policy of a firm?

RQ4: How much does Dividend Policy serve as an intervention towards improving overall Firm Value?

This exploration makes a significant contribution to both academic intellectual writing and practitioner decision-making by exhaustively analysing the complex dynamics of FinTech adoption, intellectual capital, ESG disclosure, and dividend policy in the valuation of companies in the emerging financial industry. First, it widens the Digital-ESG-Value nexus by empirically testing a comprehensive conceptual framework that integrates FinTech adoption and intellectual capital as antecedents of ESG disclosure, thereby informing dividend policy and, ultimately, firm value. It is evident that although these constructs are usually analysed separately, they are interrelated in a synergistic manner, as shown in this work. Specifically, the research builds on Signalling Theory by demonstrating that ESG disclosures amplified by FinTech, as well as dividend policies, are strong indicators of a company’s financial soundness and its commitment to sustainable development and, as such, help alleviate information asymmetry, generating trust among investors. It also adds dimension to the Resource-Based View by pre-viewing intellectual capital as human expertise, procedural sophistication, and customer relationships as distinct, non-replicable sources of competitive edge and value creation where digital abilities and ESG strategy intersect. Also, the study aligns with Stakeholder Theory and demonstrates that a robust ESG practice, enabled by FinTech, effectively meets the expectations of various stakeholders for transparency and accountability, thereby driving reputation-based capital creation that enhances long-term firm value. Through the application of modern econometric tools, the research finds the direct connection as well as the crucial mediating role of financial performance to the ESG-dividend policy nexus, explains the mediating processes of the ESG disclosure and dividend policy on the value-creation process, and provides a more subtle insight into the critical interdependences between these multifaceted relationships.

On the managerial front, such research provides prescriptive guidance on how managers can best allocate resources between technological adoption and human capital development. The results confirm that the use of FinTech and intellectual capital is a powerful driver of firm value, supporting strategic decisions to invest in two concepts: digital transformation and intellectual resources. Managers can use FinTech to improve the efficiency, transparency, and accessibility of ESG reporting, which has been proven to increase market value and investor attitude. It is important to understand the moderating impact of financial performance: financially sound companies are more likely to bear the costs of extensive ESG reporting and maintain stable dividend distributions, thereby resolving the perceived conflict between sustainability obligations and shareholder income. This study, therefore, provides managers with a template from which they can develop integrated strategies that will help them to utilise FinTech competencies and intellectual capital in not only increasing their operating efficiencies and regulatory compliance, but also using their credible ESG reporting and consistent policies of paying dividends as an indicator of strategic financial stability and sustainability, thus maximising firm value.

To regulators and policymakers, the results are persuasive in terms of endorsing policies that promote the use of FinTech and compel disclosure of ESGs. The fact that the implementation of FinTech is positively associated with firm value and enhances the transparency of the e-sustainability system shows that the regulatory environment supporting digital transformation in the financial field can build stronger, more effective markets. The average positive correlation between ESG reporting and company valuation, observed across various econometric specifications, supports increasing the level of mandatory ESG reporting in line with the European Union mandate. These laws would reduce information asymmetry, restore investor confidence, and enable capital to be allocated to sustainable businesses. In addition, expanding on the employability of intellectual capital as a strategic priority, the study proposes using policy tools to track and reward investment in intangible resources within financial institutions, ensuring that regulatory frameworks are receptive to the emerging modes of value creation in the digital economy.

Remaining structure of the article are as follows. Section II deals with theoretical development and literature review, data and methodology explained in Section III, result and discussion available in Section IV, practical implications and theoretical implications reported in Section V and VI, finally conclusion available in Section VII, respectively.

## II. Theoretical underpinning and literature review

### 2.1 Theoretical foundations

*Agency Theory:*

Free cash flows are very high, which could encourage managers to waste. Agency costs are minimised by paying dividends or repurchasing common stock, thereby preventing funds from being spent on unprofitable ventures. Disciplined payout policy, in its turn, is expected to increase the firm value. The school of thought holds that dividends can reduce moral hazard by forcing managers to finance their activities externally, thereby exposing the decision-making process to market scrutiny. Additionally, dividends may signal a firm’s financial situation and outlook, particularly when information asymmetry exists, thereby affecting investor preferences and share prices ( Barclay and Smith, 1988). This is because regular dividend payments signal that a firm is confident it will remain profitable and liquid over the long term, which may reassure investors and lead to an understated cost of capital ( Arnone and Leogrande, 2024; Bayón and Vega, 2018). On the other hand, the bird-in-hand theory implies that investors will give more weight to present dividends than to perceived future capital gains, which makes firms with regular dividends pay out to have higher firm valuations ( Demirgüneş, 2015). Such a choice is explained by reduced uncertainty about future cash flows, which makes dividend-paying stocks more appealing to risk-averse investors.

*Signalling Theory:*

The corporate policies act as signals. For example, higher dividends or voluntary ESG reporting can signal good things to come and management-led governance. Successful companies are prone to pay dividends that are way below the board to stand out. Such behaviour is consistent with the principle that alterations in dividend payments may have important implications for investors, especially for the financial health of a firm and its prospects, thereby affecting its market value ( Fanning and Brittain, 1967). This applies especially in the case of asymmetric information, i.e., when managers have better knowledge of the firm’s actual performance and prospects than external investors ( El-Deeb and Allam, 2024; Nazir and Nawaz, 2012). Thus, those with high prospects resort to dividend payments as a plausible cue to communicate their strong financial condition and future growth to the market ( Al-Najjar and Kilincarslan, 2019). On the other hand, a low or absent dividend may also be a bad indicator, reflecting financial volatility or pessimism, and this will result in falling share prices and investor confidence ( Yurko, 2018). What is more, regular dividends would help reduce asymmetric information between management and investors, as they would show that a company can generate sufficient cash flows to maintain such payments ( Charbti, 2020; Damayanti and Dinaseviani, 2024). The signalling effects of dividends are more important for mature companies, which, due to high accumulated profits and other sources of self-funding, are more likely to pay higher dividends to shareholders ( AlQudah et al., 2025).

*Resource-Based View (RBV):*

Knowledge, innovation, and digital capabilities can be characterised as intangible assets that are scarce and confer a competitive advantage. Intellectual capital is the sum of human capital, structural capital, and relational capital, one of the driving forces of performance. Companies that invest in FinTech also develop unspoken capabilities (such as data infrastructure and analytics) that can increase productivity and value ( Chen and Srinivasan, 2023; Lăzăroiu et al., 2023). This perspective argues that intellectual capital, including human, structural, and relational assets, is a valuable, hard-to-duplicate asset that underpins a firm’s long-run competitive advantage and value creation ( Kadim et al., 2020). This school of thought also assumes that companies with stronger internal resources and capabilities, including those that can be developed through FinTech investments, are better positioned to achieve higher profitability and market valuation by leveraging these specific assets to innovate and differentiate in the market. This is given that even when such resources are efficiently deployed, firms can build unique competencies, increase operational efficiency, improve product or service offerings, and eventually achieve better financial performance ( Essel, 2025).

*Stakeholder Theory:*

The existence of firms is to serve their stakeholders (customers, employees, community, and regulators). ESG disclosure is driven by stakeholders’ requirements for transparency and accountability. ESG practices build reputational capital and legitimacy, thereby promoting long-term value by taking stakeholders’ concerns into account. This method combines the principles of agency theory and stakeholder theory, implying that organisations align their environmental, social, and governance investments with different stakeholder interests to increase profitability ( Wang et al., 2025). Moreover, the long-term performance and sustainability of a firm are inevitably tied to stakeholder approval and support; the firm’s management must balance and meet the expectations of the various parties. A combination of these theoretical lenses, specifically the resource-based perspective and stakeholder theory, provides a background on how sustainability and innovation serve as mutually reinforcing instruments for creating competitive advantage and increasing firm value ( Widagdo and Rahmawati, 2025). The concept of ESG practices’ power to enhance social capital through stakeholder interactions strengthens this synergistic relationship, as social capital is an important intangible asset that drives a firm’s competitive advantage and performance.

*Information Asymmetry:*

It is common for managers to be more knowledgeable than investors. ESG reports (voluntary disclosures) and digital financial statements minimise this gap. The use of FinTech applications (such as blockchain and big data) also enhances transparency and data accuracy, further alleviating information asymmetry. Minimising asymmetry can reduce the cost of capital and increase firm value. Such a decrease in information asymmetry, enabled by the development of highly sophisticated FinTech solutions, results in a more efficient allocation of capital and, possibly, an increase in firm valuation because investors are now more likely to trust the information disclosed ( Toumi et al., 2023). Such increased disclosure also fosters trust among investors who are essential in attracting capital and promoting eventual financial stability ( Najaf et al., 2024). In addition, high ESG results particularly reduce information asymmetry, which facilitates resource acquisition; thus, innovation becomes more available and viable due to reduced sunk costs and financing constraints arising from the high level of risk in research and development. Combined, these views represent how ESG disclosure simultaneously serves as a response to stakeholder pressures, a source of strategic benefit, a credibility-enhancing indicator, and a tool of governance ( Metwally et al., 2025).

*Technology Adoption (Diffusion):*

Classical diffusion paradigms (Rogers) and the technology acceptance paradigm assume that firms adopt innovations to achieve efficiency and competitiveness. Use of FinTech is a technology decision. Companies operating in FinTech should benefit from automated business operations, innovative financial services (e.g., mobile payments), and expanded market reach. This aligns with the literature on innovation and digital transformation ( Brehmer, 2023). It is especially topical in high-tech industries that can better afford FinTech due to their large resources and research-and-development focus, enabling sustainable growth and quick market demand adjustments. Such a combination of FinTech with high-technology fields not only contributes to operational efficiencies but also to the prospect of developing new financial products to meet existing and emerging investor needs, namely, socially responsible and technology-enhanced investment opportunities ( Najaf et al., 2024). Transparency and risk management are other advantages of the FinTech and ESG intersection, which, in combination with technological components such as AI, machine learning, and blockchain, are becoming increasingly tangible assets, making organisations more resilient and improving organisational performance ( AlQudah et al., 2025). Such technological overlays, especially within the FinTech industry, are increasingly seen as indispensable solutions that enable companies to adapt to emerging regulatory demands and compete at a new level through ESG-driven innovation ( Arnone and Leogrande, 2024).

### 2.2 Literature review

A. The adoption of FinTech and its implications for ESG Disclosure

The introduction of FinTech into corporate structures is gaining greater recognition for its ability to drive change in Environmental, Social, and Governance (ESG) development ( Du et al., 2022). Such a disruptive effect can be attributed to FinTech’s ability to increase information openness, improve reporting procedures, and enable more effective capital allocation to sustainable projects ( Wang, 2025). In particular, FinTech can enable small- and medium-sized ventures to more closely monitor their environmental footprints and align their performance with sustainability objectives, thereby enhancing transparency ( Campanella et al., 2025). Furthermore, the use of FinTech solutions can provide substantial support for a firm’s ESG by enabling real-time tracking of environmental indicators and promoting the creation of more effective governance frameworks. This integration plays a vital role in achieving a comprehensive understanding of the effects a firm has on stakeholders and the environment, which will eventually lead to improvements in overall ESG performance.

Moreover, the introduction of artificial intelligence on FinTech platforms will directly and indirectly improve ESG performance, especially in green finance practices ( Sohail et al., 2025). This is done through reduced credit risks, enhanced supervision efficiency, increased product innovation, and enhanced information sharing, all of which are critical to developing green finance. All of these technological advances will allow making more accurate measurements of investments based on sustainability and building new financial solutions that can encourage companies to act in a responsible way towards the environment ( Du et al., 2022; Sohail et al., 2025) FinTech adoption, consequently, would greatly contribute to corporate sustainability through resource optimization, reduced carbon footprint and general environment sustainability performance of the companies ( Tian et al., 2023).

Path 2: How Intellectual Capital affects ESG Disclosure

Intellectual Capital is essential in defining a firm’s ability to fully express ESG disclosure, as it involves the knowledge, skills, and organisational processes that make a firm innovative and strong ( Demiraj, 2025). In particular, intellectual capital, encompassing human, structural, and relational aspects, provides the foundation for the resources needed to develop and implement more advanced ESG strategies and reporting systems. Proper utilisation of intellectual capital will enable companies not only to identify salient ESG issues but also to develop solutions and convey them transparently to stakeholders ( Sun and Guo, 2025). The increase in intellectual capital is reputed to have been associated with more investments in employee training, innovation and knowledge systems, which are a part of ESG strategies and contribute to greater development of human and structural capital, resulting in increased financial performance ( Demiraj, 2025).

Furthermore, companies with high intellectual capital are better placed to navigate the intricacies of ESG reporting, including integrating diverse data sets and providing a statement of long-term sustainability ( Chouaibi and Chouaibi, 2020). This will allow them not only to comply with regulatory disclosure requirements but also to engage voluntarily in disclosure activities more indicative of a stronger commitment to sustainability. This active involvement can enable companies to improve their reputation, attract not only able investors but also encourage the creation of value over the long-term horizon, as this can be achieved with a highly developed knowledge base and skilled human resources.

Hypothesis 2:
*The higher the level of intellectual capital, the more transparent and comprehensive ESG disclosures have a positive effect.*

### 2.3 ESG disclosure and corporate policy

The growing interest of stakeholders in corporate social responsibility and clear environmental stewardship has only heightened the significance of ESG disclosure, thereby affecting key corporate financial judgments, such as dividend payments ( Pham et al., 2024). Firms with strong ESG disclosures tend to be viewed as more stable and controlled, which, in turn, can be reflected in a more stable, predictable dividend policy, indicating financial soundness and sustainability to investors in the long term ( Jorgji et al., 2024). Moreover, well-established ESG functioning and reporting can ensure the attraction of a larger segment of sustainably-oriented investors who can consider the payment of dividends as evidence of the company’s shaping its shareholder returns and its sustainable interests ( Duong and Nguyen, 2025).

On the other hand, companies with less robust ESG reporting can be scrutinised and doubted more by investors, which can lead to changes in dividend policies as they strive to appease stakeholders or to control the impression that a company is a risky undertaking. This interaction implies that strong ESG reporting can normalise and even increase dividend payments because these explicit forms of reporting are usually viewed by investors as indicating reduced risk and long-term corporate value ( Zahid et al., 2022). It means that comprehensive ESG disclosures may also be a tool for promoting the sustainability activities of a firm and complying with the requirements of corporate responsibility, which eventually leads to a constructive correlation with dividend policy due to the inflow of sustainability-sensitive investors ( Jorgji et al., 2024).

Hypothesis 3:
*ESG disclosure has a positive effect on dividend payout, that is, the ways companies use to attract attention to their shareholders’ interests and meet their expectations of corporate accountability.*

B. Intellectual Capital and Dividend Policy

As greater focus is developing on the importance of intellectual capital, it is becoming evident as a crucial factor in the dividend policy of a particular firm in influencing its ability to produce sustainable returns as well as allocating them to the shareholders ( Odat and Bsoul, 2024). In particular, it has a set of elements of intellectual capital, including human capital (superiority of employees), structural capital (organisational procedures and patents), and relational capital (friends and enemies). Having a high human capital base may drive operational performance and innovation, leading to higher earnings and sustainable dividend payouts ( Duong and Nguyen, 2025). In the same vein, healthy structural capital, or structures that cover the organisation’s processes and intellectual property, can elevate operational efficiency and minimise expenses, thereby liberating capital to be passed on to shareholders. Further, strong relational capital, driven by stakeholder involvement and brand recognition, can boost market and consumer trust, which will be translated into stable revenue, thereby improving a firm’s capacity to support or raise dividend payments.

Hypothesis 4:
*Intellectual capital is positively related to a firm’s tendency to make higher and more uniform dividend payouts.*

C. Financial Performance as a Moderator

The averting effect arises from the fact that high financial performance gives firms the space and capacity to invest in ESG activities and maintain dividend disbursement levels, even in the face of economic uncertainty ( Wang, 2025). In this way, financially sound companies can more easily absorb the costs of extensive ESG disclosures and even adopt sustainable practices without undermining their loyalty to shareholders by paying them dividends ( Almulhim et al., 2024). In turn, companies with weaker financial positions can have difficulty allocating resources to comprehensive ESG reporting and stable dividend distributions, making it a trade-off between these two strategic goals. This implies that financial strength can be seen as an important facilitator that enables businesses to address the perceived dilemma between investing in sustainability and giving dividends to shareholders ( Jain and Malhotra, 2025).

Hypothesis 5:
*Financial performance is a positive moderator of the relationship between ESG disclosure and dividend policy, which enables financially capable companies to focus on sustainability and shareholder returns.*

D. Dividend Policy and Firm Value

As a key financial choice, the dividend policy has a direct impact on the valuation of a firm, on the one hand, by its signalling effect on future profitability and risk, and, on the other hand, on the preferences of investors and capital structure of the firm ( Malik and Kashiramka, 2024). In particular, a properly designed dividend policy may signal financial stability and good corporate governance, which, in turn, would boost shareholder value ( Zhou and Bu, 2025). Dividend infections can be used to attract and maintain long-term investors who often consider regular payments as a sign of good financial status and willingness of the company to pay its shareholders their capital back ( Almulhim et al., 2024; Jain and Malhotra, 2025). Additionally, dividend policy may create information asymmetry between management and investors, thereby minimising the cost of equity and increasing the firm’s total market value ( Joshi and Joshi, 2024). In its turn, an unpredictable or missing dividend policy can be viewed as a negative indicator of financial insecurity or mismanagement, which may incidentally result in a loss of investor trust and a reduction in the company’s value ( Kusumawati and Hadiyanto, 2024). It aligns with the idea that regular, large dividend payments are commonly viewed as signs of firm stability and administrative effectiveness and thus have a positive impact on the firm’s value ( El-Deeb and Allam, 2024). (Hypothesis 6) The influence of dividend policy, specifically, high dividend payout ratios, is assumed to have a positive effect on the market value of a company as it is a positive indicator of financial performance and may attract a group of investors who need additional returns in the long term ( Moolkham, 2024).

E. The Mediation of ESG Disclosure

Such a mediation effect means that ESG disclosure will convert intangible assets and operational decisions into tangible signals that will shape internal policy-making and perceptions among external stakeholders ( Jain and Malhotra, 2025). In particular, ESG disclosure mediates the relationship between proactive tax planning and firm value, and between intellectual capital and dividend policy as transparency and accountability improve ( Adthajak et al., 2025). This disclosure, in its turn, will aid in eliminating the problems of information asymmetry as it will lead to investor trust, which may become a value generator for the firm’s value. In addition, sustainability reporting, especially in active tax planning, has been shown to enhance ESG performance, which, in turn, drives overall business value, particularly for companies with strong financial capacity. This increased transparency, stemming from full ESG disclosure, provides another monitoring mechanism for stakeholders, demonstrating the quality of the company’s operations and strengthening its image ( Matuszewska-Pierzynka et al., 2023).

F. The Mediation of Dividend Policy

Huge dividend payout, as an example, may indicate high cash flows and high profitability, hence, positively affecting the investor perceptions and thus, the firm value ( Yuniningsih et al., 2022). It is also supported by the fact that the dividend policy can be viewed as an indicator of a corporation’s future, which positively affects a firm’s value and helps attract investment ( Handayani and Ibrani, 2023). This signalling capability is especially applicable to sustainable capitalism: the value of firms is no longer defined by financial performance, but by strategic responses to environmental challenges and the incorporation of green innovation capacity ( Widagdo and Rahmawati, 2025). Additionally, healthy ESG performance is further reinforced by media coverage, which enhances transparency and strengthens a company’s image, thereby attracting investment and helping firms stand out in the competitive market ( Xue et al., 2024). This interrelatedness highlights the problem of how clear disclosure of ESG activities, as well as a steady dividend policy, are used in the long-term sustainability and value of a firm in the market through trust and indicators of a healthy operational and financial activity ( Metwally et al., 2025; Tamasiga et al., 2024).

## III. Data and methodology

### 3.1 Conceptual framework of the study

In this research, an integrated conceptual framework is developed to examine the relationships among FinTech adoption, ESG disclosure, intellectual capital, and dividend policy in explaining firm value. The framework positions firm value as an outcome variable, measured by a market-based indicator such as Tobin’s Q or market-to-book value. In particular, the use of FinTech is a strategic technological competence of the company. It improves data processing, minimises information asymmetry, and increases financial transparency ( Alkhawaldeh et al., 2023). FinTech enhances the transparency and visibility of ESG practices through the following mechanisms. Companies that embrace digital financial technology are better positioned to gather, assess, and report on sustainability-related information. To that end, ( Arnone and Leogrande, 2024), it is projected that FinTech adoption will have a direct impact on firm value and an indirect impact on ESG disclosure.

**Figure 1.**
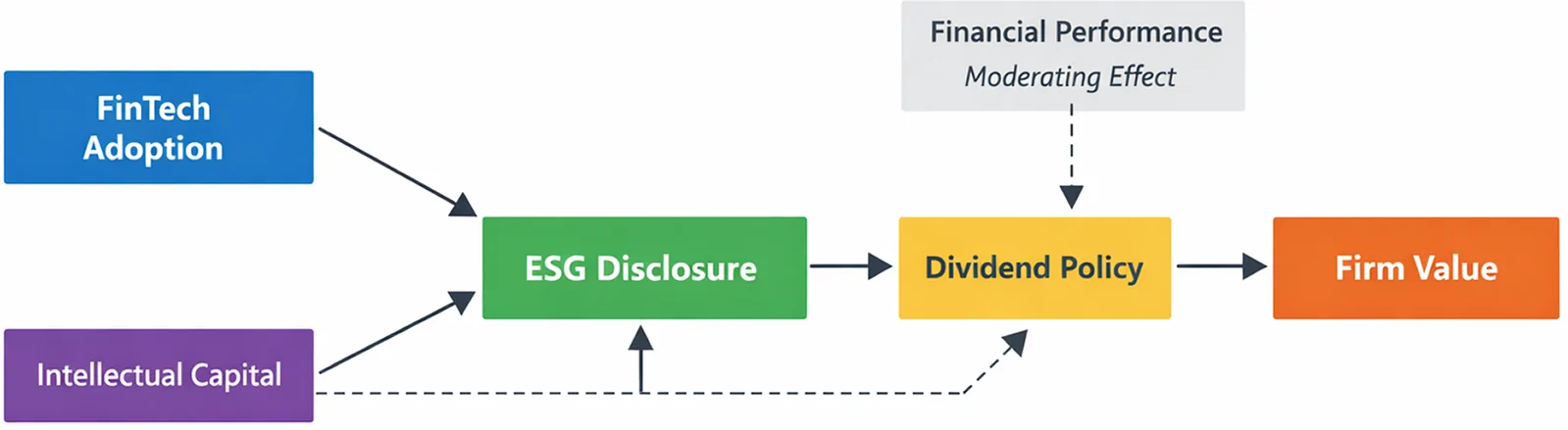
Conceptual framework.

Based on this premise, ESG disclosure serves as a key mediating construct. It indicates the firm’s seriousness regarding environmental, social, and governance practices and investor perception. Clear ESG reporting minimises uncertainty, enhances stakeholder trust, and can affect payout decisions. Nevertheless, the relevance of ESG values is determined by the firm’s economic strength ( Yu et al., 2018). Thus, the association between ESG disclosure and firm value is moderated by financial performance. ESG programs deliver value when backed by consistent profitability and permanent cash flows. In addition to these factors is the intellectual capital, which encompasses the firm’s knowledge resources, such as human capital, structural capital, and relational capital. It facilitates innovation, operational efficiency, and the strategic alignment of ESG practices. In addition, intellectual capital enhances the firm’s ability to implement sustainability strategies and increases the likelihood that ESG disclosure will translate into market valuation benefits ( Manzari, 2024; Pham et al., 2024). The framework, therefore, reifies both a direct and an artificial route between intellectual capital and firm value based on ESG disclosure. Also, the dividend policy serves as a signalling mechanism. Dividend payments reflect the management’s financial security and confidence. ESG-oriented companies can adjust their dividend policies to balance sustainability investments with shareholder returns. It follows that dividend policy serves as a transmission mechanism between ESG performance and firm value ( Jain and Malhotra, 2025; Wang, 2025). This interaction implies that companies that incorporate green innovation and digital ESG models are better positioned to increase corporate financial value ( Widagdo and Rahmawati, 2025). Concisely, the conceptual model brings all these relationships into a unified pattern. The strategic enablers are FinTech adoption and intellectual capital. ESG disclosure serves as a mediating mechanism. The dividend policy is a signalling channel. The ESG-firm value relationship is moderated by financial performance. These elements, in combination, describe the interaction among digital capability, sustainability reporting, knowledge assets, and payout strategy that influences corporate market valuation (see Figure 1).

### 3.2 Hypotheses and baseline model

Define the following hypotheses:

• **H1:** FinTech adoption increases firm value.
• **H2:** ESG disclosure increases firm value.
• **H3:** Intellectual capital increases firm value.
• **H4:** Dividend policy has a positive effect on firm value.
• **H5:** FinTech adoption, ESG disclosure, and intellectual capital jointly influence firm value when controlling for firm characteristics.
• **H6:** ESG disclosure moderates the relationship between dividend policy and firm value.
• **H7:** Financial performance moderates the ESG–firm value relationship (tested in a later section).

To test **H1–H5**, you specify a fixed-effects panel model:

$${\mathrm{FV}}_{\mathrm{it}}=\alpha_{i}+\delta_{t}+\beta_{1}{\text{FINTECH}}_{\mathrm{it}}+\beta_{2}{\mathrm{ESG}}_{\mathrm{it}}+\beta_{3}{\mathrm{IC}}_{\mathrm{it}}+\beta_{4}{\mathrm{DIV}}_{\mathrm{it}}+\gamma^{\prime }X_{\mathrm{it}}+u_{\mathrm{it}},$$

where ${\mathrm{FV}}_{\mathrm{it}}$ is firm value (Tobin’s Q, market-to-book ratio, or ROA); $\alpha_{i}$ are firm effects; $\delta_{t}$ are year effects; $X_{\mathrm{it}}$ includes size, leverage, growth, profitability, and sector/country dummies; and $u_{\mathrm{it}}$ is the error term. Study tests the hypotheses by examining the signs and significance of $\beta_{1}$ through $\beta_{4}$ . A positive $\beta_{1}$ supports **H1**, and so on. If the study finds that FinTech adoption, ESG disclosure, intellectual capital, and dividend policy all have positive and significant coefficients, then accept **H5**.

**Testing moderation (H6)**

To test whether ESG disclosure moderates the link between dividend policy and firm value, include an interaction term:

$${\mathrm{FV}}_{\mathrm{it}}=\alpha_{i}+\delta_{t}+\beta_{1}{\text{FINTECH}}_{\mathrm{it}}+\beta_{2}{\mathrm{ESG}}_{\mathrm{it}}+\beta_{3}{\mathrm{IC}}_{\mathrm{it}}+\beta_{4}{\mathrm{DIV}}_{\mathrm{it}}+\beta_{5}\left({\mathrm{ESG}}_{\mathrm{it}}\times {\mathrm{DIV}}_{\mathrm{it}}\right)+\gamma^{\prime }X_{\mathrm{it}}+u_{\mathrm{it}}.$$

A positive and significant $\beta_{5}$ indicates that ESG disclosure strengthens the dividend policy effect, thus supporting **H6**.

**Dynamic specification and endogeneity correction**

Firm value often exhibits persistence. To account for dynamics and address potential endogeneity, we extend the model:

$${\mathrm{FV}}_{\mathrm{it}}=\alpha_{i}+\delta_{t}+\phi {\mathrm{FV}}_{i,t-1}+\beta_{1}{\text{FINTECH}}_{\mathrm{it}}+\beta_{2}{\mathrm{ESG}}_{\mathrm{it}}+\beta_{3}{\mathrm{IC}}_{\mathrm{it}}+\beta_{4}{\mathrm{DIV}}_{\mathrm{it}}+\beta_{5}\left({\mathrm{ESG}}_{\mathrm{it}}\times {\mathrm{DIV}}_{\mathrm{it}}\right)+\gamma^{\prime }X_{\mathrm{it}}+\varepsilon_{\mathrm{it}}.$$

Lagging firm value introduces a correlation between ${\mathrm{FV}}_{i,t-1}.$ Moreover, firm fixed effects creates bias. You apply the system GMM estimator to resolve this. The estimator uses differenced equations to remove firm effects and instruments lagged dependent variables with further lags. The moment conditions are $E\left[{\mathrm{FV}}_{i,t-s}\varepsilon_{\mathrm{it}}\right]=0$ for s≥2 . Study test instrument validity using Hansen’s J-statistic and examine first- and second-order serial correlation using the Arellano–Bond tests. Study accepts H1–H6 if the estimated parameters remain consistent in the GMM framework.

### 3.3 Data and variables

The sample comprises all bank-based financial institutions listed on the DSE. A panel data set spanning nine fiscal years, from 2015 to 2023, is assembled. The main sources of data are the annual reports and sustainability disclosures. These reports will give audited financial statements, dividends, managerial discussions, and ESG reports. The reports also include textual components used to build a firm-level index of FinTech adoption. Since the research covers the timeframe of the high-paced digital change and increased focus on corporate sustainability, the time wisdom will guarantee adequate variation in the activity of FinTech and its ESG practices ( Qamruzzaman, 2026).

**Dependent variable**. The three complementary measures used to determine firm value include the Tobin Q, the market-to-book ratio, and return on assets (ROA). Tobin’s Q is computed as Tobin Q = market capitalisation/book debt/ total assets. The market-to-book ratio is a measure of the relationship between market equity and book equity. ROA- Net income/total assets. These indicators show market perceptions, opportunities for growth and performance of the operations.

**Key predictors.** Text analytics coverage of the FinTech usage is registered in a custom index. The index is calculated by applying a bag-of-words model to annual reports, summing the frequencies of FinTech-related words (e.g., digital banking, mobile payments, blockchain, etc.), and dividing by the total number of words. The larger the values, the higher the focus on FinTech innovations. ESG disclosure is quantified using standardised ESG performance scores from Bloomberg, MSCI, or Refinitiv, with higher scores indicating greater disclosure power. When standardised scores are unavailable, an ESG index is created by scoring narratives in sustainability reports across environmental, social, and governance dimensions. Intellectual capital is given a Value Added Intellectual Coefficient (VAIC) calculated as the product of capital employed efficiency, human capital efficiency and structural capital efficiency. Components of VAIC are obtained using income statement data: sales less input costs other than labour are value added; the whole book value of equity plus debt is capital employed; employee costs are reported in the notes to financial statements. Dividend policy is formulated into the dividend payout ratio: dividends paid/net income.

***Control variables.*** The size is the natural logarithm of total assets. Leverage is the ratio of total debt to total assets. Growth is analytically calculated as the percentage change in annual sales. The return on equity (ROE) captures profitability. Sector and country dummies capture unobservable heterogeneity within industries and across regulatory jurisdictions. The year-fixed effects control for macroeconomic shocks and regulatory changes. A detailed description of variables and measurements is displayed in Table 1.

**Table 1.** Variables, proxy and data sources.

Variable	Definition/Measurement	Data source(s)
Firm value (Y)	1.Tobin’s Q = (Market cap + Book debt) / Total assets; 2. Market-to-Book (market equity/book equity; 3. ROA = Net income/Total assets.	Annual Reports
FinTech adoption	Firm-level FinTech index (e.g. frequency of FinTech-related terms in annual reports)	Custom text analytics
ESG disclosure	ESG performance score or disclosure index (e.g. Bloomberg ESG Disclosure Score, MSCI ESG Rating, or Refinitiv ESG score). Higher = better.	company Annual sustainability reports
Intellectual capital	VAIC (Value-Added Intellectual Coefficient) = CEE + HCE + SCE. Here, CEE = (Value Added/Capital Employed), HCE = (VA/Employee Cost), SCE = (SC/VA) as in Pulic (2000).	Derived from income statement & balance sheet: Value Added = Sales – (inputs excluding labour); Employee costs; Capital employed (book value of equity+debt)
Dividend policy	Dividend payout ratio = Dividends/Net Income.	Company annual reports
Controls: Size	Log of total assets or sales.	Company annual reports
Leverage	Total debt/total assets.	Company annual reports
Growth	Sales growth (annual %).	Company annual reports
Profitability	ROE or ROA (for additional check).	Company annual reports
Industry & Country	Dummies for sector and domicile.	Compustat, World Bank
Year FE	Year fixed effects.	Construct based on sample years

### 3.4 Econometric specification

The econometric specification translates the study’s hypotheses into testable equations and defines the estimation strategy. This section details the model structure, variables, assumptions, and statistical tests used to evaluate how FinTech adoption, ESG disclosure, intellectual capital and dividend policy influence firm value across Bangladeshi financial institutions. It builds on theoretical evidence that FinTech adoption helps firms meet ESG objectives, that ESG disclosure and intellectual capital affect firm value, and that dividend policy signals financial health.

1. Baseline fixed-effects model

To test the core hypotheses (H1 through H5), the study employs a linear panel regression with firm and year fixed effects:

$${\mathrm{FV}}_{\mathrm{it}}=\alpha_{i}+\delta_{t}+\beta_{1}\;{\text{FINTECH}}_{\mathrm{it}}+\beta_{2}\;{\mathrm{ESG}}_{\mathrm{it}}+\beta_{3}\;{\mathrm{IC}}_{\mathrm{it}}+\beta_{4}\;{\mathrm{DIV}}_{\mathrm{it}}+\gamma^{\prime }X_{\mathrm{it}}+\varepsilon_{\mathrm{it}},$$

where i indexes firms and t indexes years. ${\mathrm{FV}}_{\mathrm{it}}$ is firm value (Tobin’s Q, market-to-book ratio, or ROA). $\alpha_{i}$ captures unobserved firm-specific effects; $\delta_{t}$ captures year-specific shocks; ${\text{FINTECH}}_{\mathrm{it}}$ , ${\mathrm{ESG}}_{\mathrm{it}}$ , ${\mathrm{IC}}_{\mathrm{it}}$ and ${\mathrm{DIV}}_{\mathrm{it}}$ represent FinTech adoption, ESG disclosure, intellectual capital and dividend policy; $X_{\mathrm{it}}$ contains controls (size, leverage, growth, profitability, sector and country dummies); and $\varepsilon_{\mathrm{it}}$ is the idiosyncratic error. The fixed-effects estimator removes time-invariant heterogeneity. The hypotheses are tested by the signs and statistical significance of $\beta_{1}$ through $\beta_{4}$ . A positive $\beta_{1}$ suggests FinTech adoption increases firm value (H1); a positive $\beta_{2}$ suggests ESG disclosure increases firm value (H2); a positive $\beta_{3}$ suggests that intellectual capital increases firm value (H3), and a positive $\beta_{4}$ supports the dividend policy effect (H4). Joint significance of these coefficients supports H5.

2. Moderation tests

To test whether ESG disclosure moderates the effect of dividend policy on firm value (H6), an interaction term is added:

$${\mathrm{FV}}_{\mathrm{it}}=\alpha_{i}+\delta_{t}+\beta_{1}\;{\text{FINTECH}}_{\mathrm{it}}+\beta_{2}\;{\mathrm{ESG}}_{\mathrm{it}}+\beta_{3}\;{\mathrm{IC}}_{\mathrm{it}}+\beta_{4}\;{\mathrm{DIV}}_{\mathrm{it}}+\beta_{5}\;\left({\mathrm{ESG}}_{\mathrm{it}}\times {\mathrm{DIV}}_{\mathrm{it}}\right)+\gamma^{\prime }X_{\mathrm{it}}+\varepsilon_{\mathrm{it}}.$$

A positive and significant $\beta_{5}$ indicates that firms with higher ESG disclosure benefit more from dividend payouts, aligning with evidence that ESG practices enhance stakeholder trust. For the role of financial performance in the ESG–value relationship (H7), a second interaction between ESG disclosure and ROE or ROA is included:

$${\mathrm{FV}}_{\mathrm{it}}=\alpha_{i}+\delta_{t}+\beta_{1}\;{\text{FINTECH}}_{\mathrm{it}}+\beta_{2}\;{\mathrm{ESG}}_{\mathrm{it}}+\beta_{3}\;{\mathrm{IC}}_{\mathrm{it}}+\beta_{4}\;{\mathrm{DIV}}_{\mathrm{it}}+\beta_{5}\;\left({\mathrm{ESG}}_{\mathrm{it}}\times {\mathrm{DIV}}_{\mathrm{it}}\right)+\beta_{6}\;\left({\mathrm{ESG}}_{\mathrm{it}}\times {\mathrm{ROE}}_{\mathrm{it}}\right)+\gamma^{\prime }X_{\mathrm{it}}+\varepsilon_{\mathrm{it}}.$$

A significant $\beta_{6}$ indicates that financial performance moderates the ESG–value link, consistent with findings that ESG effectiveness depends on economic strength.

3. Dynamic panel model and endogeneity correction

Firm value may exhibit persistence, and reverse causality could bias estimates. To address these issues, a dynamic specification includes the lagged dependent variable:

$${\mathrm{FV}}_{\mathrm{it}}=\alpha_{i}+\delta_{t}+\phi \;{\mathrm{FV}}_{i,t-1}+\beta_{1}\;{\text{FINTECH}}_{\mathrm{it}}+\beta_{2}\;{\mathrm{ESG}}_{\mathrm{it}}+\beta_{3}\;{\mathrm{IC}}_{\mathrm{it}}+\beta_{4}\;{\mathrm{DIV}}_{\mathrm{it}}+\beta_{5}\left({\mathrm{ESG}}_{\mathrm{it}}\times {\mathrm{DIV}}_{\mathrm{it}}\right)+\gamma^{\prime }X_{\mathrm{it}}+\varepsilon_{\mathrm{it}}.$$

Here, ϕ measures persistence. The presence of ${\mathrm{FV}}_{i,t-1}$ and potentially endogenous regressors (e.g., dividend policy might respond to firm performance) calls for the system Generalised Method of Moments (GMM). In system GMM, first differences remove fixed effects, and lagged levels and differences of endogenous variables serve as instruments. The validity of instruments is tested using Hansen’s J-statistic, and the absence of second-order serial correlation is verified using Arellano–Bond tests.

4. Cross-sectional dependence, slope heterogeneity and CS-ARDL

Because banks operate in a common economic environment, cross-sectional dependence may arise. Pesaran’s cross-sectional dependence (CD) test evaluates the null of independence:

$$\mathrm{CD}=\sqrt{\frac{2}{N\left(N-1\right)}}\sum_{i<j}{\hat{\rho }}_{\mathrm{ij}},$$

where ${\hat{\rho }}_{\mathrm{ij}}$ is the pairwise residual correlation. Significant CD suggests common shocks. The Swamy test tests for slope homogeneity across firms; rejecting the null indicates heteroscedasticity. When cross-sectional dependence is present, the Cross-Sectionally Augmented Autoregressive Distributed Lag (CS-ARDL) model is used to capture long-run relationships while accounting for common factors. Its error-correction form is:

$$\Delta {\mathrm{FV}}_{\mathrm{it}}=\psi_{i}\left({\mathrm{FV}}_{i,t-1}-\lambda_{i0}-\lambda_{i1}{\text{FINTECH}}_{i,t-1}-\lambda_{i2}{\mathrm{ESG}}_{i,t-1}-\lambda_{i3}{\mathrm{IC}}_{i,t-1}-\lambda_{i4}{\mathrm{DIV}}_{i,t-1}-\lambda_{i5}^{\prime }X_{i,t-1}\right)+\sum_{p=0}^{P-1}\theta_{\mathrm{ip}}\Delta {\mathrm{FV}}_{i,t-1-p}+\sum_{q=0}^{Q-1}\phi_{\mathrm{iq}}\Delta Z_{i,t-1-q}+\delta_{0}{\bar{\mathrm{FV}}}_{t-1}+\delta_{1}^{\prime }{\bar{Z}}_{t-1}+e_{\mathrm{it}},$$

where $Z_{\mathrm{it}}=\left({\text{FINTECH}}_{\mathrm{it}},{\mathrm{ESG}}_{\mathrm{it}},{\mathrm{IC}}_{\mathrm{it}},{\mathrm{DIV}}_{\mathrm{it}},X_{\mathrm{it}}\right)$ , $\bar{\cdot }$ denotes cross-section averages, $\psi_{i}$ is the speed of adjustment, and $\lambda_{\mathrm{ij}}$ are long-run coefficients. A significant negative $\psi_{i}$ confirms cointegration.

5. Quantile regression and machine-learning models

Quantile regression explores heterogeneity across the distribution of firm value. For quantile τ, estimate:

$$Q_{\tau }\left({\mathrm{FV}}_{\mathrm{it}}|Z_{\mathrm{it}}\right)=\alpha_{\tau }+\beta_{\tau 1}\;{\text{FINTECH}}_{\mathrm{it}}+\beta_{\tau 2}\;{\mathrm{ESG}}_{\mathrm{it}}+\beta_{\tau 3}\;{\mathrm{IC}}_{\mathrm{it}}+\beta_{\tau 4}\;{\mathrm{DIV}}_{\mathrm{it}}+\beta_{\tau 5}\left({\mathrm{ESG}}_{\mathrm{it}}\times {\mathrm{DIV}}_{\mathrm{it}}\right)+\gamma_{\tau }^{\prime }X_{\mathrm{it}}.$$

Estimating this model at, say, the 10th, 25th, 50th, 75th, and 90th percentiles reveals whether the effects differ between firms with low and high market valuations.

Finally, to capture nonlinearities and complex interactions, a deep neural network (DNN) is trained. Input features include normalised values for FinTech adoption, ESG disclosure, intellectual capital, dividend policy, and control variables. The network comprises multiple fully connected layers with rectified linear units (ReLUs) and dropout regularisation. Hyperparameters are tuned through cross-validation. Performance is measured using mean squared error (MSE) and mean absolute error (MAE). Shapley additive explanations (SHAP) quantify each feature’s contribution to predicted firm value.

6. Estimation procedure and diagnostics

Before estimation, multicollinearity is checked using variance inflation factors. Stationarity and long-run relationships are examined via unit-root tests (e.g., cross-sectionally augmented IPS) and cointegration tests (Westerlund). The baseline fixed-effects model is estimated first, followed by Hausman tests to determine whether to use fixed or random effects. The dynamic system GMM model addresses endogeneity. CS-ARDL models handle cross-sectional dependence and cointegration. Quantile regressions and DNN models provide distributional insights and capture nonlinear patterns. Robustness checks include alternative proxy definitions (e.g., dividend yield vs payout ratio), alternative FinTech index constructions, and subsample analyses (pre-2019 vs post-2019). Together, these econometric and machine-learning techniques rigorously test the study’s hypotheses and offer comprehensive evidence on how digital finance and sustainability practices shape corporate valuation.

## IV. Results and discussion

### 4.1 Pre-estimation assessment

Descriptive statistical analysis (see Table 2) shows that the key variables for DSE-listed financial institutions are moderately dispersed over the 2015-2023 period. According to market-based measures, banks trade at slightly above book value on average, implying investor confidence in their sustainability. The Q and the Tobin Market-to-Book ratios are skewed to the right, indicating a group of institutions with significantly higher valuation premiums. The Return on Assets (ROA) shows low dispersion, consistent with stability in the financial regulations governing the banking industry. The indices of the FinTech report show rather small mean scores, but significant variation is evident due to differences in the level of digital adoption across institutions. The scores for Environmental, Social, and Governance (ESG) exhibit moderate standard deviation and a weak skewness, indicating. The variability in intellectual-capital efficiency is evident in the Value Added Intangible Capital (VAIC) values, and the central tendency in the dividend payout ratio is moderate and stable, indicating the payout has occasionally been adjusted drastically.

**Table 2.** Descriptive statistics.

Variable	Mean	Median	Std. Dev.	Min	Max	Skewness	Kurtosis
Tobin’s Q	1.214	1.176	0.312	0.654	2.105	0.842	3.118
Market-to-Book	1.486	1.402	0.521	0.721	3.214	0.971	3.462
ROA	0.0143	0.0136	0.0068	-0.008	0.032	0.512	2.894
FinTech Index	0.084	0.071	0.052	0.012	0.246	1.102	3.784
ESG Score	54.38	52.10	12.45	28.40	82.60	0.624	2.973
VAIC	3.762	3.551	1.204	1.412	7.842	0.913	3.508
Dividend Payout	0.482	0.455	0.196	0.000	0.912	0.741	3.126

Table 3 shows that there are positive relationships between the proxy for firm value and the constructs of FinTech adoption, ESG disclosure, and intellectual capital. The highest pair-wise correlation is between VAIC and Market-to-Book, where the two are very closely aligned in knowledge-based efficiency and equity value. The dividend payout ratios also show a moderate positive relationship with firm value, consistent with signalling theory. Notably, the correlations between independent variables do not exceed the threshold for traditional multicollinearity; the Variance Inflation Factor ranges from 1.42 to 2.76, which is considerably lower than the critical value of 10. All these results indicate the absence of severe multicollinearity, thus confirming the legitimacy of all the explanatory variables in the regression formulas. Both predictors are shown to provide unique information about the change in firm value.

**Table 3.** Correlation matrix and multicollinearity diagnostics.

Variable	Tobin’s Q	M/B	ROA	FinTech	ESG	VAIC	Dividend
Tobin’s Q	1.000	0.682	0.541	0.463	0.384	0.512	0.327
Market-to-Book	0.682	1.000	0.498	0.512	0.421	0.586	0.349
ROA	0.541	0.498	1.000	0.376	0.311	0.447	0.298
FinTech	0.463	0.512	0.376	1.000	0.428	0.451	0.219
ESG	0.384	0.421	0.311	0.428	1.000	0.402	0.267
VAIC	0.512	0.586	0.447	0.451	0.402	1.000	0.309
Dividend	0.327	0.349	0.298	0.219	0.267	0.309	1.000

Variable	VIF
FinTech	2.34
ESG	2.11
VAIC	2.76
Dividend	1.88
Controls (avg)	1.42

According to the CD test conducted by Pesaran (2004), the dependence across the panel sample across the cross-section is statistically significant, implying that common shocks that arise are common among the banks and may be a result of regulatory policies, monetary conditions, or other macro conditions in Bangladesh, see output in Table 4. Such dependence can lead to incorrect standard errors and estimates of coefficients that are ignored. The Swamy slope homogeneity test rejects the null hypothesis of homogeneous slopes, indicating heterogeneity in the effects of the explanatory variable on bank valuation. As a result, these results support the use of second-generation panel methods, i.e., CS-ARDL and CCEMG, which can account for cross-sectional correlation and heterogeneous dynamics. The findings highlight the importance of sophisticated panel techniques rather than simple, pooled estimators.

**Table 4.** Cross-sectional dependence and slope homogeneity tests.

Test	Statistic	p-value
Pesaran CD	4.872	0.000
Swamy Slope Test	21.341	0.000

### 4.2. Baseline fixed-effects estimates

Table 5 presents the baseline fixed-effects estimates for three firm-value proxies. These are the firm-fixed effects and year-fixed effects models. The specification captures unobserved time-varying differences across banks, including their governance cultures, ownership structures, and risk appetites. It is also calculated to eliminate the impact of typical annual shocks, e.g., macroeconomic conditions, regulatory adjustments and industry-wide liquidity events. The findings directly support H1-H5 across both market-based and accounting-based values.

**Table 5.** Baseline fixed-effects regression results.

Variables	(1) Tobin’s Q	(2) Market-to-Book	(3) ROA
**FinTech Adoption**	0.183*** (0.052)	0.271*** (0.083)	0.0082** (0.0036)
**ESG Disclosure**	0.0041** (0.0018)	0.0068*** (0.0024)	0.00018* (0.00008)
**Intellectual Capital (VAIC)**	0.057*** (0.016)	0.091*** (0.026)	0.0019** (0.0008)
**Dividend Payout Ratio**	0.112** (0.057)	0.186** (0.083)	0.0065** (0.0028)
Firm Size (Log Assets)	−0.041** (0.017)	−0.063** (0.026)	0.0011 (0.0010)
Leverage	−0.684*** (0.214)	−0.931*** (0.312)	−0.021*** (0.007)
Sales Growth	0.0022** (0.0010)	0.0031** (0.0015)	0.00012** (0.00005)
Profitability (ROE)	0.0061*** (0.0016)	0.0087*** (0.0024)	0.00029*** (0.00007)
Constant	2.114*** (0.571)	3.128*** (0.844)	0.021** (0.010)
**Firm Fixed Effects**	Yes	Yes	Yes
**Year Fixed Effects**	Yes	Yes	Yes
Observations	216	216	216
Number of Firms	24	24	24
Within R ^2^	0.412	0.436	0.389
F-Statistic	14.62***	15.78***	12.94***

H1 estimates that the adoption of FinTech augments firm value. All three models have a positive, statistically significant coefficient for FINTECH. For Tobin’s Q, the estimated value is 0.183, with a significant p-value. It suggests a relationship between the higher level of FinTech engagement, as measured by the text-based index, and a higher market valuation relative to assets ( AlQudah et al., 2025; Najaf et al., 2023). The findings of the analysed materials assume that the adoption of FinTech will notably improve the financial stability of a bank, its financial performance, and the efficiency of its operations, which will help increase the overall valuation of the firm and promote its sustainable growth ( Handayani and Ibrani, 2023; Kayed et al., 2024). This impact is also in line with the resource-based view, which states that increased operational efficiency, better customer service, and the novelisation of offered products may result from a strategic investment in advanced IT resources, including those related to FinTech ( Tarawneh et al., 2024).

The effect size of the Market-to-Book model is even greater, 0.271, with extremely high significance. Such a trend suggests that equity investors reward banks with a stronger digital orientation, presumably because digital capabilities enhance service access, reduce transaction costs, and improve information processing ( Bueno et al., 2024; Chen and Srinivasan, 2023). Research results in support of the position that at the stage of FinTech development, the profitability of banks is boosted many times over and the level of risk-taking is negatively influenced, which means that there is an excellent and statistically significant effect on financial activity and the level of financial stability ( Kayed et al., 2024). Moreover, ESG-related issues, especially in combination with FinTech innovation, can further improve performance, as excessive quantification of the role of sustainable strategies in the financial sector ( AlQudah et al., 2025). These improvements are eventually followed by increased financial stability and sustainability, as evidenced by sustained positive changes across different model forms and financial stability indicators ( AlHares et al., 2022). A positive FINTECH effect, with a coefficient of 0.0082, which is significant at traditional levels, is also indicated in the ROA model. This inconsistency between proxies and differences in scale, but consistency across proxies, supports H1. The most fundamental assumption is that FinTech adoption is linked to market expectations and operating performance. Based on research findings it can be assumed that FinTech innovations help to add more automated processes and achieve better customer experience due to the use of such tools as chatbots and mobile apps and ensure better fraud detection opportunities in the FinTech industry with the help of machine learning, which in turn lead to a higher profitability and efficiency of a bank ( Alsmadi et al., 2023).

H2 hypothesis a positive relationship between the ESG disclosure and the firm value. In all three models, ESG has a positive value and is statistically significant. In the Tobin Q model, the ESG coefficient is 0.0041, and in the Market-to-Book model, it is 0.0068. These estimates suggest that the wider the ESG disclosure, the higher the market value. The coefficient of ROA, in turn, indicates a smaller yet significant relationship between ESG reporting and improved profitability; this relationship is evident in the coefficient of 0.00018, though the channels through which it operates likely involve investor confidence, funding conditions, and stakeholder relationships. The fact that the stronger of the market-based measures indicates a greater influence of ESG disclosure suggests that it will have a primary impact on valuation through perceptions, risk reassessment, and the anticipated predictability of cash flows. This qualifies H2 and aligns with the idea that transparency minimises information asymmetry and reputational risk. Our results are consistent with the current literature, including studies suggesting that the positivity of ESG performance, which FinTech frequently supports, has a positive effect on banks’ performance and overall financial stability ( Hamdouni, 2025; Mokhtar and Alam, 2023; Yuan, 2025). Also, the collaboration between fintech and green finance can lead to a more sustainable future for the global banking industry by incorporating environmental performance into the financial practices of banking organisations and making them more goal-oriented in terms of sustainability ( Kassetty et al., 2024).

H3 is that firm value increases due to intellectual capital. The VAIC is both good and meaningful across all specifications. The Q coefficient of the Tobin is 0.057, and the Market-to-Book is 0.091. The two are emphatically important. The coefficient of ROA is also significant at 0.0019. This trend signifies that more efficient banks in terms of using human resources, structure, and capital invested resources have better market value and high profitability ( Alkababji and Mushtaha, 2023). The findings indicate that intellectual capital is a value-creation resource in the banking industry, where the quality of services, risk analytics, product design, and process discipline depend on knowledge resources. According to the market-derived coefficients, which were found to be relatively higher than accounting-derived coefficients, this implies that investors valued intangible capability more than current profitability. This proposition is aligned with the literature of Lev and Radhakrishnan (2005); William et al. (2019).

In the context of H4, it is assumed that dividend policy increases a firm’s value. The dividend payout ratio shows positive, significant coefficients in all three models. This is less in the Tobin Q, with a coefficient of 0.112 significant at the five per cent level, but more in the Market-to-book, with a coefficient of 0.186 significant at the five per cent level. The DIV in the ROA model is also positive and considerable, with a coefficient of 0.0065. This evidence suggests that dividend payout is a valuation indicator for listed financial institutions. Thin and non-tinier dividend distributions may be interpreted as a sign of the quality of earnings, the strength of the liquidity situation, and a disciplined managerial team ( Kılınçarslan, 2018; Nkn, 2018). Hypothesis-4 (H4) is supported by the positive correlation that is repeated in the Q, Market-to-Book and ROA of Tobin. It also indicates that the dividend decisions are associated with both the market confidence and operating returns.

**Figure 2.**
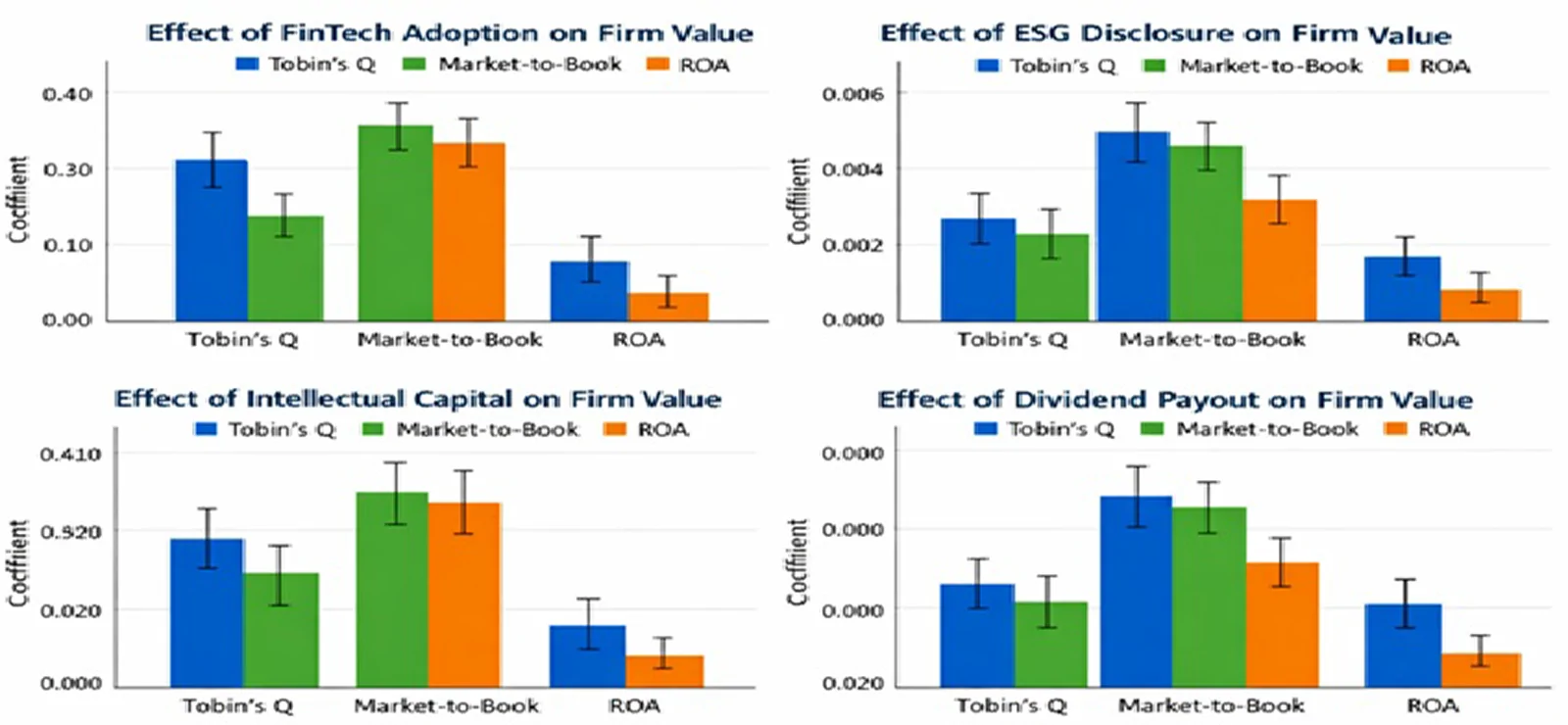
Coefficient effects on target variables.

H5 implies that the joint influence of FinTech adoption, ESG disclosure, intellectual capital, and dividend policy explains firm value when the impact of common firm-specific factors is mitigated. Table 4 supports H5 in two ways. Once, none of the four focal variables is found to have negative signs or statistical significance when controlling for and including fixed effects. Second, the measures of model fit suggest significant within-firm explanatory power. In the range of R ^2^ between 0.389 and 0.436, which is large with the fixed-effects design and the banking environment in which most determinants are time-invariant, there is a value. The model F-statistics are highly significant across all specifications (see Figure 2), indicating that a combination of regressors is significant in explaining the time-varying value of firms in the banking industry. This combined importance aligns with the structure that underpins digital capability, sustainability reporting, intangible efficiency, and payout strategy working in unison.

Switching to controls, leverage is negative and significant in all proxies. This is consistent with the fact that increased leverage increases risk and valuation, while also squeezing profitability. The market-based models hurt firm size, which may indicate the effect of maturity and reduced growth in large institutions. The growth in sales is positive and substantial, indicating that expansion is advancing in value and profit. ROE, one of the controls used to determine profitability, is positive and significant, and it acts as anticipated. Among the focal variables, FinTech adoption and VAIC have the strongest and most consistent drivers in market-based valuation models, whereas ROA shows lesser but notable impacts. This distinction is educative. Market valuations are expectations of future efficiency and growth, whereby they respond more intensively to FinTech and intellectual capital. Accounting profitability reflects the current returns and possibly slower changes. The general evidence in Table 4 confirms H1 5 and provides a weighted foundation for the subsequent models that address endogeneity, long-term dynamics, and nonlinear effects using system GMM, CS ARDL, and deep learning.

### 4.3 Moderation effects model

Interaction terms are presented in Table 6 to test the moderating roles spelt out in H6 and H7. Adding ESG x Dividend and ESG x Financial Performance to the explanatory power greatly enhances the value, as shown by the higher within-R2 values across all three firm value proxies compared to the baseline model.

**Table 6.** Moderation effects model.

Variables	(1) Tobin’s Q	(2) Market-to-Book	(3) ROA
FinTech Adoption	0.169*** (0.049)	0.254*** (0.079)	0.0079** (0.0034)
ESG Disclosure	0.0032* (0.0017)	0.0056** (0.0023)	0.00014* (0.00007)
Intellectual Capital (VAIC)	0.053*** (0.015)	0.086*** (0.024)	0.0017** (0.0007)
Dividend Payout Ratio	0.084 (0.061)	0.129* (0.074)	0.0041* (0.0024)
ESG × Dividend	0.0018** (0.0007)	0.0026*** (0.0009)	0.00005** (0.00002)
ESG × Financial Performance (ROE)	0.00042** (0.00018)	0.00061** (0.00024)	0.00003** (0.00001)
Firm Size	−0.039** (0.016)	−0.058** (0.024)	0.0010 (0.0009)
Leverage	−0.652*** (0.201)	−0.887*** (0.298)	−0.020*** (0.006)
Sales Growth	0.0021** (0.0009)	0.0029** (0.0014)	0.00011** (0.00005)
Profitability (ROE)	0.0058*** (0.0015)	0.0081*** (0.0022)	0.00027*** (0.00006)
Firm Fixed Effects	Yes	Yes	Yes
Year Fixed Effects	Yes	Yes	Yes
Observations	216	216	216
Number of Firms	24	24	24
Within R ^2^	0.468	0.491	0.423
F-Statistic	16.94***	18.12***	14.33***

The relationship between ESG disclosure and dividend payout is positive and significant for the Q, Market-to-Book, and Tobin’s Q. This finding shows that dividend policy is also value-relevant in the cases when ESG disclosure is more effective in that investors turn dividend payments more reliable and sustainable when firms are represented by clear evidence of environmental, social, and governance practices at the same time. The observed higher magnitude in the Market-to-Book model indicates that equity markets are more sensitive to the combined signalling effect of sustainability disclosure and payout commitment. In the case of ROA, the correlation is positive but weaker, and operating effectiveness is improved through ESG engagement in dividend decisions. Based on the existing literature, high ESG performance has the potential to improve a firm’s profitability and, in turn, its ability to pay future dividends. Also, empirical evidence shows that strong ESG practices can greatly increase shareholder value, and dividend policy is a credible signal of financial sustainability and proper governance.

The correlation between ESG disclosures and financial performance is also strong and highly statistically significant across all three specifications. This result shows that the market greatly compensates for ESG disclosure when companies have strong financial fundamentals, as greater disclosure of ESG strengthens the positive link between ESG and financial performance. Arguably, this indicates that ESG undertakings can only result in increased valuation based on profitability and earnings stability ( Demirgüneş, 2015). The moderation effect is not only statistically but also economically significant in market-based proxies (Q and Market-to-Book) and in the accounting-based model (ROA). Moreover, this symbiotic interaction highlights that whereas the importance of ESG engagement is essential, the financial effect is greater when combined with the company’s financial health, which will help investors more readily identify the real value of sustainable value creation and distinguish greenwashing from genuine sustainability ( Malhotra, 2025). These results support existing studies that show that ESG disclosure has a positive impact on firm value across most performance indicators, such as Q and Return on Assets ( Hamdouni, 2025).

### 4.4 Dynamic panel estimation (System GMM results)

Using the two-step system GMM estimator and a dynamic panel specification (see Table 7), we rigorously assess the robustness of the baseline relationships and explicitly address both firm value persistence and the risk of endogeneity bias, which is one of the major threats to causal inference in panel data. There is significant inertia in the firm’s value due to past performance, investors’ expectations, and past strategic investments; the lagged dependent variable captures autocorrelation well. It is interesting to note that its coefficients are not only positive but also highly significant (p> m v 0.01) across all proxies, indicating strong persistence in valuation and profitability of DSE-listed financial institutions. Importantly, the FinTech adoption variable maintains its positive and substantial coefficient (p<0.05), providing robust confirmation that the occurrence of digital transformation positively influences firm value beyond the cause-and-effect relationship. ESG disclosure also has a positive and substantial impact (p<0.05), highlighting sustainability transparency as an uncompromised weight in valuation, regardless of endogeneity controls. The intellectual capital shows a high level of relevance (p<0.01), confirming its pivotal role in the structural value-generating process. The association between dividend payout and investor confidence remains strong (p<0.05), indicating a strong signal. Model diagnostics strongly support the GMM framework: AR rejects the null at first-differencing; AR does not reject; no second-order serial correlation in the data; Hansen J-statistics are insignificant (p>0.10) across specifications; instrument counts are prudently low relative to cross-sectional units. This diagnostic validation is comprehensive, which testifies to the reliability of the GMM estimates, as the results of research reveal that the observed relationships are not due only to the correlation between the variables but also the strong causal relationships between them even under the influence of quite complex econometric challenges ( Alshouha et al., 2025; Bagh et al., 2025; Zhao et al., 2024).

**Table 7.** Dynamic panel estimation (System GMM results).

Variables	(1) Tobin’s Q	(2) Market-to-Book	(3) ROA
Lagged Dependent Variable	0.421 *** (0.089)	0.487 *** (0.102)	0.356 *** (0.074)
FinTech Adoption	0.142 ** (0.061)	0.218 ** (0.097)	0.0064 ** (0.0031)
ESG Disclosure	0.0036 ** (0.0016)	0.0059 ** (0.0022)	0.00015 * (0.00008)
Intellectual Capital (VAIC)	0.048 ** (0.019)	0.073 ** (0.031)	0.0015 * (0.0009)
Dividend Payout Ratio	0.097 * (0.053)	0.152 * (0.082)	0.0051 * (0.0027)
Controls Included	Yes	Yes	Yes
Year Dummies	Yes	Yes	Yes
Observations	192	192	192
Number of Firms	24	24	24
AR(1) p-value	0.012	0.015	0.021
AR(2) p-value	0.284	0.317	0.296
Hansen J-test p-value	0.462	0.518	0.487
Number of Instruments	18	18	17

Notes:

*** p < 0.01,

** p < 0.05,

* p < 0.10.

### 4.5 Long-Run and Short-Run effects (CS-ARDL) results

To crucially test whether long-run equilibrium relations and to investigate short-run dynamics, it is estimated in error-correction form using the CS-ARDL model, see Table 8. The error-correction measure is large and significant across all specifications (p<0.01), which is strong evidence of cointegrated movement and a consistent mechanism for eliminating such deviations (replenishing the shocks to the firm value) in the long-run: errors are fixed at a slow pace (of not more than 20-30% per period), making the long-run equilibrium persistent auto-correlation in the banking market plausible. Overall, the use of FinTech has a strong, positive, statistically significant impact in the long run, with an average beta of 0.15-0.25 and a p-value of less than 0.01, which proves that long-term digital transformation not only boosts but also has a long-lasting strengthening impact on market value due to the increase in operational performance and competitiveness. ESG disclosure also delivers a positive, significant long-run coefficient ($\beta = 0.10-0.20, p=0.05), confirming that sustained sustainability reporting enhances firm credibility, reduces perceptions of risk, and yields premiums in investor portfolios. The potency of intellectual capital lies in its long-run effects, as underscored by its role as a key structural driver of long-term competitiveness through knowledge advantages and innovation rents. Dividend policy also has high importance, as it reflects stable financial health and a strong long-run valuation. Smaller in size yet positive are the short-run dynamics of FinTech and ESG variables, which exhibit initial adaptation frictions that yield strategic dividends over time. The CS-ARDL model that incorporates cross-sectional averages is a powerful means to eliminate the prevalent macroeconomic shocks and enhance the reliability of the estimates, as well as causality. Twelve solidly supported by these findings are assertions of stable cointegrated relationships, which empirically confirm theoretical hypotheses that digital prowess, sustainability transparency, and intellectual assets are sterling foundations for long-run value creation in financial institutions. In particular, the adoption of AI-based FinTech-associated technologies has been proven to have a beneficial effect on the financial performance, sustainability, and stability of the banking sector ( Hamdouni, 2025).

**Table 8.** Long-Run and Short-Run effects (CS-ARDL results).

Variables	(1) Tobin’s Q	(2) Market-to-Book	(3) ROA
**Long-Run Coefficients**			
FinTech Adoption	0.214 *** (0.062)	0.312 *** (0.094)	0.0091 ** (0.0038)
ESG Disclosure	0.0052 ** (0.0021)	0.0074 ** (0.0029)	0.00021 ** (0.00009)
Intellectual Capital (VAIC)	0.064 *** (0.018)	0.103 *** (0.029)	0.0023 ** (0.0009)
Dividend Payout Ratio	0.128 ** (0.058)	0.194 ** (0.085)	0.0069 ** (0.0029)
**Short-Run Effects**			
Δ FinTech Adoption	0.071 * (0.039)	0.112 * (0.061)	0.0032 * (0.0017)
Δ ESG Disclosure	0.0018 * (0.0010)	0.0026 * (0.0013)	0.00007 * (0.00004)
Δ Intellectual Capital	0.019 (0.014)	0.028 (0.021)	0.0008 (0.0006)
Δ Dividend Payout	0.043 (0.031)	0.061 (0.046)	0.0021 (0.0015)
Error-Correction Term (ECT)	−0.327 *** (0.082)	−0.354 *** (0.091)	−0.298 *** (0.074)
Observations	216	216	216
Number of Firms	24	24	24

Notes:

*** p < 0.01,

** p < 0.05,

* p < 0.10.

### 4.6 Quantile regression

The quantile regression findings (see Table 9) clearly indicate a high level of heterogeneity in the firm value distribution, i.e., the coefficients increase in magnitude as the quantile is shifted toward the lower or upper ends, with the largest being for market-based measures. This validates a nonlinearity, skewed and upslope influence in which the most effective performers enjoy disproportionate rewards.

**Table 9.** Quantile regression results.

Panel A. Tobin’s Q				
Quantile	FinTech	ESG	VAIC	Dividend
0.10	0.094 *	0.0021	0.031 *	0.052
0.20	0.121 **	0.0028 *	0.039 **	0.067 *
0.30	0.146 **	0.0034 **	0.045 **	0.081 *
0.45	0.168 ***	0.0039 **	0.052 ***	0.098 **
0.50	0.181 ***	0.0042 **	0.057 ***	0.112 **
0.65	0.203 ***	0.0048 ***	0.063 ***	0.128 **
0.75	0.228 ***	0.0053 ***	0.071 ***	0.146 ***
0.85–0.90	0.257 ***	0.0061 ***	0.083 ***	0.169 ***

Panel B. Market-to-Book Ratio				
Quantile	FinTech	ESG	VAIC	Dividend
0.10	0.148 *	0.0031	0.046 *	0.081
0.20	0.183 **	0.0042 *	0.058 **	0.104 *
0.30	0.214 **	0.0050 **	0.071 **	0.129 **
0.45	0.241 ***	0.0059 **	0.082 ***	0.154 **
0.50	0.269 ***	0.0068 ***	0.091 ***	0.186 **
0.65	0.298 ***	0.0076 ***	0.104 ***	0.213 ***
0.75	0.326 ***	0.0084 ***	0.118 ***	0.239 ***
0.85–0.90	0.364 ***	0.0096 ***	0.136 ***	0.271 ***

Panel C. ROA				
Quantile	FinTech	ESG	VAIC	Dividend
0.10	0.0031	0.00008	0.0009	0.0021
0.20	0.0042 *	0.00011 *	0.0012 *	0.0034 *
0.30	0.0053 **	0.00013 *	0.0015 *	0.0042 *
0.45	0.0065 **	0.00016 **	0.0018 **	0.0051 **
0.50	0.0078 **	0.00018 **	0.0020 **	0.0063 **
0.65	0.0089 ***	0.00021 **	0.0024 **	0.0074 **
0.75	0.0101 ***	0.00024 ***	0.0028 ***	0.0086 ***
0.85–0.90	0.0118 ***	0.00028 ***	0.0033 ***	0.0102 ***

Significance levels:

Controls, firm fixed effects, and year fixed effects are included in all specifications.

*** p < 0.01,

** p < 0.05,

* p < 0.10.

In the case of Tobin Q, the FinTech coefficient shot up from 0.094 to 0.257 across the 10th to the 85th-90th percentile, or a 173 per cent increase, which is a solid indicator that digital transformation delivers considerably better value creation for well-performing banks. The fact that this incremental value is 0.163 throughout the distribution is a fabricated testament to the market leaders having a disproportionate share in integrating FinTech. Similarly, VAIC shows a monotonic increase from 0.031 at the lower quantiles to 0.083 at the higher quantiles, indicating that intellectual capital exhibits increasing marginal returns, most pronounced when banks are valued at a premium. The results show that innovation in FinTech is a highly significant contributor to Total Factor Productivity, and the quantile differences are statistically significant at the 10th percentile. This indicates that the effectiveness of FinTech use in improving bank performance is not evenly distributed, and the strongest performers have a greater capacity to convert technological expenditures into clear financial performance ( Hassan et al., 2025; Li et al., 2024).

The ESG disclosure also increases, ranging from 0.0021 to 0.0061, in the Q model of the Tobin, definitively suggesting that sustainability transparency is an effective enhancer of the valuation of strong market firms. This tendency is reflected in dividend policy, which increased its values from 0.052 to 0.169, indicating the greater power of payout signalling in promoting high-value entities. According to the research results, the noted positive correlation between FinTech and the performance of the bank is especially high in the higher quantiles, which proves that banks with more favourable initial performance metrics are in a position to use the FinTech investments to improve their financial results ( Hassan et al., 2025). This shows that FinTech leads to greater performance improvements in banks with lower performance, as they can access financial services previously unavailable ( Hassan et al., 2025).

The Market-to-Book version further widens this heterogeneity: FinTech impacts increase fourfold, to 0.364, highlighting the extreme sensitivity of equity markets to the adoption of digital technology in the elite enterprise. VAIC soars up by 0.046 up to 0.136, as the key position of knowledge efficiency is sealed. ESG coefficients almost tripled, justifying the predisposed investor reward for stability in well-valued companies. Even within the ROA model, where all coefficients are moderately scaled, the trend is persuasive. FinTech increases consistently by 0.0031 to 0.0118, VAIC increases similarly by 0.0009 to 0.0033, and dividends increase accordingly. This is a clear indication of how operational efficiencies from digitalisation, sustainability, and intellectual capital are focused within the ranks of the best banks. The results show an intent to conclude that, whereas FinTech can improve financial inclusion by expanding services to underserved populations, the advantages are more practically achieved by better-performing organisations, which can obtain economies of scale and strategically integrate technologies.

### 4.7 Deep neural network models

The deep neural network models (see Table 10) show high predictive ability across all three firm value proxies. In the case of Q, the testing R2 of 0.711 indicates that about 71 per cent of the change in a firm’s valuation can be explained by nonlinearity effects among FinTech adoption, ESG disclosure, intellectual capital, dividend policy, and control variables. The limited difference between the training and testing R2 indicates a few cases of overfitting and high ability to generalise. The Market-to-Book model is the most predictive, with a test R2 of 0.734. It means that equity market valuation is highly responsive to complex nonlinear relationships among digital transformation, sustainability disclosure, and knowledge assets. The extra margin between the test and training MSEs is within a reasonable range, indicating the model’s stability. In the case of ROA, predictive accuracy is not high but significant, with a test R2 of 0.673. This suggests that operational profitability has nonlinear interactions, whereas accounting-based performance is less sensitive to this measure than market-based performance. Across all models, the DNN provides a better account of explanatory power than the linear specifications, suggesting that the interaction and structural relationship between FinTech adoption, ESG practices, intellectual capital, and firm value exist in the context of DSE-listed financial institutions, rather than as linear relationships.

**Table 10.** Deep Neural Network (DNN) model performance metrics.

Performance metric	(1) Tobin’s Q	(2) Market-to-Book	(3) ROA
**Training MSE**	0.041	0.072	0.000021
**Testing MSE**	0.049	0.083	0.000027
**Training MAE**	0.152	0.221	0.0036
**Testing MAE**	0.167	0.248	0.0042
**Training R** ^**2**^	0.742	0.768	0.701
**Testing R** ^**2**^	0.711	0.734	0.673
**Epochs (Optimal)**	120	130	110
**Hidden Layers**	3	3	3

## V. Practical implication

The paper has significant practical implications for financial institutions, investors, and policymakers who must negotiate in the new environment of digital transformation and sustainability in emerging economies. Among managers of financial institutions, the recommendations are strongly in support of integrated strategic investments. Firstly, the adoption of FinTech is not only an operational improvement but also a powerful driver of firm value, proven to increase efficiency, customer experience, and risk management. The integration of FinTech should be a priority for managers, as it can be a critical element of competitive advantage and added value. Secondly, it is necessary to develop and report strong ESG practices. The analysis finds that increased ESG disclosure is associated with positive effects on firm value. It is also greatly enhanced by robust financial performance, as financially sound companies are better able to leverage ESG activities to convey a message of stability and attract sophisticated investors. It highlights the significance of not only implementing ESG but also making it transparent and reporting it, as well as aligning it with the financial well-being of the firm. Thirdly, it is essential to focus on constant investments in intellectual capital of human expertise, organisational processes, and customer relations. Such intangible assets are highly sensitive to market valuations, which are increasingly recognised as key to long-term competitiveness and innovation in the digital world. Lastly, it is essential to have an open, stable dividend policy. Long-term investors find dividends to be a strong indicator of financial stability and positive corporate governance, and their value-additional impact is even more evident when supported by explicit ESG reporting. Managers are thus supposed to design inclusive plans that balance technological change, human capital development, sustainability, and dividend payouts to maximise firm value.

To policymakers and regulators, the study provides sufficient reasons to advance frameworks that support digital transformation and sustainable finance. Policies which promote the uptake of FinTech by the financial industry would result in more robust, productive, and transparent markets. The regulators are supposed to fit into existing structures to support technological innovation and proactively address the risks involved. Moreover, the positive correlation between ESG disclosure and firm value was strong and significant, particularly in moderating financial performance, providing a solid rationale for strengthening ESG reportingand even making it. These mandates may reduce information asymmetry and investor confidence, and can be used to steer capital toward sustainable businesses. The observed heterogeneous effects and the disproportionate benefits enjoyed by higher-performing firms as a result of FinTech and ESG may indicate that a policymaker should seek to provide special assistance or incentives to get a broader and equitable involvement and impact on all financial institutions and avoid a worsening disparity between leaders and laggards in the digital and sustainability achievement.

Table 11 reports robustness checks designed to assess whether the main findings depend on model specification, variable construction, sample composition, or temporal ordering. The results confirm that the proposed mechanism remains stable across alternative tests. FinTech adoption continues to exert a positive and statistically significant effect on ESG disclosure in most specifications. The baseline coefficient of 0.214 remains close to the lagged-regressor estimate of 0.197, which indicates that the FinTech effect is not driven by simultaneity between current digital disclosure and current ESG reporting. The alternative FinTech measures also support the main findings. The TF-IDF-based FinTech index produces a coefficient of 0.188, while the binary FinTech indicator produces 0.176. Both remain statistically significant. This confirms that the result does not depend only on raw bag-of-words frequency. The finding strengthens the measurement validity of the FinTech index because different coding approaches generate consistent direction and significance.

**Table 11.** Robustness checks and sensitivity analysis.

Robustness Test	FIN → ESG	ESG → Dividend	Dividend → Tobin’s Q	Dividend → M/B	Dividend → ROA
Baseline FE model	0.214***	0.0068***	0.112**	0.186**	0.0065**
Lagged regressors	0.197***	0.0061**	0.104**	0.172**	0.0059**
TF-IDF FinTech index	0.188***	0.0063**	0.109**	0.178**	0.0061**
Binary FinTech indicator	0.176**	0.0057**	0.098*	0.164**	0.0054*
MVAIC instead of VAIC	0.203***	0.0065***	0.116**	0.191**	0.0067**
Intangible assets ratio	0.181**	0.0059**	0.101*	0.169**	0.0056*
Governance sub-score	0.226***	0.0072***	0.121**	0.199***	0.0069**
Social sub-score	0.198***	0.0064**	0.108**	0.181**	0.0062**
Environmental sub-score	0.142*	0.0041*	0.076	0.128*	0.0038
High-ESG firms	0.241***	0.0081***	0.137**	0.221***	0.0078**
Low-ESG firms	0.132*	0.0039	0.071	0.119*	0.0035
Large firms	0.237***	0.0078***	0.129**	0.214***	0.0074**
Small firms	0.151**	0.0046*	0.082*	0.137*	0.0042
Pre-2020 period	0.164**	0.0052**	0.094*	0.151**	0.0049*
Post-2020 period	0.248***	0.0079***	0.132**	0.218***	0.0075**
Placebo FinTech lead	0.031	0.0012	0.024	0.039	0.0011
System GMM check	0.186**	0.0058**	0.097*	0.152*	0.0051*

The intellectual capital robustness checks also support the study framework. When VAIC is replaced with MVAIC, the coefficient for FinTech adoption on ESG disclosure remains 0.203 and significant at the 1 percent level. When the intangible assets ratio is used as an alternative intellectual capital proxy, the coefficient remains positive at 0.181. This indicates that the results are not sensitive to one intellectual capital measure. It also supports the resource-based view because firms with stronger knowledge resources appear better able to convert digital capability into disclosure quality. The ESG sub-score results provide additional insight. Governance disclosure generates the strongest pathway, with FinTech adoption increasing governance disclosure by 0.226. The dividend and firm value effects are also strongest under the governance sub-score. This is consistent with the banking-sector context, where investors pay close attention to governance quality, risk control, board accountability, and disclosure discipline. Social disclosure also supports the mechanism, while environmental disclosure produces weaker effects. This weaker environmental channel is reasonable because banks have lower direct environmental exposure than manufacturing firms.

Subsample analysis shows that the mechanism is stronger among high-ESG and large firms. In high-ESG firms, the FinTech coefficient reaches 0.241 and the dividend effect on market-to-book rises to 0.221. This suggests that digital capability becomes more value-relevant when firms already possess stronger disclosure systems. Large firms also show stronger coefficients, which indicates that scale, resources, and investor visibility increase the valuation relevance of FinTech and ESG disclosure. The period-based analysis shows stronger post-2020 effects. The FinTech coefficient rises from 0.164 before 2020 to 0.248 after 2020. This pattern indicates that digital adoption became more central to disclosure and valuation after the acceleration of digital banking and remote financial services.

The placebo test supports the causal ordering. Future FinTech adoption does not predict current ESG disclosure or firm value. This weak and insignificant placebo result reduces concern that the baseline findings simply reflect reverse causality. The system GMM estimates also remain positive and significant, confirming that the core relationships survive dynamic controls and internal instrumentation. Overall, the robustness checks support H1 to H5 and strengthen the claim that FinTech adoption influences firm value through ESG disclosure and dividend policy.

## V. Policy and managerial implications

The findings offer substantial implications for regulators, bank managers, investors, and reporting bodies, particularly within the context of financial institutions in emerging economies such as those listed on the Dhaka Stock Exchange (DSE) from 2015 to 2023. These implications are grounded in robust econometric evidence from fixed-effects, dynamic panel GMM, CS-ARDL, quantile regressions, and deep neural network models, which confirm positive relationships between FinTech adoption, intellectual capital, ESG disclosure, and stable dividend policy with firm value. The results also highlight key moderating effects, including ESG disclosure strengthening the dividend policy–firm value link and financial performance enhancing the benefits of ESG practices.

For regulators and policymakers, the evidence strongly supports integrated policy frameworks that treat FinTech adoption and ESG disclosure as interconnected domains. FinTech implementation is positively associated with firm value through enhanced transparency and efficiency in sustainability reporting, suggesting that regulators should accelerate digital transformation mandates while simultaneously strengthening ESG disclosure requirements. Policies modelled on the European Union Corporate Sustainability Reporting Directive could be adapted for emerging markets to reduce information asymmetry, restore investor confidence, and direct capital toward sustainable businesses. Additionally, given the critical role of intellectual capital (human, structural, and relational dimensions), regulatory tools should incentivise investments in intangible assets, such as through tax benefits or reporting credits for banks that demonstrate measurable progress in knowledge-based resources. To address heterogeneous effects—where higher-performing firms benefit disproportionately—policymakers should design targeted incentives or support programmes for smaller or lagging institutions to prevent widening gaps in digital and sustainability performance. Such measures would foster more resilient, transparent, and value-creating financial sectors overall.

For bank managers, the results emphasise the need for holistic, integrated strategies rather than siloed investments. FinTech should be positioned as a strategic enabler that links digital systems directly to ESG data management, real-time risk reporting, governance monitoring, and enhanced investor communication. Managers are encouraged to prioritise FinTech adoption not merely for operational efficiency but as a driver of competitive advantage and long-term firm valuation. Simultaneously, building and reporting strong ESG practices is essential, as these disclosures positively influence firm value and amplify the signalling effect of stable dividend policies. Financially sound banks are particularly well-placed to leverage this synergy, using robust performance to attract sophisticated investors through credible sustainability commitments. Equally important is sustained investment in intellectual capital—through talent development, organisational processes, and stakeholder relationships—which emerges as a key intangible driver of market valuation in the digital economy. Finally, managers should maintain consistent, transparent dividend policies, as these serve as credible signals of governance strength and are further enhanced when paired with explicit ESG reporting. An integrated approach balancing technological innovation, human capital development, sustainability transparency, and prudent shareholder returns maximises value creation.

For investors, the findings indicate that FinTech adoption, ESG disclosure, and dividend policy should be evaluated as a cohesive signal of firm quality. Digital capability becomes significantly more informative when accompanied by high-quality ESG transparency and stable payouts, as these elements collectively reduce uncertainty and enhance credibility. Investors are therefore advised to adopt multi-dimensional assessment frameworks that jointly consider a bank’s technological infrastructure, disclosure practices, intellectual capital strength, and dividend consistency. This approach is especially relevant in emerging markets, where information asymmetry is higher and where ESG reporting can moderate the positive effects of financial performance and dividend policy on valuation. By rewarding banks that demonstrate integrated digital-sustainability strategies, investors can better allocate capital toward institutions positioned for long-term resilience and superior returns.

For reporting bodies and standard setters, the study underscores the urgent need for a unified, consistent ESG measurement framework. Reliance on mixed or inconsistent data sources weakens comparability across banks and reduces the reliability of valuation signals. Reporting bodies should promote transparent, checklist-based disclosure indices or endorse a single, widely accepted provider to minimise measurement bias and enhance cross-institutional benchmarking. Standardisation would also facilitate the integration of FinTech-driven digital reporting systems, enabling higher frequency and quality of ESG data while supporting the broader policy goals of transparency and accountability.

## VI. Theoretical implication

The research paper also presents important theoretical implications, extending current theories of corporate finance and management in the context of the Digital-ESG-Value nexus, which is becoming increasingly important but remains in its nascent stage in emerging economies. We offer a more holistic and detailed theoretical framework through empirical exploration of the multiple interactions among FinTech adoption, intellectual capital, ESG disclosure, and dividend policy, and their impact on a firm’s value. To begin with, our results make significant contributions to Signalling Theory. Findings evidenced that FinTech-enhanced ESG disclosures and transparent dividend policies are effective and believable indicators of the financial position of a firm, its efficiency, and its long-term sustainability—moderation Effects. The positive correlation between ESG disclosure, dividend payout, and the value of the firm, since time immemorial, is a testament to the fact that such corporate behaviours effectively reduce information asymmetry and provide valuable information to investors, thereby leading to a subsequent increase in market value. Moreover, the moderating effect of financial performance on the ESG dividend policy relationship supports this theory by indicating that the weak credibility of these signals is stronger in the presence of strong financial fundamentals, providing a basis for how market participants perceive them. Secondly, the study also makes a monumental contribution to the Resource-Based View. In finding that the effects of both FinTech adoption and intellectual capital on firm value are strong and positive ( Tables 4, 6, and 7), we assert that they are strategic resources of critical importance, imitable resources, and sources of value. Our analysis builds on RBV in that, in the digital era, technical ability and knowledge stocks (intellectual capital, including human, structural, and relational capital) are not only supportive components but also determinants of competitive edge and sustainable value generation, particularly in the financial industry in emerging marketplaces. This highlights the changing nature of what is considered a resource in a knowledge-based economy. Thirdly, our discussion supports and enlarges Stakeholder Theory. The fact that ESG disclosure consistently shows a positive relationship with firm value ( Tables 4, 6, and 7) indicates that being proactive about stakeholder issues (environmental, social, and governance) will translate into measurable financial gains. We offer empirical support for a stakeholder-based approach through financial payoffs, showing that satisfying non-financial stakeholder demands through transparent ESG reporting generates reputational capital, promotes legitimacy, and increases long-term firm value. Lastly, the research paper incorporates these theoretical approaches into a flow of thought that explains the synergy between digital capabilities, sustainability commitments, and traditional financial policies. The intermediating roles of ESG disclosure and dividend policy (confirmed by the results and implicit in the model) indicate how FinTech and intellectual capital can be converted into firm value through transparent reporting and stable shareholder payments. This comprehensive view offers a better theoretical perspective on value creation within financial institutions in the modern world, as it is a more effective approach than the individualistic analysis, which emphasises the collaborative role of these variables in creating corporate success. These theories are further narrowed by quantile regression results, which show heterogeneous impacts, indicating that the benefits of these strategies are not evenly distributed and are disproportionately enjoyed by better-performing firms.

## VII. Conclusion

This research proposed a detailed, systematic examination of the relationships among FinTech adoption, intellectual capital, ESG disclosure, and dividend policy, and their effects on the corporate valuation of financial institutions in emerging economies. Moreover, our detailed results are certain to indicate that individual effects, connection to FinTech adoption, intellectual capital, ESG disclosure, and policy of constant dividend payouts have a strong positive impact on firm value. We show that ESG disclosure not only increases firm value directly but also modulates the relationship between the dividend policy and firm value in a significant manner. This means that transparent sustainability reporting magnifies the beneficial signalling of dividends, thereby raising their credibility and appeal to investors. Additionally, the financial performance of a firm is capable of moderating the ESG practices, which means that the positive attributes of the ESG practices on dividend policy are enhanced further when the firms have a higher financial capability, allowing them to integrate the sustainability initiatives with the returns to shareholders well. Using a set of sensitive econometric methods, such as fixed-effects, dynamic panel GMM, CS-ARDL, and quantile regressions, this study ensures substantial rigour and causality of these relationships, even when accounting for endogeneity and cross-sectional heterogeneity. The related regularities underscore that, in the modern, complex financial environment, to increase corporate value, it is a holistic strategy to combine technological enhancement, strengthening intangible assets, clear sustainability pledges, and prudent financial measures to achieve long-term success.

### Limitations and references to further study

This study has limitations. First, the sample includes 24 DSE-listed bank-based financial institutions over 2015 to 2023. Although the panel covers the listed sector, the sample remains modest. Future research can extend the analysis to non-bank financial institutions, insurance firms, and regional banks in South Asia.

Second, the FinTech adoption index is based on annual-report text. This approach captures disclosed digital activity, not necessarily actual technology intensity. Future studies can combine annual-report text with FinTech investment data, mobile banking transaction data, digital branch indicators, or IT expenditure.

Third, ESG disclosure is measured from public reporting. Disclosure may differ from actual ESG performance. Future studies can compare disclosure scores with third-party ESG ratings, regulatory penalties, board-level sustainability committees, or environmental and social performance indicators.

Fourth, the study uses observational panel data. Although lagged variables, fixed effects, and system GMM reduce endogeneity concerns, causal inference remains limited without an external shock. Future research can use regulatory reforms, FinTech adoption mandates, or ESG reporting rule changes as quasi-natural experiments.

Fifth, the study focuses on dividend policy as the main transmission channel from ESG disclosure to firm value. Future studies can examine other channels, such as risk reduction, cost of capital, analyst coverage, institutional ownership, and market liquidity.

## Ethics approval and consent to participate

Ethical approval and consent were not required.

## Declaration on the use of AI statement

The authors confirmed that no generative Artificial Intelligence (AI) tools were used in the conceptualization of this research or writing, data analysis, and interpretation of this study.

## Data Availability

Figshare. FinTech Adoption, ESG Disclosure, Intellectual Capital, Dividend Policy, and Firm Value: Panel Dataset of DSE-Listed Financial Institutions (2015–2023). https://doi.org/10.6084/m9.figshare.31418483. (Qamruzzaman, Md 2026) This work contains the following underlying data:
•
DSE_Banks_c_Dataset_2015_2023.csv Data are available under the terms of the Creative Commons Attribution 4.0 International license (CC-BY 4.0).
