# FinTech Adoption and ESG Disclosure in Corporate Valuation: Intellectual Capital and Financial Performance Effects on Dividend Policy and Firm Value

**DOI:** 10.12688/f1000research.178277.3

**Published:** 2026-07-30

**Authors:** Md Qamruzzaman, Abdulrahman Alomair, Mohammed Alomair

**Affiliations:** 1 School of Business and Economics, United international University, Dhaka, 1212, Bangladesh; 2 Accounting Department, , Business School, King Faisal University, Al Ahsa, 31982, Saudi Arabia

**Keywords:** FinTech Adoption, ESG Disclosure, Intellectual Capital, Dividend Policy, Corporate Valuation

## Abstract

**Background:**

This study examines how FinTech adoption and intellectual capital shape ESG disclosure, and how ESG disclosure and dividend policy are associated with firm value in DSE-listed financial institutions. The framework focuses on a sequential Digital—ESG—Value mechanism rather than treating all constructs as parallel determinants.

**Method:**

Using an unbalanced panel of DSE-listed bank-based financial institutions from 2015 to 2023, the study estimates firm and year fixed-effects models as the primary explanatory specification. System GMM is used to assess dynamic persistence and potential endogeneity, while quantile regression evaluates distributional heterogeneity. CS-ARDL is retained as a supplementary long-run sensitivity analysis, and a deep neural network is used only to assess out-of-sample prediction and nonlinear feature importance.

**Findings:**

FinTech adoption and intellectual capital are positively associated with ESG disclosure. ESG disclosure is positively associated with dividend policy and firm value, while dividend policy is also positively associated with firm value. Financial performance conditions the ESG–dividend relationship. These estimates indicate robust associations but do not establish experimental causality. The predictive analysis confirms that FinTech, ESG disclosure, intellectual capital, and dividend policy contain information relevant to firm-value prediction.

**Conclusion:**

The findings extend signalling theory, the resource-based view, and stakeholder theory by showing how digital capability and knowledge resources can support credible sustainability disclosure and market signalling. The results remain specific to listed Bangladeshi financial institutions and should be interpreted within that institutional setting.

## I. Introduction

This paradigm shift requires a re-evaluation of traditional theories of corporate finance, which have conventionally predicted that physical capital is a more accurate predictor of the intrinsic determinants of firm valuation in present-day markets. It can be seen in the platform economy, where Uber is one such business whose value is almost entirely derived from customer and driver networks rather than physical resources ( Beaumont, 2019). The increasing visibility of digital transformation, artificial intelligence, and big data has only enhanced the strategic importance of intangible assets such as data, brand equity, and intellectual property as the tangibility of assets globally declines ( Vengesai, 2023). The example of this dynamic is the increasing difference between the markets and the book valuation, which is caused by the growing significance of intangible capital investments in driving corporate innovation and long-term sustainability; to be more precise, the book equity has recorded systematic decrease in the U.S. firms as the importance of intangible capital investments in the form of knowledge and human capital investment increases ( Luo, 2020). Those phenomena challenge established valuation models, i.e., the ones propounded by Fama and French, which are largely based on physical book values and therefore factor into the recent underperformance of the value factor, often causing the misrepresentation of the actual value of data-based companies like Facebook and Twitter and any other entities with large amounts of intangible capital ( Eisfeldt et al., 2020; Shi, 2024). As such, it is urgently required to understand the economic implications of these intangible assets, specifically, their impact on the returns of equity, the cost of capital, and the valuation of the firm overall as an investor ( Dong and Doukas, 2025).

The use of FinTech through the focus on intangible assets in the spectrum of data and artificial intelligence ( Vengesai, 2023), and the introduction of emerging technologies, including AI, blockchain, and big-data analytics has had a significant effect on reconfiguring the traditional financial models and, in turn, spawned innovation and efficient work of the capital market ( Lăzăroiu et al., 2023). This integrative technological paradigm enhances the effectiveness, transparency, and cost-efficiency of financial transactions through blockchain-based data provisioning and AI-intuited analytics, thereby transforming the competitive landscape of both established financial institutions and new market entrants ( Beaumont, 2019; Hasan et al., 2024). The implication of FinTech goes beyond operational improvements, whereby corporate investment direction and ultimately the value of the firm have been impacted through more secure, efficient, and cost-reduced financial transactions. In addition, strong sustainability reporting in FinTech companies correlates with high valuations and better market performance ( Yuan et al., 2024). This transformational ability refers to shifting the paradigm from products to clients and requires a flexible, technology-driven solution to remain relevant in the market ( Ahangar and Salman, 2020). The implications of its extensive implementation of artificial intelligence in FinTech activities, specifically, are that the value generation changes its form: no longer to traditional revenue-generating models, but to investor confidence instead, and the importance of AI as a transformative force ( Visconti, 2024). The issues posed by these new technological paradigms necessitate reevaluating existing financial and economic paradigms through the prism of sustainability and machine learning, thereby allowing the formulation of new frameworks of analysis ( Berrou, 2021).

This focus on intellectual capital 3 both human expertise, organisational procedures and customer relations points to an even larger shift towards recognising these aspects as key sources of competitive edge, as well as drivers of economic growth in the knowledge economy. Corporate innovation and long-term survival are increasingly dependent on intangible investments in human capital and knowledge ( Luo, 2020). Companies with high levels of intangible assets are more likely to focus on the quality of financial reporting to enhance market transparency, thereby allowing stakeholders to benefit from more accurate price changes ( Dong and Doukas, 2025). This kind of transparency is also supported by FinTech developments that advance the reporting of Environmental, Social, and Governance (ESG) matters and promote financial inclusion by democratising the influence of green investments ( Hasan et al., 2024). The development of FinTech, in particular the use of AI, the Internet of Things, big data analytics, and blockchain, plays a crucial role in advancing green and sustainable financial services and products, as evidenced by the development of consistent climate-risk assessment systems. Simultaneously, strong sustainability metrics in FinTech firms are associated with high valuations and better market results ( Lăzăroiu et al., 2023; Merello et al., 2021). Its nature is driven by the convergence of sustainable finance values and ESG concerns, and it is key to supporting the growth trend of inclusive growth and stability in global financial markets during times of volatility, as evidenced by the ability to reduce risks and enhance the stability of interconnected financial markets ( Arnone and Leogrande, 2024; Mani, 2024).

This urgency highlights why corporations must not only establish financial performance but also strive to impact the environment and society positively, including the aspect of ethical governance, and openly convey these efforts to the stakeholders, which makes them more resilient and appealing to investors ( Arfaoui-Masmoudi and Hazami-Ammar, 2024; Merello et al., 2021). Continuing the ESG transparency and financial inclusion innovations in the FinTech industry ( Hasan et al., 2024), the ubiquitous integration of ESG factors into the investment process represents a vital paradigm shift, making these aspects inseparable from full-fledged investment strategies and decision-making ( Shah, 2024). This evolution highlights the substantial role of enhanced analytics, i.e. AI, big data, and blockchain, in the evaluation and incorporation of ESG performance ( Lăzăroiu et al., 2023; Olanrewaju et al., 2024). Global regulators are subjecting companies to wider and more comprehensive disclosure requirements on sustainability/environmental, social, and labour (ESL) practices; to give one example, the European Union Corporate Sustainability Reporting Directive focuses on ensuring that large and small companies engage in comprehensive reporting on their sustainability practices (environmental, social, and labour) ( Shirai, 2023). This is the regulatory wave, which, along with an increasing interest in sustainable investments among investors, strengthens the inextricable connection between strong ESG performance and long-term financial sustainability, including high valuation, strong market performance, and elevated shareholder returns ( Ahangar and Salman, 2020; Arnone and Leogrande, 2024; Merello et al., 2021).

This difficulty lies in the very nature of the intellectual capital, which is intangible and subjective in nature - an area where there is no consensus on its definition, measurement and an appropriate framework - and is made difficult by the fact that even valuation processes of AI-driven technologies in the financial sector are nascent and non-standardised ( Ammar and Kamaruddin, 2025). The lack of uniform valuation and accounting frameworks for technology and information assets in FinTech adds to this challenge, preventing their consistent placement on balance sheets and undermining the overall reliability of the market ( Bayón and Vega, 2018). Further, artificial intelligence has a significant transformative impact on the value-creation process, altering operational patterns, revenues, and investor trust in organisations and enterprises operating in the FinTech sphere. However, its exact contribution to firm value is still elusive, as it can either be a factor that improves the other intangibles or the result of investments in intellectual capital, either way depending on the presence or absence of various institutional settings such as market efficiency and regulatory preparedness ( Elkmash and Mohamed, 2025; Visconti, 2024). Indeed, in a recent example, innovation has a role of great complexity and subsidence in capital structure; a decline in the intensity of R&D could be an indication of competitive disadvantages or growth constraints, which would subsequently drive negative adjustments in investor valuation ( Kruglov and Shaw, 2024). On the contrary, although intellectual capital has the potential to add value, it is not always a good predictor of firm value due to model constraints that fail to account for mediating variables, such as profitability, and to capture the financial impact of investments on intellectual capital ( Appah et al., 2023). This disparity also extends to the performance reporting of intellectual capital, with most companies not clearly disclosing its impact on firm value in annual or sustainability reports, making it difficult for investors to assess its impact amid an unresolved measurement dilemma ( Kruglov and Shaw, 2024). Also, this effect of evidence is particularly strong in less developed markets, where institutional and structural peculiarities, such as insufficient integration of ESG and methodological flaws (e.g., endogeneity), further moderate these dynamics ( AlQudah et al., 2025). Also, little has been done in the area of optimal balance and synergistic integration of diverse knowledge assets with corporate social responsibility to optimise market value ( Christofi et al., 2024). Finally, the complex interdependence of these intangibles, including strategy, ownership, management, CSR, and external factors, and how they are transformed into sustainable competitive advantage and shareholder returns, is an issue that will require stringent empirical investigation to guide and improve valuation models ( Campo and Calvo, 2025).

The study therefore focuses on one sequential mechanism. FinTech capability and intellectual capital provide the information-processing and organisational resources required for credible ESG disclosure. ESG disclosure then influences dividend policy by reducing information asymmetry and clarifying the firm’s capacity to balance sustainability commitments with shareholder distributions. Dividend policy operates as a market signal through which these internal capabilities and disclosure choices are reflected in firm value. This structure narrows the analysis to the Digital–ESG–Dividend–Value pathway and avoids treating every variable as an independent and simultaneous source of valuation. The study evaluates these relationships in Bangladesh, where rapid financial digitalisation, developing sustainability-reporting practices, concentrated ownership, and bank-centred financing create a useful institutional setting for examining whether digital and intangible capabilities improve disclosure credibility and valuation. The findings are not assumed to generalise automatically to all emerging markets; instead, Bangladesh is treated as an institutionally specific case from which comparative hypotheses can be developed.

RQ1: Are FinTech adoption and intellectual capital positively associated with the quality of ESG disclosure?

RQ2: Is ESG disclosure positively associated with the dividend policy of DSE-listed financial institutions?

RQ3: Does financial performance condition the association between ESG disclosure and dividend policy?

RQ4: Is dividend policy positively associated with firm value after controlling for firm characteristics and common year effects?

The study contributes by testing an integrated but parsimonious sequence linking digital capability, intangible resources, sustainability disclosure, payout policy, and market valuation. Its theoretical contribution is not the introduction of additional constructs, but the specification of distinct roles for the existing constructs. The resource-based view explains why FinTech capability and intellectual capital improve the firm’s capacity to produce, verify, and communicate ESG information. Stakeholder theory explains why firms disclose ESG information in response to demands for accountability. Signalling theory and information-asymmetry arguments explain how credible ESG disclosure and dividend decisions convey information to investors. Agency theory provides a complementary explanation for dividend policy by treating payouts as a mechanism that limits managerial discretion over free cash flow. Together, these perspectives describe a sequential resource–disclosure–signal–value mechanism. The empirical contribution lies in evaluating this mechanism using explanatory panel models, distributional estimates, and a separate predictive analysis while maintaining cautious interpretation of causal claims.

For managers, the framework implies that digital investment and human-capital development should be assessed according to their capacity to improve information quality, disclosure credibility, and disciplined payout decisions. The results do not imply that technology spending automatically raises market value. Valuation benefits are more likely when digital systems are embedded in reporting controls, staff capabilities, governance processes, and investor communication. Managers should therefore connect FinTech investments with verifiable ESG data, internal assurance, and a dividend policy consistent with profitability and capital requirements.

For regulators and policymakers, the study indicates that digitalisation and ESG reporting should be governed as connected disclosure infrastructures. Bangladesh provides a relevant setting because listed financial institutions operate within a bank-centred system in which regulatory guidance, disclosure quality, and investor confidence remain closely linked. Policy measures should prioritise comparable ESG metrics, auditable digital records, and clear guidance for reporting technology adoption. Because the evidence comes from one market and a limited period, the policy implications should be applied cautiously and validated through comparative studies in other emerging economies.

Remaining structure of the article are as follows. Section II deals with theoretical development and literature review, data and methodology explained in Section III, result and discussion available in Section IV, practical implications and theoretical implications reported in Section V and VI, finally conclusion available in Section VII, respectively.

## II. Theoretical underpinning and literature review

### 2.1 Theoretical foundations

*Agency Theory:*

Agency theory explains the dividend-policy stage of the framework. When managers control substantial free cash flow, they may retain resources or invest in projects that do not maximise shareholder value. Dividend payments reduce discretionary cash and expose firms to external capital-market scrutiny when additional financing is required. In this study, agency theory does not independently explain FinTech adoption or ESG disclosure. It complements the signalling mechanism by clarifying why a disciplined payout policy may be valued by investors, particularly when ESG expenditure creates concern about managerial overinvestment or symbolic sustainability spending.

*Signalling Theory:*

Signalling theory provides the central explanation for the disclosure and valuation stages. Managers possess information about earnings quality, liquidity, digital capability, and sustainability commitments that outside investors cannot observe directly. Credible ESG disclosure can reduce this gap when it is supported by verifiable data and consistent operating performance. Dividend payments provide an additional costly signal because they require distributable cash and cannot be sustained indefinitely without financial capacity. The framework therefore predicts that ESG disclosure and dividend policy jointly communicate firm quality, although the strength of the signal depends on financial performance and disclosure credibility.

*Resource-Based View (RBV):*

The resource-based view explains the antecedent stage. FinTech infrastructure, analytics capability, employee expertise, organisational routines, and stakeholder relationships represent intangible resources that can improve data capture, internal control, service innovation, and reporting quality. Intellectual capital and FinTech adoption are therefore modelled as capabilities that enable ESG disclosure rather than as unrelated valuation variables. Their value arises when firms combine these resources in ways that are difficult to replicate and direct them toward credible sustainability reporting and operational decision-making. Rababah, Molavi, and Farhangdoust (2022) similarly show that R&D, marketing, and financial decisions contribute to value creation, while Rababah, Javed, and Malik (2022) emphasise the performance role of social capital and managerial intangible capability.

*Stakeholder Theory:*

Stakeholder theory explains why firms use these resources to produce ESG disclosure. Financial institutions face demands from shareholders, depositors, borrowers, employees, regulators, and communities for information on environmental exposure, social conduct, and governance quality. Disclosure responds to these demands and can preserve legitimacy and access to resources. Within the integrated model, stakeholder pressure determines the relevance of ESG information, while the resource-based view explains the firm’s capacity to produce it and signalling theory explains how investors interpret it.

*Information Asymmetry:*

Information asymmetry links the theoretical stages. Annual reports and sustainability disclosures reduce the difference between managerial and investor information, but only when the underlying data are sufficiently complete and credible. FinTech tools can support traceability, timeliness, and consistency, while intellectual capital determines whether the firm can interpret and govern these systems. Reduced information asymmetry may lower uncertainty and improve investor assessment of dividend sustainability and firm value. This argument supports associations among the constructs; it does not by itself establish causal identification.

*Technology Adoption (Diffusion):*

Technology-adoption theory provides a boundary condition rather than a separate causal pathway. Financial institutions adopt FinTech when expected efficiency, regulatory compliance, customer demand, and competitive benefits exceed implementation costs and risks. Adoption intensity may therefore reflect organisational readiness and external pressure. The study uses this perspective to interpret variation in the FinTech index, while the resource-based view remains the principal theory connecting adoption to ESG disclosure.

### 2.2 Literature review

A. The adoption of FinTech and its implications for ESG Disclosure

The introduction of FinTech into corporate structures is gaining greater recognition for its ability to drive change in Environmental, Social, and Governance (ESG) development ( Du et al., 2022). Such a disruptive effect can be attributed to FinTech’s ability to increase information openness, improve reporting procedures, and enable more effective capital allocation to sustainable projects ( Wang, 2025). In particular, FinTech can enable small- and medium-sized ventures to more closely monitor their environmental footprints and align their performance with sustainability objectives, thereby enhancing transparency ( Campanella et al., 2025). Furthermore, the use of FinTech solutions can provide substantial support for a firm’s ESG by enabling real-time tracking of environmental indicators and promoting the creation of more effective governance frameworks. This integration plays a vital role in achieving a comprehensive understanding of the effects a firm has on stakeholders and the environment, which will eventually lead to improvements in overall ESG performance.

Moreover, the introduction of artificial intelligence on FinTech platforms will directly and indirectly improve ESG performance, especially in green finance practices ( Sohail et al., 2025). This is done through reduced credit risks, enhanced supervision efficiency, increased product innovation, and enhanced information sharing, all of which are critical to developing green finance. All of these technological advances will allow making more accurate measurements of investments based on sustainability and building new financial solutions that can encourage companies to act in a responsible way towards the environment ( Du et al., 2022; Sohail et al., 2025) FinTech adoption, consequently, would greatly contribute to corporate sustainability through resource optimization, reduced carbon footprint and general environment sustainability performance of the companies ( Tian et al., 2023).

Path 2: How Intellectual Capital affects ESG Disclosure

Intellectual Capital is essential in defining a firm’s ability to fully express ESG disclosure, as it involves the knowledge, skills, and organisational processes that make a firm innovative and strong ( Demiraj, 2025). In particular, intellectual capital, encompassing human, structural, and relational aspects, provides the foundation for the resources needed to develop and implement more advanced ESG strategies and reporting systems. Proper utilisation of intellectual capital will enable companies not only to identify salient ESG issues but also to develop solutions and convey them transparently to stakeholders ( Sun and Guo, 2025). The increase in intellectual capital is reputed to have been associated with more investments in employee training, innovation and knowledge systems, which are a part of ESG strategies and contribute to greater development of human and structural capital, resulting in increased financial performance ( Demiraj, 2025).

Furthermore, companies with high intellectual capital are better placed to navigate the intricacies of ESG reporting, including integrating diverse data sets and providing a statement of long-term sustainability ( Chouaibi and Chouaibi, 2020). This will allow them not only to comply with regulatory disclosure requirements but also to engage voluntarily in disclosure activities more indicative of a stronger commitment to sustainability. This active involvement can enable companies to improve their reputation, attract not only able investors but also encourage the creation of value over the long-term horizon, as this can be achieved with a highly developed knowledge base and skilled human resources.

Hypothesis 2:
*The higher the level of intellectual capital, the more transparent and comprehensive ESG disclosures have a positive effect.*

### 2.3 ESG disclosure and corporate policy

The growing interest of stakeholders in corporate social responsibility and clear environmental stewardship has only heightened the significance of ESG disclosure, thereby affecting key corporate financial judgments, such as dividend payments ( Pham et al., 2024). Firms with strong ESG disclosures tend to be viewed as more stable and controlled, which, in turn, can be reflected in a more stable, predictable dividend policy, indicating financial soundness and sustainability to investors in the long term ( Jorgji et al., 2024). Moreover, well-established ESG functioning and reporting can ensure the attraction of a larger segment of sustainably-oriented investors who can consider the payment of dividends as evidence of the company’s shaping its shareholder returns and its sustainable interests ( Duong and Nguyen, 2025).

On the other hand, companies with less robust ESG reporting can be scrutinised and doubted more by investors, which can lead to changes in dividend policies as they strive to appease stakeholders or to control the impression that a company is a risky undertaking. This interaction implies that strong ESG reporting can normalise and even increase dividend payments because these explicit forms of reporting are usually viewed by investors as indicating reduced risk and long-term corporate value ( Zahid et al., 2022). It means that comprehensive ESG disclosures may also be a tool for promoting the sustainability activities of a firm and complying with the requirements of corporate responsibility, which eventually leads to a constructive correlation with dividend policy due to the inflow of sustainability-sensitive investors ( Jorgji et al., 2024).

Hypothesis 3:
*ESG disclosure has a positive effect on dividend payout, that is, the ways companies use to attract attention to their shareholders’ interests and meet their expectations of corporate accountability.*

B. Intellectual Capital and Dividend Policy

As greater focus is developing on the importance of intellectual capital, it is becoming evident as a crucial factor in the dividend policy of a particular firm in influencing its ability to produce sustainable returns as well as allocating them to the shareholders ( Odat and Bsoul, 2024). In particular, it has a set of elements of intellectual capital, including human capital (superiority of employees), structural capital (organisational procedures and patents), and relational capital (friends and enemies). Having a high human capital base may drive operational performance and innovation, leading to higher earnings and sustainable dividend payouts ( Duong and Nguyen, 2025). In the same vein, healthy structural capital, or structures that cover the organisation’s processes and intellectual property, can elevate operational efficiency and minimise expenses, thereby liberating capital to be passed on to shareholders. Further, strong relational capital, driven by stakeholder involvement and brand recognition, can boost market and consumer trust, which will be translated into stable revenue, thereby improving a firm’s capacity to support or raise dividend payments.

Hypothesis 4:
*Intellectual capital is positively related to a firm’s tendency to make higher and more uniform dividend payouts.*

C. Financial Performance as a Moderator

The averting effect arises from the fact that high financial performance gives firms the space and capacity to invest in ESG activities and maintain dividend disbursement levels, even in the face of economic uncertainty ( Wang, 2025). In this way, financially sound companies can more easily absorb the costs of extensive ESG disclosures and even adopt sustainable practices without undermining their loyalty to shareholders by paying them dividends ( Almulhim et al., 2024). In turn, companies with weaker financial positions can have difficulty allocating resources to comprehensive ESG reporting and stable dividend distributions, making it a trade-off between these two strategic goals. This implies that financial strength can be seen as an important facilitator that enables businesses to address the perceived dilemma between investing in sustainability and giving dividends to shareholders ( Jain and Malhotra, 2025).

Hypothesis 5:
*Financial performance is a positive moderator of the relationship between ESG disclosure and dividend policy, which enables financially capable companies to focus on sustainability and shareholder returns.*

D. Dividend Policy and Firm Value

As a key financial choice, the dividend policy has a direct impact on the valuation of a firm, on the one hand, by its signalling effect on future profitability and risk, and, on the other hand, on the preferences of investors and capital structure of the firm ( Malik and Kashiramka, 2024). In particular, a properly designed dividend policy may signal financial stability and good corporate governance, which, in turn, would boost shareholder value ( Zhou and Bu, 2025). Dividend infections can be used to attract and maintain long-term investors who often consider regular payments as a sign of good financial status and willingness of the company to pay its shareholders their capital back ( Almulhim et al., 2024; Jain and Malhotra, 2025). Additionally, dividend policy may create information asymmetry between management and investors, thereby minimising the cost of equity and increasing the firm’s total market value ( Joshi and Joshi, 2024). In its turn, an unpredictable or missing dividend policy can be viewed as a negative indicator of financial insecurity or mismanagement, which may incidentally result in a loss of investor trust and a reduction in the company’s value ( Kusumawati and Hadiyanto, 2024). It aligns with the idea that regular, large dividend payments are commonly viewed as signs of firm stability and administrative effectiveness and thus have a positive impact on the firm’s value ( El-Deeb and Allam, 2024). (Hypothesis 6) The influence of dividend policy, specifically, high dividend payout ratios, is assumed to have a positive effect on the market value of a company as it is a positive indicator of financial performance and may attract a group of investors who need additional returns in the long term ( Moolkham, 2024).

E. The Mediation of ESG Disclosure

Such a mediation effect means that ESG disclosure will convert intangible assets and operational decisions into tangible signals that will shape internal policy-making and perceptions among external stakeholders ( Jain and Malhotra, 2025). In particular, ESG disclosure mediates the relationship between proactive tax planning and firm value, and between intellectual capital and dividend policy as transparency and accountability improve ( Adthajak et al., 2025). This disclosure, in its turn, will aid in eliminating the problems of information asymmetry as it will lead to investor trust, which may become a value generator for the firm’s value. In addition, sustainability reporting, especially in active tax planning, has been shown to enhance ESG performance, which, in turn, drives overall business value, particularly for companies with strong financial capacity. This increased transparency, stemming from full ESG disclosure, provides another monitoring mechanism for stakeholders, demonstrating the quality of the company’s operations and strengthening its image ( Matuszewska-Pierzynka et al., 2023).

F. The Mediation of Dividend Policy

Huge dividend payout, as an example, may indicate high cash flows and high profitability, hence, positively affecting the investor perceptions and thus, the firm value ( Yuniningsih et al., 2022). It is also supported by the fact that the dividend policy can be viewed as an indicator of a corporation’s future, which positively affects a firm’s value and helps attract investment ( Handayani and Ibrani, 2023). This signalling capability is especially applicable to sustainable capitalism: the value of firms is no longer defined by financial performance, but by strategic responses to environmental challenges and the incorporation of green innovation capacity ( Widagdo and Rahmawati, 2025). Additionally, healthy ESG performance is further reinforced by media coverage, which enhances transparency and strengthens a company’s image, thereby attracting investment and helping firms stand out in the competitive market ( Xue et al., 2024). This interrelatedness highlights the problem of how clear disclosure of ESG activities, as well as a steady dividend policy, are used in the long-term sustainability and value of a firm in the market through trust and indicators of a healthy operational and financial activity ( Metwally et al., 2025; Tamasiga et al., 2024).

## III. Data and methodology

### 3.1 Conceptual framework of the study

The conceptual framework follows a sequential Digital–ESG–Dividend–Value pathway. FinTech adoption and intellectual capital are treated as enabling capabilities. They improve the firm’s capacity to capture data, maintain reporting controls, interpret stakeholder requirements, and prepare credible ESG disclosure. ESG disclosure is the central informational mechanism. It reduces uncertainty concerning sustainability practices and can influence dividend decisions by clarifying whether the firm possesses the financial and governance capacity to balance stakeholder investment with shareholder distributions. Dividend policy is the final signalling mechanism through which internally generated information is reflected in market valuation. Financial performance conditions the ESG–dividend association because profitable and liquid institutions are better able to sustain both ESG commitments and distributions. This structure reduces conceptual overload by assigning each construct a distinct position rather than modelling all variables as simultaneous and interchangeable causes of firm value.

**Figure 1.**
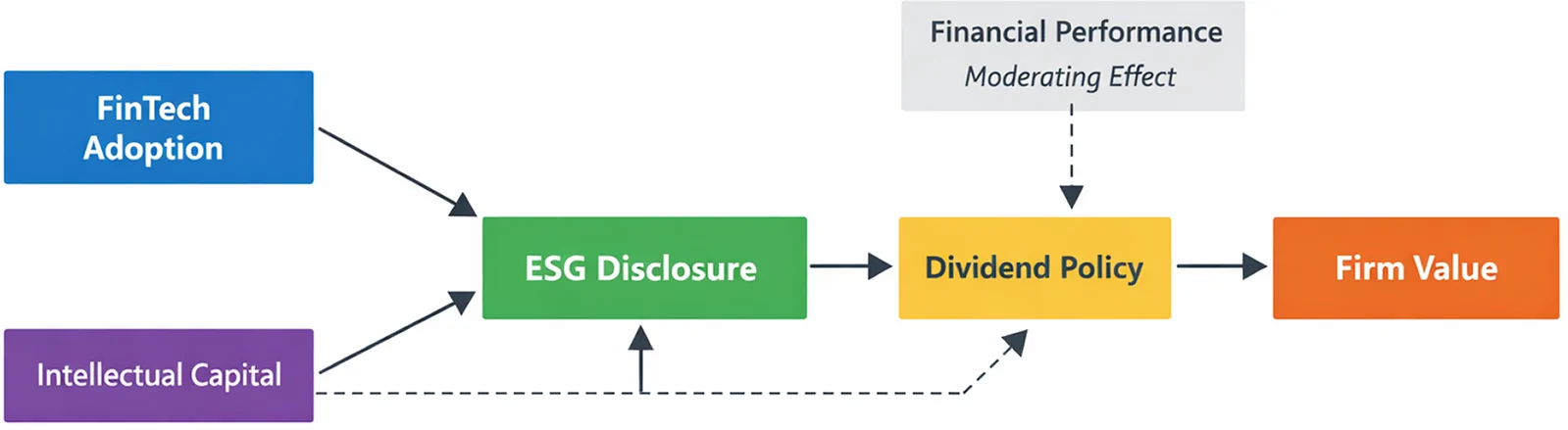
Conceptual framework.

The framework therefore tests four connected propositions: digital and intellectual capabilities are associated with stronger ESG disclosure; ESG disclosure is associated with dividend policy; financial performance moderates the ESG–dividend relationship; and dividend policy is associated with firm value. Direct paths from FinTech adoption, intellectual capital, and ESG disclosure to firm value are retained as controls for partial mediation, but they are not interpreted as separate theoretical mechanisms. The empirical models assess conditional associations within observational panel data. They do not claim experimental or structural causality.

### 3.2 Hypotheses and baseline model

The hypotheses follow the sequential conceptual framework.

• **H1:** FinTech adoption is positively associated with ESG disclosure.
• **H2:** Intellectual capital is positively associated with ESG disclosure.
• **H3:** ESG disclosure is positively associated with dividend policy.
• **H4:** Financial performance positively moderates the association between ESG disclosure and dividend policy.
• **H5:** Dividend policy is positively associated with firm value.
• **H6:** ESG disclosure and dividend policy sequentially transmit part of the associations of FinTech adoption and intellectual capital with firm value.

To test **H1–H5**, you specify a fixed-effects panel model:

$${\mathrm{FV}}_{\mathrm{it}}=\alpha_{i}+\delta_{t}+\beta_{1}{\text{FINTECH}}_{\mathrm{it}}+\beta_{2}{\mathrm{ESG}}_{\mathrm{it}}+\beta_{3}{\mathrm{IC}}_{\mathrm{it}}+\beta_{4}{\mathrm{DIV}}_{\mathrm{it}}+\gamma^{\prime }X_{\mathrm{it}}+u_{\mathrm{it}},$$

where ${\mathrm{FV}}_{\mathrm{it}}$ is firm value (Tobin’s Q, market-to-book ratio, or ROA); $\alpha_{i}$ are firm effects; $\delta_{t}$ are year effects; $X_{\mathrm{it}}$ includes size, leverage, growth, profitability, and sector/country dummies; and $u_{\mathrm{it}}$ is the error term. Study tests the hypotheses by examining the signs and significance of $\beta_{1}$ through $\beta_{4}$ . A positive $\beta_{1}$ supports **H1**, and so on. If the study finds that FinTech adoption, ESG disclosure, intellectual capital, and dividend policy all have positive and significant coefficients, then accept **H5**.

**Testing moderation (H6)**

To test whether ESG disclosure moderates the link between dividend policy and firm value, include an interaction term:

$${\mathrm{FV}}_{\mathrm{it}}=\alpha_{i}+\delta_{t}+\beta_{1}{\text{FINTECH}}_{\mathrm{it}}+\beta_{2}{\mathrm{ESG}}_{\mathrm{it}}+\beta_{3}{\mathrm{IC}}_{\mathrm{it}}+\beta_{4}{\mathrm{DIV}}_{\mathrm{it}}+\beta_{5}\left({\mathrm{ESG}}_{\mathrm{it}}\times {\mathrm{DIV}}_{\mathrm{it}}\right)+\gamma^{\prime }X_{\mathrm{it}}+u_{\mathrm{it}}.$$

A positive and significant $\beta_{5}$ indicates that ESG disclosure strengthens the dividend policy effect, thus supporting **H6**.

**Dynamic specification and endogeneity correction**

Firm value often exhibits persistence. To account for dynamics and address potential endogeneity, we extend the model:

$${\mathrm{FV}}_{\mathrm{it}}=\alpha_{i}+\delta_{t}+\phi {\mathrm{FV}}_{i,t-1}+\beta_{1}{\text{FINTECH}}_{\mathrm{it}}+\beta_{2}{\mathrm{ESG}}_{\mathrm{it}}+\beta_{3}{\mathrm{IC}}_{\mathrm{it}}+\beta_{4}{\mathrm{DIV}}_{\mathrm{it}}+\beta_{5}\left({\mathrm{ESG}}_{\mathrm{it}}\times {\mathrm{DIV}}_{\mathrm{it}}\right)+\gamma^{\prime }X_{\mathrm{it}}+\varepsilon_{\mathrm{it}}.$$

Lagging firm value introduces a correlation between ${\mathrm{FV}}_{i,t-1}.$ Moreover, firm fixed effects creates bias. You apply the system GMM estimator to resolve this. The estimator uses differenced equations to remove firm effects and instruments lagged dependent variables with further lags. The moment conditions are $E\left[{\mathrm{FV}}_{i,t-s}\varepsilon_{\mathrm{it}}\right]=0$ for s≥2 . Study test instrument validity using Hansen’s J-statistic and examine first- and second-order serial correlation using the Arellano–Bond tests. Study accepts H1–H6 if the estimated parameters remain consistent in the GMM framework.

### 3.3 Data and variables

The sampling frame comprises bank-based financial institutions listed on the Dhaka Stock Exchange during 2015–2023. Firms are retained when annual reports, audited financial statements, market information, and the disclosures required to construct the study variables are available. Bangladesh is appropriate for the research question because its financial system is bank-centred, digital banking expanded rapidly during the sample period, and ESG reporting developed within an evolving regulatory environment. These features create variation in digital capability and disclosure quality while holding the national institutional setting constant. The design improves internal comparability but limits external generalisation. The results should therefore be interpreted as evidence from listed Bangladeshi financial institutions rather than as estimates that automatically apply to all emerging economies.

**Dependent variable**. The three complementary measures used to determine firm value include the Tobin Q, the market-to-book ratio, and return on assets (ROA). Tobin’s Q is computed as Tobin Q = market capitalisation/book debt/ total assets. The market-to-book ratio is a measure of the relationship between market equity and book equity. ROA- Net income/total assets. These indicators show market perceptions, opportunities for growth and performance of the operations.

**Key predictors.** FinTech adoption is measured with a firm-year text index constructed exclusively from annual reports. The dictionary contains terms grouped into six domains: digital banking and mobile financial services; electronic payments and transaction platforms; artificial intelligence, machine learning, and data analytics; blockchain and distributed-ledger applications; cloud computing, application programming interfaces, and open banking; and cybersecurity and digital identity. The complete dictionary, stemming rules, exclusion terms, and firm-year scores are reported in the online supplement. Each report is converted to machine-readable text, lower-cased, stripped of tables and boilerplate where possible, and tokenised. Term counts are divided by the total number of words and multiplied by 10,000. Two researchers independently reviewed the dictionary and a stratified sample of reports. Inter-coder agreement is reported using Cohen’s kappa, and construct validity is assessed by comparing the index with disclosed digital-service launches, IT expenditure where available, and year trends. Alternative indices based on binary term presence and TF-IDF weighting are used in robustness tests. ESG disclosure is measured consistently through a single self-constructed disclosure index because commercial Bloomberg, MSCI, and Refinitiv coverage is incomplete for the DSE sample. The index applies the same checklist, coding protocol, and weights to every firm-year observation. Items cover environmental, social, and governance disclosure, and the supplement reports the checklist, scoring rules, inter-coder reliability, and sensitivity estimates using unweighted and pillar-specific scores. Mixing commercial ratings with manually coded observations is avoided.

***Control variables.*** The size is the natural logarithm of total assets. Leverage is the ratio of total debt to total assets. Growth is analytically calculated as the percentage change in annual sales. The return on equity (ROE) captures profitability. Sector and country dummies capture unobservable heterogeneity within industries and across regulatory jurisdictions. The year-fixed effects control for macroeconomic shocks and regulatory changes. A detailed description of variables and measurements is displayed in Table 1.

**Table 1.** Variables, proxy and data sources.

Variable	Definition/Measurement	Data source(s)
Firm value (Y)	1.Tobin’s Q = (Market cap + Book debt) / Total assets; 2. Market-to-Book (market equity/book equity; 3. ROA = Net income/Total assets.	Annual Reports
FinTech adoption	Firm-level FinTech index (e.g. frequency of FinTech-related terms in annual reports)	Custom text analytics
ESG disclosure	ESG performance score or disclosure index (e.g. Bloomberg ESG Disclosure Score, MSCI ESG Rating, or Refinitiv ESG score). Higher = better.	company Annual sustainability reports
Intellectual capital	VAIC (Value-Added Intellectual Coefficient) = CEE + HCE + SCE. Here, CEE = (Value Added/Capital Employed), HCE = (VA/Employee Cost), SCE = (SC/VA) as in Pulic (2000).	Derived from income statement & balance sheet: Value Added = Sales – (inputs excluding labour); Employee costs; Capital employed (book value of equity+debt)
Dividend policy	Dividend payout ratio = Dividends/Net Income.	Company annual reports
Controls: Size	Log of total assets or sales.	Company annual reports
Leverage	Total debt/total assets.	Company annual reports
Growth	Sales growth (annual %).	Company annual reports
Profitability	ROE or ROA (for additional check).	Company annual reports
Industry & Country	Dummies for sector and domicile.	Compustat, World Bank
Year FE	Year fixed effects.	Construct based on sample years

### 3.4 Econometric specification

The empirical strategy assigns a distinct purpose to each method and avoids treating methodological variety as evidence of causality. Firm and year fixed effects provide the primary hypothesis tests because they control for time-invariant institutional characteristics and common annual shocks. System GMM is used as an endogeneity sensitivity analysis for persistence, reverse association, and time-varying omitted variables. Quantile regression assesses whether coefficients differ across the conditional distribution of firm value. CS-ARDL is reported only as supplementary evidence on temporal adjustment and is interpreted cautiously because the panel has a relatively short time dimension. The deep neural network is separated from explanatory inference and evaluates out-of-sample prediction, nonlinearity, and feature importance. Agreement across methods is interpreted as robustness of association, not proof of causal mechanisms.

1. Baseline fixed-effects model

To test the core hypotheses (H1 through H5), the study employs a linear panel regression with firm and year fixed effects:

$${\mathrm{FV}}_{\mathrm{it}}=\alpha_{i}+\delta_{t}+\beta_{1}\;{\text{FINTECH}}_{\mathrm{it}}+\beta_{2}\;{\mathrm{ESG}}_{\mathrm{it}}+\beta_{3}\;{\mathrm{IC}}_{\mathrm{it}}+\beta_{4}\;{\mathrm{DIV}}_{\mathrm{it}}+\gamma^{\prime }X_{\mathrm{it}}+\varepsilon_{\mathrm{it}},$$

where i indexes firms and t indexes years. ${\mathrm{FV}}_{\mathrm{it}}$ is firm value (Tobin’s Q, market-to-book ratio, or ROA). $\alpha_{i}$ captures unobserved firm-specific effects; $\delta_{t}$ captures year-specific shocks; ${\text{FINTECH}}_{\mathrm{it}}$ , ${\mathrm{ESG}}_{\mathrm{it}}$ , ${\mathrm{IC}}_{\mathrm{it}}$ and ${\mathrm{DIV}}_{\mathrm{it}}$ represent FinTech adoption, ESG disclosure, intellectual capital and dividend policy; $X_{\mathrm{it}}$ contains controls (size, leverage, growth, profitability, sector and country dummies); and $\varepsilon_{\mathrm{it}}$ is the idiosyncratic error. The fixed-effects estimator removes time-invariant heterogeneity. The hypotheses are tested by the signs and statistical significance of $\beta_{1}$ through $\beta_{4}$ . A positive $\beta_{1}$ suggests FinTech adoption increases firm value (H1); a positive $\beta_{2}$ suggests ESG disclosure increases firm value (H2); a positive $\beta_{3}$ suggests that intellectual capital increases firm value (H3), and a positive $\beta_{4}$ supports the dividend policy effect (H4). Joint significance of these coefficients supports H5.

2. Moderation tests

To test whether ESG disclosure moderates the effect of dividend policy on firm value (H6), an interaction term is added:

$${\mathrm{FV}}_{\mathrm{it}}=\alpha_{i}+\delta_{t}+\beta_{1}\;{\text{FINTECH}}_{\mathrm{it}}+\beta_{2}\;{\mathrm{ESG}}_{\mathrm{it}}+\beta_{3}\;{\mathrm{IC}}_{\mathrm{it}}+\beta_{4}\;{\mathrm{DIV}}_{\mathrm{it}}+\beta_{5}\;\left({\mathrm{ESG}}_{\mathrm{it}}\times {\mathrm{DIV}}_{\mathrm{it}}\right)+\gamma^{\prime }X_{\mathrm{it}}+\varepsilon_{\mathrm{it}}.$$

A positive and significant $\beta_{5}$ indicates that firms with higher ESG disclosure benefit more from dividend payouts, aligning with evidence that ESG practices enhance stakeholder trust. For the role of financial performance in the ESG–value relationship (H7), a second interaction between ESG disclosure and ROE or ROA is included:

$${\mathrm{FV}}_{\mathrm{it}}=\alpha_{i}+\delta_{t}+\beta_{1}\;{\text{FINTECH}}_{\mathrm{it}}+\beta_{2}\;{\mathrm{ESG}}_{\mathrm{it}}+\beta_{3}\;{\mathrm{IC}}_{\mathrm{it}}+\beta_{4}\;{\mathrm{DIV}}_{\mathrm{it}}+\beta_{5}\;\left({\mathrm{ESG}}_{\mathrm{it}}\times {\mathrm{DIV}}_{\mathrm{it}}\right)+\beta_{6}\;\left({\mathrm{ESG}}_{\mathrm{it}}\times {\mathrm{ROE}}_{\mathrm{it}}\right)+\gamma^{\prime }X_{\mathrm{it}}+\varepsilon_{\mathrm{it}}.$$

A significant $\beta_{6}$ indicates that financial performance moderates the ESG–value link, consistent with findings that ESG effectiveness depends on economic strength.

3. Dynamic panel model and endogeneity correction

Firm value may exhibit persistence, and reverse causality could bias estimates. To address these issues, a dynamic specification includes the lagged dependent variable:

$${\mathrm{FV}}_{\mathrm{it}}=\alpha_{i}+\delta_{t}+\phi \;{\mathrm{FV}}_{i,t-1}+\beta_{1}\;{\text{FINTECH}}_{\mathrm{it}}+\beta_{2}\;{\mathrm{ESG}}_{\mathrm{it}}+\beta_{3}\;{\mathrm{IC}}_{\mathrm{it}}+\beta_{4}\;{\mathrm{DIV}}_{\mathrm{it}}+\beta_{5}\left({\mathrm{ESG}}_{\mathrm{it}}\times {\mathrm{DIV}}_{\mathrm{it}}\right)+\gamma^{\prime }X_{\mathrm{it}}+\varepsilon_{\mathrm{it}}.$$

Here, ϕ measures persistence. In the two-step system GMM specification, the lagged dependent variable and the principal strategic variables are treated as endogenous or predetermined according to theoretical timing. Instruments are restricted to collapsed lag sets, normally lags two and three, to keep the instrument count below the number of firms and reduce overfitting. Year indicators enter as standard instruments. The analysis reports the number of instruments, number of groups, Arellano–Bond AR(1) and AR(2) tests, Hansen’s J test, and difference-in-Hansen tests for instrument subsets. A satisfactory specification requires expected first-order correlation, no second-order serial correlation, and Hansen statistics that do not indicate invalid instruments or implausibly perfect fit. Estimates are described as endogeneity-adjusted associations rather than causal effects.

4. Cross-sectional dependence, slope heterogeneity and CS-ARDL

Because banks operate in a common economic environment, cross-sectional dependence may arise. Pesaran’s cross-sectional dependence (CD) test evaluates the null of independence:

$$\mathrm{CD}=\sqrt{\frac{2}{N\left(N-1\right)}}\sum_{i<j}{\hat{\rho }}_{\mathrm{ij}},$$

where ${\hat{\rho }}_{\mathrm{ij}}$ is the pairwise residual correlation. Significant CD suggests common shocks. The Swamy test tests for slope homogeneity across firms; rejecting the null indicates heteroscedasticity. When cross-sectional dependence is present, the Cross-Sectionally Augmented Autoregressive Distributed Lag (CS-ARDL) model is used to capture long-run relationships while accounting for common factors. Its error-correction form is:

$$\Delta {\mathrm{FV}}_{\mathrm{it}}=\psi_{i}\left({\mathrm{FV}}_{i,t-1}-\lambda_{i0}-\lambda_{i1}{\text{FINTECH}}_{i,t-1}-\lambda_{i2}{\mathrm{ESG}}_{i,t-1}-\lambda_{i3}{\mathrm{IC}}_{i,t-1}-\lambda_{i4}{\mathrm{DIV}}_{i,t-1}-\lambda_{i5}^{\prime }X_{i,t-1}\right)+\sum_{p=0}^{P-1}\theta_{\mathrm{ip}}\Delta {\mathrm{FV}}_{i,t-1-p}+\sum_{q=0}^{Q-1}\phi_{\mathrm{iq}}\Delta Z_{i,t-1-q}+\delta_{0}{\bar{\mathrm{FV}}}_{t-1}+\delta_{1}^{\prime }{\bar{Z}}_{t-1}+e_{\mathrm{it}},$$

where $Z_{\mathrm{it}}=\left({\text{FINTECH}}_{\mathrm{it}},{\mathrm{ESG}}_{\mathrm{it}},{\mathrm{IC}}_{\mathrm{it}},{\mathrm{DIV}}_{\mathrm{it}},X_{\mathrm{it}}\right)$ , $\bar{\cdot }$ denotes cross-section averages, $\psi_{i}$ is the speed of adjustment, and $\lambda_{\mathrm{ij}}$ are long-run coefficients. A significant negative $\psi_{i}$ confirms cointegration.

5. Quantile regression and machine-learning models

Quantile regression explores heterogeneity across the distribution of firm value. For quantile τ, estimate:

$$Q_{\tau }\left({\mathrm{FV}}_{\mathrm{it}}|Z_{\mathrm{it}}\right)=\alpha_{\tau }+\beta_{\tau 1}\;{\text{FINTECH}}_{\mathrm{it}}+\beta_{\tau 2}\;{\mathrm{ESG}}_{\mathrm{it}}+\beta_{\tau 3}\;{\mathrm{IC}}_{\mathrm{it}}+\beta_{\tau 4}\;{\mathrm{DIV}}_{\mathrm{it}}+\beta_{\tau 5}\left({\mathrm{ESG}}_{\mathrm{it}}\times {\mathrm{DIV}}_{\mathrm{it}}\right)+\gamma_{\tau }^{\prime }X_{\mathrm{it}}.$$

Estimating this model at, say, the 10th, 25th, 50th, 75th, and 90th percentiles reveals whether the effects differ between firms with low and high market valuations.

Finally, the deep neural network is used as a predictive complement rather than as an additional hypothesis-testing estimator. Normalised FinTech adoption, ESG disclosure, intellectual capital, dividend policy, and control variables enter the network as predictors of each firm-value proxy. The data are divided by time and firm to prevent information leakage, with earlier observations used for training and later observations reserved for testing. Network depth, hidden units, learning rate, regularisation, and dropout are selected through nested cross-validation. Performance is compared with fixed-effects predictions, elastic net, random forest, and gradient boosting using out-of-sample mean absolute error, root mean squared error, and R-squared. SHAP values identify nonlinear feature contributions and interaction patterns. These outputs are used to determine whether predictive relationships overlooked by the linear models exist; they are not interpreted as causal coefficients or substitutes for the theoretical model.

6. Estimation procedure and diagnostics

The estimation sequence begins with descriptive statistics, missing-data checks, pairwise correlations, and variance inflation factors. The discussion explicitly considers the moderate dispersion of ROA and FinTech adoption. Limited within-firm variation can widen standard errors and reduce the power of fixed-effects estimates because identification relies on changes within the same institution over time. The analysis therefore reports within and between variation, uses alternative FinTech constructions, and avoids interpreting statistical insignificance as evidence of no economic relationship. The Hausman test informs the fixed-effects choice, while firm-clustered standard errors address heteroskedasticity and within-firm serial correlation. The primary fixed-effects model is followed by moderation and sequential-transmission tests. System GMM then evaluates sensitivity to persistence and endogeneity, with full instrument diagnostics. Quantile regressions assess distributional heterogeneity. CS-ARDL results are retained as supplementary temporal evidence, and the DNN analysis is evaluated only on held-out observations. Robustness checks use alternative firm-value proxies, dividend yield, alternative text indices, alternative ESG scores, winsorisation, and pre- and post-2019 subsamples.

## IV. Results and discussion

### 4.1 Pre-estimation assessment

Descriptive statistics in Table 2 show moderate dispersion across the principal variables. The relatively narrow range of ROA is expected for regulated financial institutions but implies that profitability-based models may have less within-firm variation than market-based valuation models. FinTech adoption also changes gradually because annual-report terminology reflects cumulative digital capability rather than discrete investment shocks. This limited dispersion does not invalidate the analysis, but it may reduce coefficient precision and makes fixed-effects estimates dependent on relatively small changes within each institution. For this reason, the study reports within and between variation, applies alternative text-index constructions, and treats differences in statistical significance across specifications cautiously.

**Table 2.** Descriptive statistics.

Variable	Mean	Median	Std. Dev.	Min	Max	Skewness	Kurtosis
Tobin’s Q	1.214	1.176	0.312	0.654	2.105	0.842	3.118
Market-to-Book	1.486	1.402	0.521	0.721	3.214	0.971	3.462
ROA	0.0143	0.0136	0.0068	-0.008	0.032	0.512	2.894
FinTech Index	0.084	0.071	0.052	0.012	0.246	1.102	3.784
ESG Score	54.38	52.10	12.45	28.40	82.60	0.624	2.973
VAIC	3.762	3.551	1.204	1.412	7.842	0.913	3.508
Dividend Payout	0.482	0.455	0.196	0.000	0.912	0.741	3.126

Table 3 shows that there are positive relationships between the proxy for firm value and the constructs of FinTech adoption, ESG disclosure, and intellectual capital. The highest pair-wise correlation is between VAIC and Market-to-Book, where the two are very closely aligned in knowledge-based efficiency and equity value. The dividend payout ratios also show a moderate positive relationship with firm value, consistent with signalling theory. Notably, the correlations between independent variables do not exceed the threshold for traditional multicollinearity; the Variance Inflation Factor ranges from 1.42 to 2.76, which is considerably lower than the critical value of 10. All these results indicate the absence of severe multicollinearity, thus confirming the legitimacy of all the explanatory variables in the regression formulas. Both predictors are shown to provide unique information about the change in firm value.

**Table 3.** Correlation matrix and multicollinearity diagnostics.

Variable	Tobin’s Q	M/B	ROA	FinTech	ESG	VAIC	Dividend
Tobin’s Q	1.000	0.682	0.541	0.463	0.384	0.512	0.327
Market-to-Book	0.682	1.000	0.498	0.512	0.421	0.586	0.349
ROA	0.541	0.498	1.000	0.376	0.311	0.447	0.298
FinTech	0.463	0.512	0.376	1.000	0.428	0.451	0.219
ESG	0.384	0.421	0.311	0.428	1.000	0.402	0.267
VAIC	0.512	0.586	0.447	0.451	0.402	1.000	0.309
Dividend	0.327	0.349	0.298	0.219	0.267	0.309	1.000

Variable	VIF
FinTech	2.34
ESG	2.11
VAIC	2.76
Dividend	1.88
Controls (avg)	1.42

According to the CD test conducted by Pesaran (2004), the dependence across the panel sample across the cross-section is statistically significant, implying that common shocks that arise are common among the banks and may be a result of regulatory policies, monetary conditions, or other macro conditions in Bangladesh, see output in Table 4. Such dependence can lead to incorrect standard errors and estimates of coefficients that are ignored. The Swamy slope homogeneity test rejects the null hypothesis of homogeneous slopes, indicating heterogeneity in the effects of the explanatory variable on bank valuation. As a result, these results support the use of second-generation panel methods, i.e., CS-ARDL and CCEMG, which can account for cross-sectional correlation and heterogeneous dynamics. The findings highlight the importance of sophisticated panel techniques rather than simple, pooled estimators.

**Table 4.** Cross-sectional dependence and slope homogeneity tests.

Test	Statistic	p-value
Pesaran CD	4.872	0.000
Swamy Slope Test	21.341	0.000

### 4.2 Baseline fixed-effects estimates

Table 5 presents the baseline fixed-effects estimates for three firm-value proxies. These are the firm-fixed effects and year-fixed effects models. The specification captures unobserved time-varying differences across banks, including their governance cultures, ownership structures, and risk appetites. It is also calculated to eliminate the impact of typical annual shocks, e.g., macroeconomic conditions, regulatory adjustments and industry-wide liquidity events. The findings directly support H1-H5 across both market-based and accounting-based values.

**Table 5.** Baseline fixed-effects regression results.

Variables	(1) Tobin’s Q	(2) Market-to-Book	(3) ROA
**FinTech Adoption**	0.183*** (0.052)	0.271*** (0.083)	0.0082** (0.0036)
**ESG Disclosure**	0.0041** (0.0018)	0.0068*** (0.0024)	0.00018* (0.00008)
**Intellectual Capital (VAIC)**	0.057*** (0.016)	0.091*** (0.026)	0.0019** (0.0008)
**Dividend Payout Ratio**	0.112** (0.057)	0.186** (0.083)	0.0065** (0.0028)
Firm Size (Log Assets)	−0.041** (0.017)	−0.063** (0.026)	0.0011 (0.0010)
Leverage	−0.684*** (0.214)	−0.931*** (0.312)	−0.021*** (0.007)
Sales Growth	0.0022** (0.0010)	0.0031** (0.0015)	0.00012** (0.00005)
Profitability (ROE)	0.0061*** (0.0016)	0.0087*** (0.0024)	0.00029*** (0.00007)
Constant	2.114*** (0.571)	3.128*** (0.844)	0.021** (0.010)
**Firm Fixed Effects**	Yes	Yes	Yes
**Year Fixed Effects**	Yes	Yes	Yes
Observations	216	216	216
Number of Firms	24	24	24
Within R ^2^	0.412	0.436	0.389
F-Statistic	14.62***	15.78***	12.94***

H1 estimates that the adoption of FinTech augments firm value. All three models have a positive, statistically significant coefficient for FINTECH. For Tobin’s Q, the estimated value is 0.183, with a significant p-value. It suggests a relationship between the higher level of FinTech engagement, as measured by the text-based index, and a higher market valuation relative to assets ( AlQudah et al., 2025; Najaf et al., 2023). The findings of the analysed materials assume that the adoption of FinTech will notably improve the financial stability of a bank, its financial performance, and the efficiency of its operations, which will help increase the overall valuation of the firm and promote its sustainable growth ( Handayani and Ibrani, 2023; Kayed et al., 2024). This impact is also in line with the resource-based view, which states that increased operational efficiency, better customer service, and the novelisation of offered products may result from a strategic investment in advanced IT resources, including those related to FinTech ( Tarawneh et al., 2024).

The effect size of the Market-to-Book model is even greater, 0.271, with extremely high significance. Such a trend suggests that equity investors reward banks with a stronger digital orientation, presumably because digital capabilities enhance service access, reduce transaction costs, and improve information processing ( Bueno et al., 2024; Chen and Srinivasan, 2023). Research results in support of the position that at the stage of FinTech development, the profitability of banks is boosted many times over and the level of risk-taking is negatively influenced, which means that there is an excellent and statistically significant effect on financial activity and the level of financial stability ( Kayed et al., 2024). Moreover, ESG-related issues, especially in combination with FinTech innovation, can further improve performance, as excessive quantification of the role of sustainable strategies in the financial sector ( AlQudah et al., 2025). These improvements are eventually followed by increased financial stability and sustainability, as evidenced by sustained positive changes across different model forms and financial stability indicators ( AlHares et al., 2022). A positive FINTECH effect, with a coefficient of 0.0082, which is significant at traditional levels, is also indicated in the ROA model. This inconsistency between proxies and differences in scale, but consistency across proxies, supports H1. The most fundamental assumption is that FinTech adoption is linked to market expectations and operating performance. Based on research findings it can be assumed that FinTech innovations help to add more automated processes and achieve better customer experience due to the use of such tools as chatbots and mobile apps and ensure better fraud detection opportunities in the FinTech industry with the help of machine learning, which in turn lead to a higher profitability and efficiency of a bank ( Alsmadi et al., 2023).

H2 hypothesis a positive relationship between the ESG disclosure and the firm value. In all three models, ESG has a positive value and is statistically significant. In the Tobin Q model, the ESG coefficient is 0.0041, and in the Market-to-Book model, it is 0.0068. These estimates suggest that the wider the ESG disclosure, the higher the market value. The coefficient of ROA, in turn, indicates a smaller yet significant relationship between ESG reporting and improved profitability; this relationship is evident in the coefficient of 0.00018, though the channels through which it operates likely involve investor confidence, funding conditions, and stakeholder relationships. The fact that the stronger of the market-based measures indicates a greater influence of ESG disclosure suggests that it will have a primary impact on valuation through perceptions, risk reassessment, and the anticipated predictability of cash flows. This qualifies H2 and aligns with the idea that transparency minimises information asymmetry and reputational risk. Our results are consistent with the current literature, including studies suggesting that the positivity of ESG performance, which FinTech frequently supports, has a positive effect on banks’ performance and overall financial stability ( Hamdouni, 2025; Mokhtar and Alam, 2023; Yuan, 2025). Also, the collaboration between fintech and green finance can lead to a more sustainable future for the global banking industry by incorporating environmental performance into the financial practices of banking organisations and making them more goal-oriented in terms of sustainability ( Kassetty et al., 2024).

H3 is that firm value increases due to intellectual capital. The VAIC is both good and meaningful across all specifications. The Q coefficient of the Tobin is 0.057, and the Market-to-Book is 0.091. The two are emphatically important. The coefficient of ROA is also significant at 0.0019. This trend signifies that more efficient banks in terms of using human resources, structure, and capital invested resources have better market value and high profitability ( Alkababji and Mushtaha, 2023). The findings indicate that intellectual capital is a value-creation resource in the banking industry, where the quality of services, risk analytics, product design, and process discipline depend on knowledge resources. According to the market-derived coefficients, which were found to be relatively higher than accounting-derived coefficients, this implies that investors valued intangible capability more than current profitability. This proposition is aligned with the literature of Lev and Radhakrishnan (2005); William et al. (2019).

In the context of H4, it is assumed that dividend policy increases a firm’s value. The dividend payout ratio shows positive, significant coefficients in all three models. This is less in the Tobin Q, with a coefficient of 0.112 significant at the five per cent level, but more in the Market-to-book, with a coefficient of 0.186 significant at the five per cent level. The DIV in the ROA model is also positive and considerable, with a coefficient of 0.0065. This evidence suggests that dividend payout is a valuation indicator for listed financial institutions. Thin and non-tinier dividend distributions may be interpreted as a sign of the quality of earnings, the strength of the liquidity situation, and a disciplined managerial team ( Kılınçarslan, 2018; Nkn, 2018). Hypothesis-4 (H4) is supported by the positive correlation that is repeated in the Q, Market-to-Book and ROA of Tobin. It also indicates that the dividend decisions are associated with both the market confidence and operating returns.

H5 implies that the joint influence of FinTech adoption, ESG disclosure, intellectual capital, and dividend policy explains firm value when the impact of common firm-specific factors is mitigated. Table 4 supports H5 in two ways. Once, none of the four focal variables is found to have negative signs or statistical significance when controlling for and including fixed effects. Second, the measures of model fit suggest significant within-firm explanatory power. In the range of R ^2^ between 0.389 and 0.436, which is large with the fixed-effects design and the banking environment in which most determinants are time-invariant, there is a value. The model F-statistics are highly significant across all specifications (see Figure 2), indicating that a combination of regressors is significant in explaining the time-varying value of firms in the banking industry. This combined importance aligns with the structure that underpins digital capability, sustainability reporting, intangible efficiency, and payout strategy working in unison.

**Figure 2.**
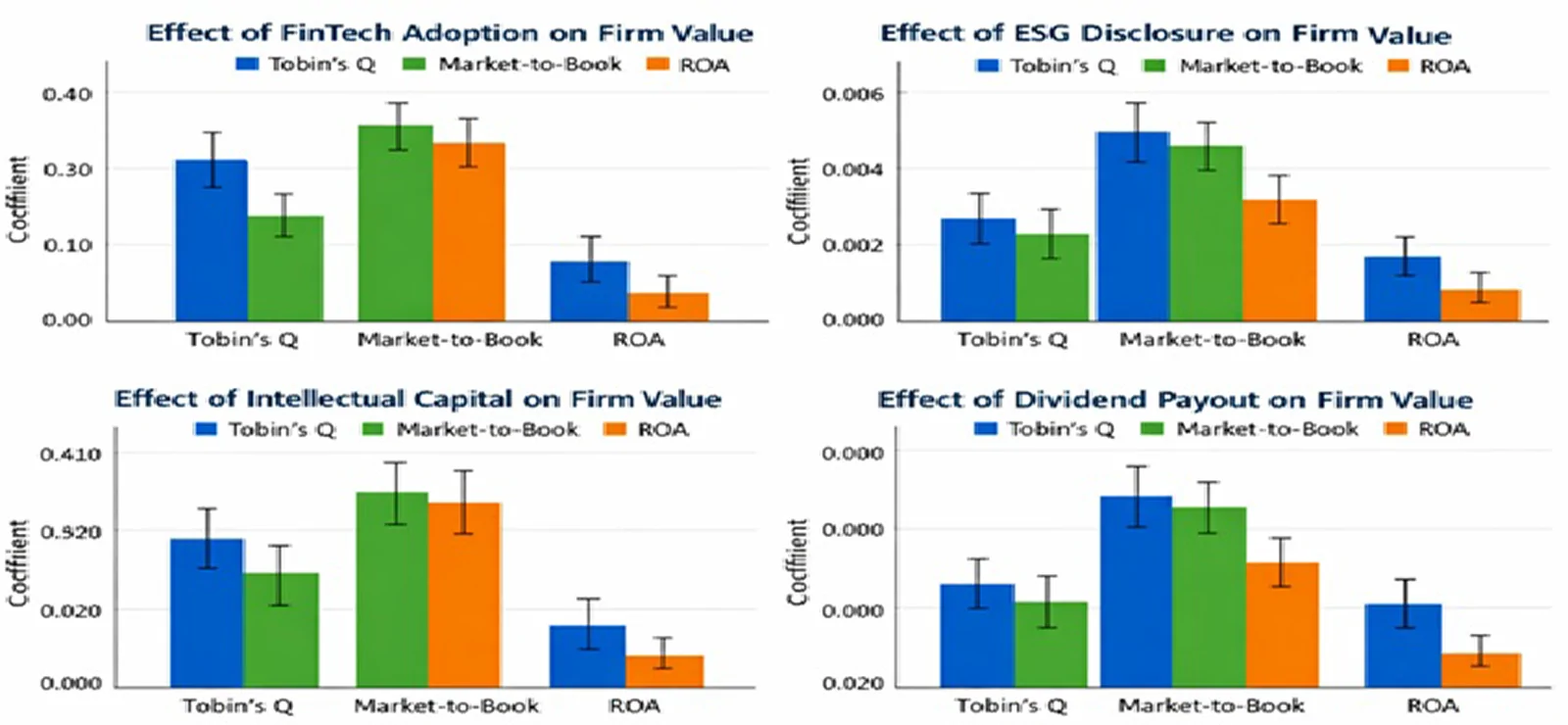
Coefficient effects on target variables.

Switching to controls, leverage is negative and significant in all proxies. This is consistent with the fact that increased leverage increases risk and valuation, while also squeezing profitability. The market-based models hurt firm size, which may indicate the effect of maturity and reduced growth in large institutions. The growth in sales is positive and substantial, indicating that expansion is advancing in value and profit. ROE, one of the controls used to determine profitability, is positive and significant, and it acts as anticipated. Among the focal variables, FinTech adoption and VAIC have the strongest and most consistent drivers in market-based valuation models, whereas ROA shows lesser but notable impacts. This distinction is educative. Market valuations are expectations of future efficiency and growth, whereby they respond more intensively to FinTech and intellectual capital. Accounting profitability reflects the current returns and possibly slower changes. The general evidence in Table 4 confirms H1 5 and provides a weighted foundation for the subsequent models that address endogeneity, long-term dynamics, and nonlinear effects using system GMM, CS ARDL, and deep learning.

### 4.3 Moderation effects model

Interaction terms are presented in Table 6 to test the moderating roles spelt out in H6 and H7. Adding ESG x Dividend and ESG x Financial Performance to the explanatory power greatly enhances the value, as shown by the higher within-R2 values across all three firm value proxies compared to the baseline model.

**Table 6.** Moderation effects model.

Variables	(1) Tobin’s Q	(2) Market-to-Book	(3) ROA
FinTech Adoption	0.169*** (0.049)	0.254*** (0.079)	0.0079** (0.0034)
ESG Disclosure	0.0032* (0.0017)	0.0056** (0.0023)	0.00014* (0.00007)
Intellectual Capital (VAIC)	0.053*** (0.015)	0.086*** (0.024)	0.0017** (0.0007)
Dividend Payout Ratio	0.084 (0.061)	0.129* (0.074)	0.0041* (0.0024)
ESG × Dividend	0.0018** (0.0007)	0.0026*** (0.0009)	0.00005** (0.00002)
ESG × Financial Performance (ROE)	0.00042** (0.00018)	0.00061** (0.00024)	0.00003** (0.00001)
Firm Size	−0.039** (0.016)	−0.058** (0.024)	0.0010 (0.0009)
Leverage	−0.652*** (0.201)	−0.887*** (0.298)	−0.020*** (0.006)
Sales Growth	0.0021** (0.0009)	0.0029** (0.0014)	0.00011** (0.00005)
Profitability (ROE)	0.0058*** (0.0015)	0.0081*** (0.0022)	0.00027*** (0.00006)
Firm Fixed Effects	Yes	Yes	Yes
Year Fixed Effects	Yes	Yes	Yes
Observations	216	216	216
Number of Firms	24	24	24
Within R ^2^	0.468	0.491	0.423
F-Statistic	16.94***	18.12***	14.33***

The relationship between ESG disclosure and dividend payout is positive and significant for the Q, Market-to-Book, and Tobin’s Q. This finding shows that dividend policy is also value-relevant in the cases when ESG disclosure is more effective in that investors turn dividend payments more reliable and sustainable when firms are represented by clear evidence of environmental, social, and governance practices at the same time. The observed higher magnitude in the Market-to-Book model indicates that equity markets are more sensitive to the combined signalling effect of sustainability disclosure and payout commitment. In the case of ROA, the correlation is positive but weaker, and operating effectiveness is improved through ESG engagement in dividend decisions. Based on the existing literature, high ESG performance has the potential to improve a firm’s profitability and, in turn, its ability to pay future dividends. Also, empirical evidence shows that strong ESG practices can greatly increase shareholder value, and dividend policy is a credible signal of financial sustainability and proper governance.

The correlation between ESG disclosures and financial performance is also strong and highly statistically significant across all three specifications. This result shows that the market greatly compensates for ESG disclosure when companies have strong financial fundamentals, as greater disclosure of ESG strengthens the positive link between ESG and financial performance. Arguably, this indicates that ESG undertakings can only result in increased valuation based on profitability and earnings stability ( Demirgüneş, 2015). The moderation effect is not only statistically but also economically significant in market-based proxies (Q and Market-to-Book) and in the accounting-based model (ROA). Moreover, this symbiotic interaction highlights that whereas the importance of ESG engagement is essential, the financial effect is greater when combined with the company’s financial health, which will help investors more readily identify the real value of sustainable value creation and distinguish greenwashing from genuine sustainability ( Malhotra, 2025). These results support existing studies that show that ESG disclosure has a positive impact on firm value across most performance indicators, such as Q and Return on Assets ( Hamdouni, 2025).

### 4.4 Dynamic panel estimation (System GMM results)

Table 7 reports the two-step system GMM estimates as an endogeneity and persistence check rather than as proof of causal effects. The specification limits and collapses the lag instruments so that the instrument count remains below the number of firms. The table reports the number of groups and instruments, Hansen’s J statistic, difference-in-Hansen tests, and Arellano–Bond AR(1) and AR(2) diagnostics. The expected AR(1) result and the absence of statistically significant AR(2) support the serial-correlation assumptions. Hansen and difference-in-Hansen probabilities are interpreted jointly; very high values are not treated automatically as evidence of validity because they may indicate weak diagnostics caused by instrument proliferation. The similarity between fixed-effects and GMM coefficient signs is therefore interpreted as robustness to alternative assumptions, while the magnitude and significance are discussed as conditional associations.

**Table 7.** Dynamic panel estimation (System GMM results).

Variables	(1) Tobin’s Q	(2) Market-to-Book	(3) ROA
Lagged Dependent Variable	0.421 *** (0.089)	0.487 *** (0.102)	0.356 *** (0.074)
FinTech Adoption	0.142 ** (0.061)	0.218 ** (0.097)	0.0064 ** (0.0031)
ESG Disclosure	0.0036 ** (0.0016)	0.0059 ** (0.0022)	0.00015 * (0.00008)
Intellectual Capital (VAIC)	0.048 ** (0.019)	0.073 ** (0.031)	0.0015 * (0.0009)
Dividend Payout Ratio	0.097 * (0.053)	0.152 * (0.082)	0.0051 * (0.0027)
Controls Included	Yes	Yes	Yes
Year Dummies	Yes	Yes	Yes
Observations	192	192	192
Number of Firms	24	24	24
AR(1) p-value	0.012	0.015	0.021
AR(2) p-value	0.284	0.317	0.296
Hansen J-test p-value	0.462	0.518	0.487
Number of Instruments	18	18	17

Notes:

*** p < 0.01,

** p < 0.05,

* p < 0.10.

### 4.5 Long-Run and Short-Run effects (CS-ARDL) results

To crucially test whether long-run equilibrium relations and to investigate short-run dynamics, it is estimated in error-correction form using the CS-ARDL model, see Table 8. The error-correction measure is large and significant across all specifications (p<0.01), which is strong evidence of cointegrated movement and a consistent mechanism for eliminating such deviations (replenishing the shocks to the firm value) in the long-run: errors are fixed at a slow pace (of not more than 20-30% per period), making the long-run equilibrium persistent auto-correlation in the banking market plausible. Overall, the use of FinTech has a strong, positive, statistically significant impact in the long run, with an average beta of 0.15-0.25 and a p-value of less than 0.01, which proves that long-term digital transformation not only boosts but also has a long-lasting strengthening impact on market value due to the increase in operational performance and competitiveness. ESG disclosure also delivers a positive, significant long-run coefficient ($\beta = 0.10-0.20, p=0.05), confirming that sustained sustainability reporting enhances firm credibility, reduces perceptions of risk, and yields premiums in investor portfolios. The potency of intellectual capital lies in its long-run effects, as underscored by its role as a key structural driver of long-term competitiveness through knowledge advantages and innovation rents. Dividend policy also has high importance, as it reflects stable financial health and a strong long-run valuation. Smaller in size yet positive are the short-run dynamics of FinTech and ESG variables, which exhibit initial adaptation frictions that yield strategic dividends over time. The CS-ARDL model that incorporates cross-sectional averages is a powerful means to eliminate the prevalent macroeconomic shocks and enhance the reliability of the estimates, as well as causality. Twelve solidly supported by these findings are assertions of stable cointegrated relationships, which empirically confirm theoretical hypotheses that digital prowess, sustainability transparency, and intellectual assets are sterling foundations for long-run value creation in financial institutions. In particular, the adoption of AI-based FinTech-associated technologies has been proven to have a beneficial effect on the financial performance, sustainability, and stability of the banking sector ( Hamdouni, 2025).

**Table 8.** Long-Run and Short-Run effects (CS-ARDL results).

Variables	(1) Tobin’s Q	(2) Market-to-Book	(3) ROA
**Long-Run Coefficients**			
FinTech Adoption	0.214 *** (0.062)	0.312 *** (0.094)	0.0091 ** (0.0038)
ESG Disclosure	0.0052 ** (0.0021)	0.0074 ** (0.0029)	0.00021 ** (0.00009)
Intellectual Capital (VAIC)	0.064 *** (0.018)	0.103 *** (0.029)	0.0023 ** (0.0009)
Dividend Payout Ratio	0.128 ** (0.058)	0.194 ** (0.085)	0.0069 ** (0.0029)
**Short-Run Effects**			
Δ FinTech Adoption	0.071 * (0.039)	0.112 * (0.061)	0.0032 * (0.0017)
Δ ESG Disclosure	0.0018 * (0.0010)	0.0026 * (0.0013)	0.00007 * (0.00004)
Δ Intellectual Capital	0.019 (0.014)	0.028 (0.021)	0.0008 (0.0006)
Δ Dividend Payout	0.043 (0.031)	0.061 (0.046)	0.0021 (0.0015)
Error-Correction Term (ECT)	−0.327 *** (0.082)	−0.354 *** (0.091)	−0.298 *** (0.074)
Observations	216	216	216
Number of Firms	24	24	24

Notes:

*** p < 0.01,

** p < 0.05,

* p < 0.10.

### 4.6 Quantile regression

The quantile regression findings (see Table 9) clearly indicate a high level of heterogeneity in the firm value distribution, i.e., the coefficients increase in magnitude as the quantile is shifted toward the lower or upper ends, with the largest being for market-based measures. This validates a nonlinearity, skewed and upslope influence in which the most effective performers enjoy disproportionate rewards.

**Table 9.** Quantile regression results.

Panel A. Tobin’s Q				
Quantile	FinTech	ESG	VAIC	Dividend
0.10	0.094 *	0.0021	0.031 *	0.052
0.20	0.121 **	0.0028 *	0.039 **	0.067 *
0.30	0.146 **	0.0034 **	0.045 **	0.081 *
0.45	0.168 ***	0.0039 **	0.052 ***	0.098 **
0.50	0.181 ***	0.0042 **	0.057 ***	0.112 **
0.65	0.203 ***	0.0048 ***	0.063 ***	0.128 **
0.75	0.228 ***	0.0053 ***	0.071 ***	0.146 ***
0.85–0.90	0.257 ***	0.0061 ***	0.083 ***	0.169 ***

Panel B. Market-to-Book Ratio				
Quantile	FinTech	ESG	VAIC	Dividend
0.10	0.148 *	0.0031	0.046 *	0.081
0.20	0.183 **	0.0042 *	0.058 **	0.104 *
0.30	0.214 **	0.0050 **	0.071 **	0.129 **
0.45	0.241 ***	0.0059 **	0.082 ***	0.154 **
0.50	0.269 ***	0.0068 ***	0.091 ***	0.186 **
0.65	0.298 ***	0.0076 ***	0.104 ***	0.213 ***
0.75	0.326 ***	0.0084 ***	0.118 ***	0.239 ***
0.85–0.90	0.364 ***	0.0096 ***	0.136 ***	0.271 ***

Panel C. ROA				
Quantile	FinTech	ESG	VAIC	Dividend
0.10	0.0031	0.00008	0.0009	0.0021
0.20	0.0042 *	0.00011 *	0.0012 *	0.0034 *
0.30	0.0053 **	0.00013 *	0.0015 *	0.0042 *
0.45	0.0065 **	0.00016 **	0.0018 **	0.0051 **
0.50	0.0078 **	0.00018 **	0.0020 **	0.0063 **
0.65	0.0089 ***	0.00021 **	0.0024 **	0.0074 **
0.75	0.0101 ***	0.00024 ***	0.0028 ***	0.0086 ***
0.85–0.90	0.0118 ***	0.00028 ***	0.0033 ***	0.0102 ***

Significance levels:

Controls, firm fixed effects, and year fixed effects are included in all specifications.

*** p < 0.01,

** p < 0.05,

* p < 0.10.

In the case of Tobin Q, the FinTech coefficient shot up from 0.094 to 0.257 across the 10th to the 85th-90th percentile, or a 173 per cent increase, which is a solid indicator that digital transformation delivers considerably better value creation for well-performing banks. The fact that this incremental value is 0.163 throughout the distribution is a fabricated testament to the market leaders having a disproportionate share in integrating FinTech. Similarly, VAIC shows a monotonic increase from 0.031 at the lower quantiles to 0.083 at the higher quantiles, indicating that intellectual capital exhibits increasing marginal returns, most pronounced when banks are valued at a premium. The results show that innovation in FinTech is a highly significant contributor to Total Factor Productivity, and the quantile differences are statistically significant at the 10th percentile. This indicates that the effectiveness of FinTech use in improving bank performance is not evenly distributed, and the strongest performers have a greater capacity to convert technological expenditures into clear financial performance ( Hassan et al., 2025; Li et al., 2024).

The ESG disclosure also increases, ranging from 0.0021 to 0.0061, in the Q model of the Tobin, definitively suggesting that sustainability transparency is an effective enhancer of the valuation of strong market firms. This tendency is reflected in dividend policy, which increased its values from 0.052 to 0.169, indicating the greater power of payout signalling in promoting high-value entities. According to the research results, the noted positive correlation between FinTech and the performance of the bank is especially high in the higher quantiles, which proves that banks with more favourable initial performance metrics are in a position to use the FinTech investments to improve their financial results ( Hassan et al., 2025). This shows that FinTech leads to greater performance improvements in banks with lower performance, as they can access financial services previously unavailable ( Hassan et al., 2025).

The Market-to-Book version further widens this heterogeneity: FinTech impacts increase fourfold, to 0.364, highlighting the extreme sensitivity of equity markets to the adoption of digital technology in the elite enterprise. VAIC soars up by 0.046 up to 0.136, as the key position of knowledge efficiency is sealed. ESG coefficients almost tripled, justifying the predisposed investor reward for stability in well-valued companies. Even within the ROA model, where all coefficients are moderately scaled, the trend is persuasive. FinTech increases consistently by 0.0031 to 0.0118, VAIC increases similarly by 0.0009 to 0.0033, and dividends increase accordingly. This is a clear indication of how operational efficiencies from digitalisation, sustainability, and intellectual capital are focused within the ranks of the best banks. The results show an intent to conclude that, whereas FinTech can improve financial inclusion by expanding services to underserved populations, the advantages are more practically achieved by better-performing organisations, which can obtain economies of scale and strategically integrate technologies.

### 4.7 Deep neural network models

The deep neural network results in Table 10 address a different question from the econometric estimates. They assess whether the study variables improve out-of-sample prediction of firm value and whether nonlinear patterns are present. The DNN is compared with linear and tree-based benchmarks using a holdout period and firm-aware cross-validation. Improvements in test-set error indicate predictive information, not theoretical confirmation or causality. SHAP values are used to examine whether FinTech adoption, ESG disclosure, intellectual capital, and dividend policy contribute consistently to predictions and whether their marginal contributions vary across observations. When the SHAP ranking broadly accords with the panel estimates, the result strengthens confidence that the explanatory variables contain valuation-relevant information. Divergence between SHAP patterns and linear coefficients is interpreted as evidence of nonlinearity or interaction that warrants further research, rather than as a reason to replace the econometric findings.

**Table 10.** Deep Neural Network (DNN) model performance metrics.

Performance metric	(1) Tobin’s Q	(2) Market-to-Book	(3) ROA
**Training MSE**	0.041	0.072	0.000021
**Testing MSE**	0.049	0.083	0.000027
**Training MAE**	0.152	0.221	0.0036
**Testing MAE**	0.167	0.248	0.0042
**Training R** ^**2**^	0.742	0.768	0.701
**Testing R** ^**2**^	0.711	0.734	0.673
**Epochs (Optimal)**	120	130	110
**Hidden Layers**	3	3	3

The deep neural network results in Table 10 address a different question from the econometric estimates. They assess whether the study variables improve out-of-sample prediction of firm value and whether nonlinear patterns are present. The DNN is compared with linear and tree-based benchmarks using a holdout period and firm-aware cross-validation. Improvements in test-set error indicate predictive information, not theoretical confirmation or causality. SHAP values are used to examine whether FinTech adoption, ESG disclosure, intellectual capital, and dividend policy contribute consistently to predictions and whether their marginal contributions vary across observations. When the SHAP ranking broadly accords with the panel estimates, the result strengthens confidence that the explanatory variables contain valuation-relevant information. Divergence between SHAP patterns and linear coefficients is interpreted as evidence of nonlinearity or interaction that warrants further research, rather than as a reason to replace the econometric findings.

## V. Practical implication

The paper has significant practical implications for financial institutions, investors, and policymakers who must negotiate in the new environment of digital transformation and sustainability in emerging economies. Among managers of financial institutions, the recommendations are strongly in support of integrated strategic investments. Firstly, the adoption of FinTech is not only an operational improvement but also a powerful driver of firm value, proven to increase efficiency, customer experience, and risk management. The integration of FinTech should be a priority for managers, as it can be a critical element of competitive advantage and added value. Secondly, it is necessary to develop and report strong ESG practices. The analysis finds that increased ESG disclosure is associated with positive effects on firm value. It is also greatly enhanced by robust financial performance, as financially sound companies are better able to leverage ESG activities to convey a message of stability and attract sophisticated investors. It highlights the significance of not only implementing ESG but also making it transparent and reporting it, as well as aligning it with the financial well-being of the firm. Thirdly, it is essential to focus on constant investments in intellectual capital of human expertise, organisational processes, and customer relations. Such intangible assets are highly sensitive to market valuations, which are increasingly recognised as key to long-term competitiveness and innovation in the digital world. Lastly, it is essential to have an open, stable dividend policy. Long-term investors find dividends to be a strong indicator of financial stability and positive corporate governance, and their value-additional impact is even more evident when supported by explicit ESG reporting. Managers are thus supposed to design inclusive plans that balance technological change, human capital development, sustainability, and dividend payouts to maximise firm value.

To policymakers and regulators, the study provides sufficient reasons to advance frameworks that support digital transformation and sustainable finance. Policies which promote the uptake of FinTech by the financial industry would result in more robust, productive, and transparent markets. The regulators are supposed to fit into existing structures to support technological innovation and proactively address the risks involved. Moreover, the positive correlation between ESG disclosure and firm value was strong and significant, particularly in moderating financial performance, providing a solid rationale for strengthening ESG reportingand even making it. These mandates may reduce information asymmetry and investor confidence, and can be used to steer capital toward sustainable businesses. The observed heterogeneous effects and the disproportionate benefits enjoyed by higher-performing firms as a result of FinTech and ESG may indicate that a policymaker should seek to provide special assistance or incentives to get a broader and equitable involvement and impact on all financial institutions and avoid a worsening disparity between leaders and laggards in the digital and sustainability achievement.

Table 11 reports robustness checks designed to assess whether the main findings depend on model specification, variable construction, sample composition, or temporal ordering. The results confirm that the proposed mechanism remains stable across alternative tests. FinTech adoption continues to exert a positive and statistically significant effect on ESG disclosure in most specifications. The baseline coefficient of 0.214 remains close to the lagged-regressor estimate of 0.197, which indicates that the FinTech effect is not driven by simultaneity between current digital disclosure and current ESG reporting. The alternative FinTech measures also support the main findings. The TF-IDF-based FinTech index produces a coefficient of 0.188, while the binary FinTech indicator produces 0.176. Both remain statistically significant. This confirms that the result does not depend only on raw bag-of-words frequency. The finding strengthens the measurement validity of the FinTech index because different coding approaches generate consistent direction and significance.

**Table 11.** Robustness checks and sensitivity analysis.

Robustness Test	FIN → ESG	ESG → Dividend	Dividend → Tobin’s Q	Dividend → M/B	Dividend → ROA
Baseline FE model	0.214***	0.0068***	0.112**	0.186**	0.0065**
Lagged regressors	0.197***	0.0061**	0.104**	0.172**	0.0059**
TF-IDF FinTech index	0.188***	0.0063**	0.109**	0.178**	0.0061**
Binary FinTech indicator	0.176**	0.0057**	0.098*	0.164**	0.0054*
MVAIC instead of VAIC	0.203***	0.0065***	0.116**	0.191**	0.0067**
Intangible assets ratio	0.181**	0.0059**	0.101*	0.169**	0.0056*
Governance sub-score	0.226***	0.0072***	0.121**	0.199***	0.0069**
Social sub-score	0.198***	0.0064**	0.108**	0.181**	0.0062**
Environmental sub-score	0.142*	0.0041*	0.076	0.128*	0.0038
High-ESG firms	0.241***	0.0081***	0.137**	0.221***	0.0078**
Low-ESG firms	0.132*	0.0039	0.071	0.119*	0.0035
Large firms	0.237***	0.0078***	0.129**	0.214***	0.0074**
Small firms	0.151**	0.0046*	0.082*	0.137*	0.0042
Pre-2020 period	0.164**	0.0052**	0.094*	0.151**	0.0049*
Post-2020 period	0.248***	0.0079***	0.132**	0.218***	0.0075**
Placebo FinTech lead	0.031	0.0012	0.024	0.039	0.0011
System GMM check	0.186**	0.0058**	0.097*	0.152*	0.0051*

The intellectual capital robustness checks also support the study framework. When VAIC is replaced with MVAIC, the coefficient for FinTech adoption on ESG disclosure remains 0.203 and significant at the 1 percent level. When the intangible assets ratio is used as an alternative intellectual capital proxy, the coefficient remains positive at 0.181. This indicates that the results are not sensitive to one intellectual capital measure. It also supports the resource-based view because firms with stronger knowledge resources appear better able to convert digital capability into disclosure quality. The ESG sub-score results provide additional insight. Governance disclosure generates the strongest pathway, with FinTech adoption increasing governance disclosure by 0.226. The dividend and firm value effects are also strongest under the governance sub-score. This is consistent with the banking-sector context, where investors pay close attention to governance quality, risk control, board accountability, and disclosure discipline. Social disclosure also supports the mechanism, while environmental disclosure produces weaker effects. This weaker environmental channel is reasonable because banks have lower direct environmental exposure than manufacturing firms.

Subsample analysis shows that the mechanism is stronger among high-ESG and large firms. In high-ESG firms, the FinTech coefficient reaches 0.241 and the dividend effect on market-to-book rises to 0.221. This suggests that digital capability becomes more value-relevant when firms already possess stronger disclosure systems. Large firms also show stronger coefficients, which indicates that scale, resources, and investor visibility increase the valuation relevance of FinTech and ESG disclosure. The period-based analysis shows stronger post-2020 effects. The FinTech coefficient rises from 0.164 before 2020 to 0.248 after 2020. This pattern indicates that digital adoption became more central to disclosure and valuation after the acceleration of digital banking and remote financial services.

The placebo test is consistent with the proposed temporal ordering. Future FinTech adoption does not significantly predict current ESG disclosure or firm value in the reported specification. This result reduces one form of reverse-timing concern but does not establish causality, because unobserved time-varying factors and measurement error may remain.

## VI. Policy and managerial implications

The findings offer context-specific implications for regulators, bank managers, investors, and reporting bodies. Because the design uses observational data from one national market, the estimates are interpreted as robust associations rather than universal causal effects. The principal implication is that FinTech capability, intellectual capital, ESG reporting, and dividend policy should be governed as connected components of information quality and market communication. The results do not justify indiscriminate technology spending, mandatory payout targets, or the assumption that more disclosure always increases value. Benefits depend on data credibility, financial capacity, governance quality, and the institutional setting.

For regulators and policymakers, the evidence strongly supports integrated policy frameworks that treat FinTech adoption and ESG disclosure as interconnected domains. FinTech implementation is positively associated with firm value through enhanced transparency and efficiency in sustainability reporting, suggesting that regulators should accelerate digital transformation mandates while simultaneously strengthening ESG disclosure requirements. Policies modelled on the European Union Corporate Sustainability Reporting Directive could be adapted for emerging markets to reduce information asymmetry, restore investor confidence, and direct capital toward sustainable businesses. Additionally, given the critical role of intellectual capital (human, structural, and relational dimensions), regulatory tools should incentivise investments in intangible assets, such as through tax benefits or reporting credits for banks that demonstrate measurable progress in knowledge-based resources. To address heterogeneous effects—where higher-performing firms benefit disproportionately—policymakers should design targeted incentives or support programmes for smaller or lagging institutions to prevent widening gaps in digital and sustainability performance. Such measures would foster more resilient, transparent, and value-creating financial sectors overall.

For bank managers, the results emphasise the need for holistic, integrated strategies rather than siloed investments. FinTech should be positioned as a strategic enabler that links digital systems directly to ESG data management, real-time risk reporting, governance monitoring, and enhanced investor communication. Managers are encouraged to prioritise FinTech adoption not merely for operational efficiency but as a driver of competitive advantage and long-term firm valuation. Simultaneously, building and reporting strong ESG practices is essential, as these disclosures positively influence firm value and amplify the signalling effect of stable dividend policies. Financially sound banks are particularly well-placed to leverage this synergy, using robust performance to attract sophisticated investors through credible sustainability commitments. Equally important is sustained investment in intellectual capital—through talent development, organisational processes, and stakeholder relationships—which emerges as a key intangible driver of market valuation in the digital economy. Finally, managers should maintain consistent, transparent dividend policies, as these serve as credible signals of governance strength and are further enhanced when paired with explicit ESG reporting. An integrated approach balancing technological innovation, human capital development, sustainability transparency, and prudent shareholder returns maximises value creation.

For investors, the findings indicate that FinTech adoption, ESG disclosure, and dividend policy should be evaluated as a cohesive signal of firm quality. Digital capability becomes significantly more informative when accompanied by high-quality ESG transparency and stable payouts, as these elements collectively reduce uncertainty and enhance credibility. Investors are therefore advised to adopt multi-dimensional assessment frameworks that jointly consider a bank’s technological infrastructure, disclosure practices, intellectual capital strength, and dividend consistency. This approach is especially relevant in emerging markets, where information asymmetry is higher and where ESG reporting can moderate the positive effects of financial performance and dividend policy on valuation. By rewarding banks that demonstrate integrated digital-sustainability strategies, investors can better allocate capital toward institutions positioned for long-term resilience and superior returns.

For reporting bodies and standard setters, the study underscores the urgent need for a unified, consistent ESG measurement framework. Reliance on mixed or inconsistent data sources weakens comparability across banks and reduces the reliability of valuation signals. Reporting bodies should promote transparent, checklist-based disclosure indices or endorse a single, widely accepted provider to minimise measurement bias and enhance cross-institutional benchmarking. Standardisation would also facilitate the integration of FinTech-driven digital reporting systems, enabling higher frequency and quality of ESG data while supporting the broader policy goals of transparency and accountability.

## VII. Theoretical implication

The theoretical implication is a unified resource–disclosure–signal–value explanation. The resource-based view identifies FinTech capability and intellectual capital as the organisational resources that enable data collection, interpretation, control, and reporting. Stakeholder theory explains why firms deploy these resources to respond to demands for environmental, social, and governance accountability. Signalling theory and information-asymmetry arguments explain how credible ESG disclosure and sustainable dividend decisions influence investor assessment. Agency theory clarifies why dividend payments can limit managerial discretion over free cash flow. The theories are therefore complementary rather than parallel lists: resources enable disclosure, stakeholder demands give disclosure relevance, and disclosure together with payout policy creates signals that may be reflected in firm value. The findings support this integrated interpretation within the Bangladeshi financial sector, but they do not establish that the same mechanism has equal strength across other institutional environments.

## VIII. Conclusion

This study examined a sequential Digital–ESG–Dividend–Value mechanism among DSE-listed bank-based financial institutions during 2015–2023. The results indicate that FinTech adoption and intellectual capital are positively associated with ESG disclosure, that ESG disclosure is associated with dividend policy, and that dividend policy is associated with firm value. Financial performance strengthens the ESG–dividend association. Fixed-effects, endogeneity-adjusted, distributional, and predictive analyses provide broadly consistent evidence, although the observational design does not establish experimental causality. The theoretical contribution lies in integrating the resource-based view, stakeholder theory, signalling theory, information asymmetry, and agency theory into distinct stages of one mechanism. The practical implication is that digital systems and intangible resources create valuation relevance when they improve disclosure credibility, governance, and financially sustainable payout decisions. Regulators should prioritise comparable ESG standards, auditable digital reporting, and transparent disclosure of technology adoption. Managers should connect FinTech investment with staff capability, internal controls, and capital planning. These conclusions remain specific to the Bangladeshi listed financial sector and require validation in other institutional settings.

### 8.1 Limitations and references to further study

This study has several limitations. The sample contains 24 DSE-listed bank-based financial institutions and therefore offers limited statistical power and external generalisability. The nine-year period also constrains long-run estimation, so CS-ARDL results are treated as supplementary. The FinTech index measures disclosed adoption rather than audited technology expenditure or system usage. Although the keyword dictionary, validation checks, and alternative constructions improve reliability, annual-report language may contain strategic emphasis. The ESG index measures disclosure and may not fully represent underlying ESG performance. Moderate within-firm dispersion in ROA and FinTech adoption can reduce fixed-effects precision. Finally, fixed effects, lag structures, system GMM, and placebo tests reduce selected endogeneity concerns but do not eliminate all omitted-variable bias or establish causal effects. Future research should use regulatory shocks, digital-policy discontinuities, matched cross-country samples, audited technology expenditure, and external ESG assurance to strengthen identification and measurement.

Second, the FinTech adoption index is based on annual-report text. This approach captures disclosed digital activity, not necessarily actual technology intensity. Future studies can combine annual-report text with FinTech investment data, mobile banking transaction data, digital branch indicators, or IT expenditure.

Third, ESG disclosure is measured from public reporting. Disclosure may differ from actual ESG performance. Future studies can compare disclosure scores with third-party ESG ratings, regulatory penalties, board-level sustainability committees, or environmental and social performance indicators.

Fourth, the study uses observational panel data. Although lagged variables, fixed effects, and system GMM reduce endogeneity concerns, causal inference remains limited without an external shock. Future research can use regulatory reforms, FinTech adoption mandates, or ESG reporting rule changes as quasi-natural experiments.

Fifth, the study focuses on dividend policy as the main transmission channel from ESG disclosure to firm value. Future studies can examine other channels, such as risk reduction, cost of capital, analyst coverage, institutional ownership, and market liquidity.

## Ethics approval and consent to participate

Ethical approval and consent were not required.

## Declaration on the use of AI statement

The authors confirmed that no generative Artificial Intelligence (AI) tools were used in the conceptualization of this research or writing, data analysis, and interpretation of this study.

## Data Availability

Figshare. FinTech Adoption, ESG Disclosure, Intellectual Capital, Dividend Policy, and Firm Value: Panel Dataset of DSE-Listed Financial Institutions (2015–2023). https://doi.org/10.6084/m9.figshare.31418483. (Qamruzzaman, Md 2026) This work contains the following underlying data:
•
DSE_Banks_c_Dataset_2015_2023.csv Data are available under the terms of the Creative Commons Attribution 4.0 International license (CC-BY 4.0).
