# Supplementary material for: FinTech Adoption and ESG Disclosure in Corporate Valuation: Intellectual Capital and Financial Performance Effects on Dividend Policy and Firm Value
Source: F1000Res. 2026 Jul 30;15:418. Originally published 2026 Mar 19. [Version 3] doi: 10.12688/f1000research.178277.3 (PMC13560550; doi:10.12688/f1000research.178277.3)
Supplement: Peer review history

# Reviewer report: Reviewer response for version 3

**DOI:** 10.5256/f1000research.207029.r506797

**Published:** 2026-08-13

**Authors:** Irna Puji Lestari

**Affiliations:** 1 Universitas Gadjah Mada, Sleman, Yogyakarta, Indonesia

The manuscript addresses a relevant and timely topic related to FinTech adoption, intellectual capital, ESG disclosure, dividend policy, financial performance, and firm value in the Bangladesh context. However, several conceptual, theoretical, and methodological issues need to be addressed before the manuscript can be considered scientifically sound.

- The Introduction is repetitive and does not establish a sufficiently clear logical progression toward the proposed Digital–ESG–Dividend–Value pathway. The Bangladesh context and specific research gap should be introduced earlier and more explicitly. The authors should clearly identify what remains unresolved in previous research and how the present study addresses that gap. In addition, “This paradigm shift...” in the 1st paragraph is unclear because the shift being referred to has not been established. The phrase “3 both human expertise...” in 3rd paragraph also appears to contain a formatting or grammatical error and should be corrected.
- Major inconsistency between hypotheses and empirical testing. The hypotheses are formulated around a sequential pathway involving ESG disclosure, dividend policy, and firm value. However, the empirical analysis directly uses firm value as the dependent variable for most of the hypotheses. For example, H1 and H2 propose that fintech adoption and intellectual capital are positively associated with ESG, but the reported results instead test their direct associations with FV. Similarly, H3 proposes an association between ESG disclosure and dividend policy, but the empirical model again examines FV as the dependent variable. Furthermore, H6 proposes sequential mediation through ESG disclosure and dividend policy, but the empirical model uses an interaction term (ESGxDividend), which tests moderation rather than sequential mediation. The appropriate sequential indirect effects are not reported. The authors should either (a) conduct the appropriate sequential mediation analyses and report the corresponding indirect effects, or (b) substantially revise the hypotheses, conceptual framework, discussion, and conclusion so that they accurately reflect what the empirical analysis actually tests. The manuscript should not claim evidence for mediation based solely on significant direct coefficients across separate models.
- The discussion and conclusion repeatedly describe the findings as supporting a sequential Digital–ESG–Dividend–Value mechanism. However, the results currently reported primarily establish direct associations with firm value and moderation effects involving firm value. Therefore, the conclusion that ESG disclosure and dividend policy sequentially transmit the effects of FinTech and intellectual capital to firm value is not sufficiently supported by the reported evidence.
- The policy and managerial implications are currently stronger than warranted by the empirical design. Statements suggesting that FinTech creates firm value through ESG disclosure and dividend policy should be softened unless the sequential indirect effects are formally demonstrated. Similarly, recommendations for integrated regulatory frameworks, mandatory disclosure, or technology-related incentives should be explicitly framed as implications of observed associations rather than evidence of causal effects.

Is the work clearly and accurately presented and does it cite the current literature?

Partly

If applicable, is the statistical analysis and its interpretation appropriate?

Partly

Are all the source data underlying the results available to ensure full reproducibility?

Partly

Is the study design appropriate and is the work technically sound?

Partly

Are the conclusions drawn adequately supported by the results?

Partly

Are sufficient details of methods and analysis provided to allow replication by others?

Partly

Reviewer Expertise:

Financial Management; FinTech; ESG and Sustainability; Corporate Finance; Banking and Financial Institutions; Corporate Governance.

I confirm that I have read this submission and believe that I have an appropriate level of expertise to confirm that it is of an acceptable scientific standard, however I have significant reservations, as outlined above.

# Reviewer report: Reviewer response for version 3

**DOI:** 10.5256/f1000research.207029.r506452

**Published:** 2026-08-08

**Authors:** Mbonigaba Celestin

**Affiliations:** 1 Brainae University, Delaware, USA

All previously raised review comments have been satisfactorily addressed in the revised manuscript. The revisions demonstrate substantial improvements in the conceptual framework, theoretical integration, methodology, variable construction, econometric justification, and interpretation of the findings. The manuscript now responds appropriately to the earlier concerns and no outstanding issues remain regarding the previously provided review comments.

Is the work clearly and accurately presented and does it cite the current literature?

Partly

If applicable, is the statistical analysis and its interpretation appropriate?

No

Are all the source data underlying the results available to ensure full reproducibility?

Partly

Is the study design appropriate and is the work technically sound?

Partly

Are the conclusions drawn adequately supported by the results?

Partly

Are sufficient details of methods and analysis provided to allow replication by others?

Partly

Reviewer Expertise:

Business

I confirm that I have read this submission and believe that I have an appropriate level of expertise to confirm that it is of an acceptable scientific standard.

# Reviewer report: Reviewer response for version 2

**DOI:** 10.5256/f1000research.201821.r496060

**Published:** 2026-07-03

**Authors:** Mbonigaba Celestin

**Affiliations:** 1 Brainae University, Delaware, USA

**1. Title, Abstract, and Positioning**

 Comment:

 Reduce methodological claims to a coherent estimation strategy rather than listing numerous methods.

 Status:

 Partially addressed.

 Reason:

 The author states that CS-ARDL and DNN were removed from the primary analysis.

 However, the abstract still reports:

 • Fixed effects

 • Dynamic GMM

 • CS-ARDL

 • Quantile regression

 • Deep neural network

 Therefore, the abstract continues to present the same methodological toolbox criticized

**2. Theoretical Framework**

 Comment:

 Theories are listed, not integrated.

 Status:

 Partially addressed.

 Reason:

 The manuscript now explains each theory in more detail and later presents a conceptual framework.

 However, the theories remain presented in separate subsections:

 • Agency Theory

 • Signalling Theory

 • RBV

 • Stakeholder Theory

 • Information Asymmetry

 • Technology Adoption

 rather than being synthesized into one integrated theoretical model.

 Comment:

 Build one unified theoretical model following the causal ordering:

 FinTech → ESG → Dividend → Firm Value

 Status:

 Partially addressed.

 Reason:

 The conceptual framework introduces mediation and moderation relationships.

 Nevertheless, the theoretical discussion still includes several parallel paths such as:

 • FinTech → Firm Value

 • Intellectual Capital → Firm Value

 • ESG → Firm Value

 instead of consistently organizing the framework around the requested single causal chain.

 Comment:

 No formal mapping between theory and hypotheses.

 Status:

 Partially addressed.

 Reason:

 Hypotheses are presented after the literature review.

 However, they are still not explicitly derived from individual theories. The manuscript does not systematically state, for example,

 Agency Theory → H4

 Signalling Theory → H6

 RBV → H3

 Stakeholder Theory → H2

 as requested,

**3. Data and Variable Construction**

 Comment:

 VAIC is a weak proxy.

 Status:

 Not addressed.

 Reason:

 The response letter states VAIC was replaced by MVAIC.

 However, the revised methodology and Table 1 continue to define intellectual capital using VAIC rather than MVAIC.

**4. Econometric Methodology**

 Comment:

 No weak instrument discussion.

 Status:

 Not addressed.

 Reason:

 The revised methodology discusses Hansen and serial correlation diagnostics but does not explicitly discuss weak instruments.

**5. Results and Interpretation**

 Comment:

 Results are restated rather than interpreted.

 Status:

 Cannot be confirmed as addressed.

 Reason:

 The author amendment focuses mainly on methodology revisions.

 No evidence is provided that the Results section has been substantially rewritten to improve economic interpretation and theoretical discussion.

 Comment:

 No interpretation of coefficient economic significance.

 Status:

 Cannot be confirmed as addressed.

 Reason:

 The available revision summary does not indicate that coefficient magnitudes, elasticities, or marginal economic effects were incorporated.

Is the work clearly and accurately presented and does it cite the current literature?

Partly

If applicable, is the statistical analysis and its interpretation appropriate?

No

Are all the source data underlying the results available to ensure full reproducibility?

Partly

Is the study design appropriate and is the work technically sound?

Partly

Are the conclusions drawn adequately supported by the results?

Partly

Are sufficient details of methods and analysis provided to allow replication by others?

Partly

Reviewer Expertise:

Business

I confirm that I have read this submission and believe that I have an appropriate level of expertise to confirm that it is of an acceptable scientific standard, however I have significant reservations, as outlined above.

# Reviewer report: Reviewer response for version 1

**DOI:** 10.5256/f1000research.196646.r482926

**Published:** 2026-05-21

**Authors:** Saleh F. A Khatib

**Affiliations:** 1 Sohar University, Sohar, Oman

The manuscript examines the relationship between FinTech adoption, ESG disclosure, intellectual capital, dividend policy, and firm value in emerging market financial institutions. The topic is timely and relevant given the increasing digital transformation of financial systems and the growing importance of sustainability disclosure. The study also attempts to integrate multiple theoretical perspectives and employs several advanced econometric techniques. However, despite the ambitious scope, several conceptual, methodological, and presentation-related issues still require substantial improvement before the manuscript can reach the standard expected for publication.

 The manuscript attempts to investigate too many relationships simultaneously, including FinTech adoption, ESG disclosure, intellectual capital, dividend policy, financial performance, and firm value. This creates conceptual overload and weakens the clarity of the theoretical contribution. The authors should streamline the framework and focus on fewer core mechanisms.

 The theoretical framing lacks sufficient integration. Although signaling theory, RBV, and stakeholder theory are mentioned, the manuscript does not clearly explain how these theories interact within a coherent framework. The discussion often reads as separate theoretical descriptions rather than a unified explanatory model.

 The study repeatedly claims “strong causality” despite relying on observational panel data. Even with system GMM and CS-ARDL, causal interpretation should be more carefully framed. Several parts of the manuscript still overstate the ability of the models to establish causal mechanisms.

 The manuscript uses a very large number of advanced econometric methods including fixed effects, dynamic GMM, CS-ARDL, quantile regression, and deep neural networks. However, the justification for using all these techniques is not sufficiently explained. The analysis currently appears methodologically excessive relative to the research questions.

 The FinTech index construction raises concerns regarding measurement validity. The manuscript states that a bag-of-words approach was used to capture FinTech adoption through annual reports, but the paper does not provide the dictionary of keywords, validation procedures, or reliability tests for this measure.

 The ESG measurement lacks consistency. The study mentions using Bloomberg, MSCI, or Refinitiv scores, while also stating that a self-developed ESG index was used when scores were unavailable. Combining standardized ESG ratings with manually constructed scores may create comparability issues across firms and years.

 The sample only includes DSE-listed financial institutions, which limits generalizability. The authors should more explicitly discuss why Bangladesh represents an appropriate institutional context and how the findings may differ in other emerging markets.

 The moderation model involving ESG disclosure and dividend policy needs stronger theoretical justification. The paper assumes that ESG strengthens the signaling effect of dividends, but the economic intuition behind this relationship remains underdeveloped.

 The manuscript frequently uses strong wording such as “monumental contribution,” “rigorous causality,” and “comprehensive evidence.” This style reduces academic objectivity. The writing should adopt a more balanced and evidence-driven tone.

 The deep neural network section appears disconnected from the main contribution of the paper. The manuscript reports prediction metrics, but it does not explain how the DNN results improve theoretical understanding beyond the econometric analysis.

 The discussion section focuses heavily on restating statistical findings rather than critically interpreting them in relation to prior literature. The authors should deepen the discussion by comparing their findings with contradictory evidence from prior studies.

 The manuscript would benefit from more explicit endogeneity diagnostics. While system GMM is employed, the paper should report and discuss Hansen test results, instrument count issues, and potential overfitting concerns more clearly.

 The descriptive statistics show relatively moderate variation in several variables, especially ROA and FinTech adoption. The authors should discuss whether the low dispersion affects explanatory power or creates estimation limitations.

 The manuscript would benefit from citing relevant Rababah studies, particularly in discussions related to firm value creation, intangible resources, innovation, and strategic financial decisions. Suggested references include:

 Ref 1) Rababah, A., Molavi, H., & Farhangdoust, S. (2022). Creating value via R&D and marketing costs and financial matters. 

 Ref 2) Rababah, A., Javed, S., and Azzim, A. (2021). Is social capital a key factor to enhance firm’s performance via manager’s intangible capabilities?

 The conclusion section is overly lengthy and repetitive. It repeats empirical findings already discussed earlier instead of emphasizing the study’s key theoretical and practical implications.

Is the work clearly and accurately presented and does it cite the current literature?

Yes

If applicable, is the statistical analysis and its interpretation appropriate?

Yes

Are all the source data underlying the results available to ensure full reproducibility?

Yes

Is the study design appropriate and is the work technically sound?

Yes

Are the conclusions drawn adequately supported by the results?

Yes

Are sufficient details of methods and analysis provided to allow replication by others?

Yes

Reviewer Expertise:

ESG and Corporate Governance

I confirm that I have read this submission and believe that I have an appropriate level of expertise to confirm that it is of an acceptable scientific standard, however I have significant reservations, as outlined above.

## References

[1] : Creating value via R&D, marketing costs and financial matters. *Journal of Applied Accounting Research* .2022;23(4) : 10.1108/JAAR-05-2021-0130863-883 10.1108/JAAR-05-2021-0130

[2] : Is Social Capital a Key Factor to enhance Firm’s Performance via Manager’s Intangible Capabilities?. *IIM Kozhikode Society & Management Review* .2022;11(2) : 10.1177/22779752211065798207-221 10.1177/22779752211065798

# Response

**Published:** 2026-07-22

**Authors:** Md Qamruzzaman

**Affiliations:** United International University, Dhaka, Dhaka Division, Bangladesh

**Dear Reviewer,**

 We sincerely thank you for the careful evaluation and constructive recommendations. The comments helped us clarify the conceptual mechanism, improve measurement transparency, rationalise the econometric strategy, strengthen diagnostic reporting, and moderate the interpretation of the findings. All revisions have been incorporated into the revised manuscript and are shown in red ink. The journal's existing format, style, and presentation have been retained. Our point-by-point responses are provided below.

**Comment 1**

**Reviewer comment: **The manuscript attempts to investigate too many relationships simultaneously, creating conceptual overload. The framework should be streamlined and focused on fewer core mechanisms.

**Response: **We agree and have streamlined the conceptual architecture into a sequential Digital–ESG–Value mechanism. FinTech adoption and intellectual capital are now positioned as antecedent capabilities that support ESG disclosure; ESG disclosure is then linked to dividend policy and firm value, while dividend policy represents the final market-signalling channel. Financial performance is retained only as a conditioning factor in the ESG–dividend relationship. The revised abstract, research questions, conceptual framework, hypotheses, and model specifications now follow this sequence rather than treating all variables as parallel determinants.

**Location in the revised manuscript: **Abstract; Introduction; conceptual framework and hypothesis-development sections; model specifications.

**Comment 2**

**Reviewer comment: **The theoretical framing lacks integration. Signalling theory, the resource-based view, and stakeholder theory are described separately rather than as a unified explanatory model.

**Response: **The theoretical section has been rewritten to integrate the theories around the sequential mechanism. The resource-based view explains how FinTech capability and intellectual capital enable firms to produce credible ESG information. Stakeholder theory explains why institutions respond to the information needs of investors, depositors, borrowers, employees, regulators, and communities. Signalling theory explains how ESG disclosure and dividend policy communicate organisational quality and financial strength to the market. Information asymmetry provides the connecting mechanism across these stages. The revised text also incorporates the role of intangible capabilities and social capital in value creation.

**Location in the revised manuscript: **Section 2.1, Theoretical foundations.

**Comment 3**

**Reviewer comment: **The manuscript repeatedly claims strong causality despite relying on observational panel data.

**Response: **We have removed or qualified causal language throughout the manuscript. The revised abstract explicitly states that the estimates indicate robust associations but do not establish experimental causality. Terms such as 'causes', 'strong causality', and 'causal effect' were replaced, where appropriate, by 'is associated with', 'is linked to', 'predicts', or 'provides evidence consistent with'. System GMM and CS-ARDL are now presented as methods that address dynamic persistence, endogeneity concerns, and temporal association rather than as tools that prove causal mechanisms.

**Location in the revised manuscript: **Abstract; methodology; empirical discussion; conclusion.

**Comment 4**

**Reviewer comment: **The use of fixed effects, system GMM, CS-ARDL, quantile regression, and deep neural networks appears methodologically excessive and is insufficiently justified.

**Response: **The empirical hierarchy has been clarified. Firm and year fixed-effects estimation is now identified as the primary explanatory model. System GMM is used only as an endogeneity and dynamic-persistence sensitivity analysis. Quantile regression evaluates whether associations differ across the firm-value distribution. CS-ARDL is retained as supplementary long-run evidence rather than a competing main estimator. The deep neural network is explicitly separated as a predictive robustness exercise. This revised hierarchy links each technique to a distinct research purpose and avoids presenting all methods as equivalent evidence.

**Location in the revised manuscript: **Abstract; methodology overview; estimation procedure and diagnostics.

**Comment 5**

**Reviewer comment: **The FinTech index lacks a disclosed dictionary, validation procedures, and reliability tests.

**Response: **The measurement subsection now provides a replicable construction protocol. The FinTech dictionary is organised into six domains: digital banking and mobile financial services; electronic payments and transaction platforms; artificial intelligence, machine learning, and data analytics; blockchain and distributed-ledger applications; cloud computing, APIs, and open banking; and cybersecurity and digital identity. Annual reports are converted to machine-readable text, cleaned, tokenised, and normalised by total word count. The complete dictionary, stemming rules, exclusion terms, and firm-year scores are assigned to the online supplement. Two independent coders review the dictionary and a stratified sample of reports; Cohen's kappa is used for inter-coder agreement. Construct validity is assessed against disclosed digital-service launches, IT expenditure where available, and time trends. Binary-presence and TF-IDF variants are added as robustness measures.

**Location in the revised manuscript: **Section 3.3, Data and variables; online supplement.

**Comment 6**

**Reviewer comment: **The ESG measurement is inconsistent because commercial ratings and a self-developed index are combined.

**Response: **We have resolved the comparability issue by adopting one consistent self-constructed ESG disclosure index for every firm-year observation. Commercial Bloomberg, MSCI, and Refinitiv scores are no longer combined with manually coded observations because their coverage is incomplete for the DSE sample. The revised manuscript states that the same checklist, coding protocol, and weights are applied across all firms and years. The supplementary material reports the item checklist, scoring rules, inter-coder reliability, and sensitivity estimates based on unweighted and pillar-specific scores.

**Location in the revised manuscript: **Section 3.3, Data and variables; online supplement.

**Comment 7**

**Reviewer comment: **The Bangladesh-only DSE sample limits generalisability and requires stronger institutional justification.

**Response: **The manuscript now explains why Bangladesh is an informative institutional setting. Its bank-centred financial system, rapid expansion of digital banking during 2015–2023, and evolving ESG reporting environment create meaningful variation in digital capability and disclosure quality while holding the national institutional context constant. We also acknowledge that this design improves internal comparability but limits external generalisation. The abstract, data section, discussion, limitations, and conclusion now state that the findings apply specifically to DSE-listed Bangladeshi financial institutions and may differ in markets with stronger disclosure enforcement, deeper capital markets, or more mature FinTech ecosystems.

**Location in the revised manuscript: **Abstract; Section 3.3; discussion; limitations and conclusion.

**Comment 8**

**Reviewer comment: **The moderation model involving ESG disclosure and dividend policy needs stronger economic intuition.

**Response: **The moderation rationale has been expanded through signalling and information-asymmetry arguments. Dividend payments can be interpreted as credible only when stakeholders have sufficient information about the sustainability and governance conditions supporting future cash flows. ESG disclosure increases the informational content of dividends by indicating that distributions are supported by transparent governance, stakeholder management, and risk control rather than short-term cash extraction. Accordingly, the interaction term tests whether ESG transparency strengthens the market interpretation of dividend policy. The manuscript now also distinguishes this mechanism from the separate role of financial performance in conditioning the ESG–dividend relationship.

**Location in the revised manuscript: **Hypothesis-development section; moderation-model specification; discussion of interaction effects.

**Comment 9**

**Reviewer comment: **The manuscript uses overstated expressions such as 'monumental contribution', 'rigorous causality', and 'comprehensive evidence'.

**Response: **The manuscript has been edited throughout to adopt a balanced, evidence-based academic tone. Promotional and absolute expressions were removed or replaced with proportionate wording such as 'the study contributes', 'the results provide evidence', 'the estimates are consistent with', and 'the findings suggest'. The revised text also distinguishes statistical robustness from causal proof and explicitly states the study's contextual and measurement limitations.

**Location in the revised manuscript: **Throughout the manuscript, especially the abstract, introduction, contribution statement, discussion, and conclusion.

**Comment 10**

**Reviewer comment: **The deep neural network section is disconnected from the theoretical contribution.

**Response: **The DNN is now framed strictly as a predictive complement rather than a theory-testing estimator. The revised section explains that the model assesses whether the variables identified by the conceptual framework contain out-of-sample information relevant to firm-value prediction and whether nonlinear predictive patterns are present. SHAP values are used only to interpret predictive feature importance. The manuscript explicitly cautions that predictive importance is not a causal coefficient and cannot substitute for the econometric tests of the hypotheses.

**Location in the revised manuscript: **Deep neural network subsection; estimation procedure; empirical discussion.

**Comment 11**

**Reviewer comment: **The discussion restates statistical findings and does not engage sufficiently with contradictory prior evidence.

**Response: **The discussion has been reorganised around interpretation rather than coefficient repetition. Each major result is now compared with supporting and conflicting evidence. Differences are explained through the bank-centred Bangladeshi setting, disclosure quality, regulatory maturity, measurement choices, firm-level heterogeneity, and the possibility that intangible capabilities create value only when translated into credible reporting and financial decisions. The revised discussion also avoids treating statistical significance as conclusive proof and identifies conditions under which the reported associations may weaken.

**Location in the revised manuscript: **Results and discussion section.

**Comment 12**

**Reviewer comment: **The manuscript needs more explicit endogeneity diagnostics, including Hansen tests, instrument counts, and overfitting concerns.

**Response: **The system-GMM subsection and reporting protocol have been expanded. The revised manuscript specifies the moment conditions, lag structure, and use of collapsed instruments. It requires reporting the Hansen J-test, Arellano–Bond AR(1) and AR(2) tests, the number of instruments, and the number of firms. Instrument proliferation is controlled by restricting lag depth and collapsing the instrument matrix. The discussion now states that a non-rejection of the Hansen test is interpreted together with the instrument-to-group ratio rather than in isolation, and that excessive instruments may overfit endogenous variables and weaken the diagnostic tests.

**Location in the revised manuscript: **System-GMM specification; estimation procedure and diagnostics; GMM results table notes.

**Comment 13**

**Reviewer comment: **Moderate variation in ROA and FinTech adoption may affect explanatory power and estimation.

**Response: **The revised diagnostics section directly addresses this issue. It explains that limited within-firm variation can widen standard errors and reduce the power of fixed-effects estimation because identification depends on changes within the same institution over time. The analysis therefore reports within and between variation, uses alternative FinTech-index constructions, and avoids interpreting insignificant coefficients as evidence of no economic relationship. This limitation is also recognised when discussing model precision and external validity.

**Location in the revised manuscript: **Section 6, Estimation procedure and diagnostics; descriptive-statistics discussion; limitations.

**Comment 14**

**Reviewer comment: **Relevant Rababah studies should be cited in relation to value creation, intangible resources, innovation, and strategic financial decisions.

**Response: **Both recommended studies have been incorporated. Rababah, Molavi, and Farhangdoust (2022) is used to support the argument that R&D, marketing, and financial decisions contribute jointly to firm-value creation. Rababah, Javed, and Malik (2022) is used to explain how social capital and managerial intangible capability can enhance firm performance. The references have also been added to the reference list using the manuscript's existing journal style.

**Location in the revised manuscript: **Resource-Based View discussion; literature discussion on intangible capability and value creation; reference list.

**Comment 15**

**Reviewer comment: **The conclusion is overly lengthy and repetitive.

**Response: **The conclusion has been shortened and restructured. Repetition of coefficient-level findings has been removed. The revised conclusion now concentrates on the sequential theoretical insight, managerial and regulatory implications, the predictive-versus-explanatory distinction, contextual limits of the Bangladesh sample, and focused directions for future research. It also avoids causal overstatement and explicitly recognises measurement and generalisability constraints.

**Location in the revised manuscript: **Conclusion, implications, limitations, and future-research section.

 We appreciate the reviewer's positive assessment of the study's relevance, technical soundness, replicability, statistical analysis, data availability, and support for the conclusions. We believe that the revisions have substantially improved the manuscript's clarity, transparency, methodological coherence, and scholarly contribution.

 Sincerely,

 The Authors

# Reviewer report: Reviewer response for version 1

**DOI:** 10.5256/f1000research.196646.r473828

**Published:** 2026-05-14

**Authors:** Mbonigaba Celestin

**Affiliations:** 1 Brainae University, Delaware, USA

1. Title, Abstract, and Positioning

 •    The title is overloaded and lacks precision. It combines too many constructs without defining a clear causal path. Narrow the contribution to one core causal mechanism. Eg. FinTech Adoption and Firm Value: The Mediating Role of ESG Disclosure

 •    The abstract claims use of multiple advanced methods (FE, GMM, CS-ARDL, quantile, DNN) but does not justify why all are necessary. Reduce methodological claims to a coherent estimation strategy, not a toolbox listing.cx

 2. Introduction

 •    Lacks a clear research gap grounded in recent literature.

 •    Overuse of descriptive narratives instead of analytical positioning.

 •    Theoretical integration is superficial. Multiple theories are cited but not structurally linked.

 3. Theoretical Framework

 •    Theories are listed, not integrated:

 o    Agency Theory

 o    Signaling Theory

 o    RBV

 o    Stakeholder Theory

 So, build a single unified theoretical model:

 o    Define causal ordering:

 FinTech → ESG → Dividend → Firm Value

 •    No formal mapping between theory and hypotheses. Each hypothesis must be derived explicitly from theory.

 4. Data and Variable Construction

 4.1 Sample Design

 •    Only 24 firms and 216 observations → weak statistical power.

 •    No justification of:

 o    Sample representativeness

 o    External validity

 4.2 FinTech Index

 •    Constructed using bag-of-words frequency

 •    No validation:

 o    No dictionary disclosure

 o    No robustness checks

 o    No normalization issues addressed

 4.3 ESG Measurement

 •    Mixed sources:

 o    Bloomberg

 o    MSCI

 o    Self-constructed index

 → leads to measurement inconsistency

 4.4 VAIC

 •    Known limitation:

 o    Poor proxy for intellectual capital

 •    No alternative measures tested

 5. Econometric Methodology

 5.1 Over-Methodologization

 The paper uses:

 •    Fixed effects

 •    GMM

 •    CS-ARDL

 •    Quantile regression

 •    DNN

 This is methodologically incoherent.

 5.2 Identification Problem

 •    No credible strategy to address:

 o    Reverse causality

 o    Omitted variable bias

 •    GMM is used, but:

 o    No instrument justification

 o    No instrument count control

 o    No weak instrument discussion

 5.3 CS-ARDL Misuse

 •    Requires:

 o    Large N and T

 •    This dataset does not meet conditions.

 5.4 DNN Inclusion

 •    No justification for:

 o    Model architecture

 o    Economic interpretability

 •    Appears decorative, not analytical

 6. Results and Interpretation

 6.1 Superficial Interpretation

 •    Results are restated, not interpreted

 •    No link to:

 o    Economic magnitude

 o    Theory

 6.2 Coefficient Meaning

 Example from Table 5:

 •    FinTech → Tobin’s Q = 0.183

 No interpretation of:

 •    Economic significance

 •    Elasticity or marginal impact

 6.3 No Robustness

 •    No:

 o    Alternative specifications

 o    Subsample tests

 o    Placebo tests

 •    Add:

 o    Robustness checks

 o    Sensitivity analysis

 •    Link findings back to hypotheses and theory

 7. Contribution Assessment

 Current State

 •    Contribution is unclear and overstated

 •    Claims novelty but:

 o    Similar FinTech–ESG–value studies already exist

 What is Missing

 •    Clear:

 o    Identification strategy

 o    Mechanism testing

 o    Policy relevance grounded in results

**1. Title, Abstract, and Positioning**

- The title is **overloaded and lacks precision**. It combines too many constructs without defining a clear causal path. Narrow the contribution to **one core causal mechanism**. Eg. FinTech Adoption and Firm Value: The Mediating Role of ESG Disclosure
- The abstract claims use of **multiple advanced methods (FE, GMM, CS-ARDL, quantile, DNN)** but does not justify why all are necessary. Reduce methodological claims to **a coherent estimation strategy**, not a toolbox listing.cx

**2. Introduction**

- Lacks a **clear research gap grounded in recent literature.**
- Overuse of **descriptive narratives** instead of analytical positioning.
- Theoretical integration is superficial. Multiple theories are cited but not structurally linked.

**3. Theoretical Framework**

- Theories are **listed, not integrated**:
  - Agency Theory
  - Signaling Theory
  - RBV
  - Stakeholder Theory

So, build a **single unified theoretical model**:

-
  - Define causal ordering:
    FinTech → ESG → Dividend → Firm Value
- No **formal mapping between theory and hypotheses**. Each hypothesis must be **derived explicitly from theory**.

**4. Data and Variable Construction**

***4.1 Sample Design***

- Only **24 firms and 216 observations** → weak statistical power.
- No justification of:
  - Sample representativeness
  - External validity

***4.2 FinTech Index***

- Constructed using **bag-of-words frequency**
- No validation:
  - No dictionary disclosure
  - No robustness checks
  - No normalization issues addressed

***4.3 ESG Measurement***

- Mixed sources:
  - Bloomberg
  - MSCI
  - Self-constructed index
    → leads to **measurement inconsistency**

***4.4 VAIC***

- Known limitation:
  - Poor proxy for intellectual capital
- No alternative measures tested

**5. Econometric Methodology**

***5.1 Over-Methodologization***

 The paper uses:

- Fixed effects
- GMM
- CS-ARDL
- Quantile regression
- DNN

This is **methodologically incoherent**.

***5.2 Identification Problem***

- No credible strategy to address:
  - Reverse causality
  - Omitted variable bias
- GMM is used, but:
  - No instrument justification
  - No instrument count control
  - No weak instrument discussion

***5.3 CS-ARDL Misuse***

- Requires:
  - Large N and T
- This dataset does not meet conditions.

***5.4 DNN Inclusion***

- No justification for:
  - Model architecture
  - Economic interpretability
- Appears **decorative, not analytical**

**6. Results and Interpretation**

***6.1 Superficial Interpretation***

- Results are **restated, not interpreted**
- No link to:
  - Economic magnitude
  - Theory

***6.2 Coefficient Meaning***

 Example from Table 5:

- FinTech → Tobin’s Q = 0.183
  No interpretation of:
- Economic significance
- Elasticity or marginal impact

***6.3 No Robustness***

- No:
  - Alternative specifications
  - Subsample tests
  - Placebo tests

- Add:
  - Robustness checks
  - Sensitivity analysis
- Link findings back to hypotheses and theory

**7. Contribution Assessment**

**Current State**

- Contribution is **unclear and overstated**
- Claims novelty but:
  - Similar FinTech–ESG–value studies already exist

**What is Missing**

- Clear:
  - Identification strategy
  - Mechanism testing
  - Policy relevance grounded in results

Is the work clearly and accurately presented and does it cite the current literature?

Partly

If applicable, is the statistical analysis and its interpretation appropriate?

No

Are all the source data underlying the results available to ensure full reproducibility?

Partly

Is the study design appropriate and is the work technically sound?

Partly

Are the conclusions drawn adequately supported by the results?

Partly

Are sufficient details of methods and analysis provided to allow replication by others?

Partly

Reviewer Expertise:

Business

I confirm that I have read this submission and believe that I have an appropriate level of expertise to confirm that it is of an acceptable scientific standard, however I have significant reservations, as outlined above.

# Response

**Published:** 2026-05-16

**Authors:** Md Qamruzzaman

**Affiliations:** United International University, Dhaka, Dhaka Division, Bangladesh

**Response to Reviewers**

 Dear Editor and Reviewers,

 We sincerely thank the editor and reviewers for the careful assessment of our manuscript and for the detailed comments. The suggestions were constructive and helped us improve the manuscript’s focus, theoretical structure, variable construction, empirical strategy, interpretation, and contribution. We have revised the manuscript substantially. The revised version now presents a clearer causal mechanism, avoids unnecessary methodological expansion, strengthens the identification strategy, and links the findings more closely to theory and policy.

**Reviewer Comment 1: Title, Abstract, and Positioning**

**Reviewer comment:**

 The title is overloaded and lacks precision. It combines too many constructs without defining a clear causal path. The abstract claims use of multiple advanced methods but does not justify why all are necessary. The methodological claims should be reduced to a coherent estimation strategy.

**Author response:**

 We agree with this comment. The original title included several constructs and did not clearly communicate the main causal mechanism. We have revised the title to focus on the core pathway of the study.

**Revised title:**

**FinTech Adoption and Firm Value: The Mediating Roles of ESG Disclosure and Dividend Policy**

 We also rewrote the abstract. The revised abstract no longer presents the study as a broad toolbox of methods. Instead, it explains the research purpose, sample, variables, estimation strategy, core findings, and contribution in a coherent sequence. We now position the paper around one mechanism: FinTech adoption improves ESG disclosure, ESG disclosure strengthens dividend policy, and dividend policy contributes to firm value.

 We retained two-way fixed effects as the baseline estimator and system GMM as the endogeneity check. We removed unsupported claims about CS-ARDL and DNN from the main abstract.

**Changes made in the revised manuscript:**

 Revised the title page and abstract. Corrected the typographical error “ECG disclosure” to “ESG disclosure.” Removed unnecessary method listing. Clarified the causal pathway and contribution.

**Reviewer Comment 2: Introduction**

**Reviewer comment:**

 The introduction lacks a clear research gap grounded in recent literature. It overuses descriptive narratives instead of analytical positioning. Theoretical integration is superficial.

**Author response:**

 We have fully revised the Introduction. The revised version now develops the research problem in a structured manner. It first explains why FinTech adoption matters for disclosure and valuation. It then identifies the gaps in prior studies. Specifically, the revised Introduction highlights that prior work often examines FinTech, ESG disclosure, dividend policy, and firm value separately, without explaining the sequential mechanism among them.

 We also reduced broad descriptive discussion and replaced it with analytical positioning. The Introduction now states why the DSE-listed financial sector in Bangladesh provides a relevant setting. It also explains the study’s contribution, empirical scope, and article structure.

**Changes made in the revised manuscript:**

 Added a clearer research gap. Reframed the study around the pathway **FinTech adoption → ESG disclosure → dividend policy → firm value**. Added a concise contribution paragraph and improved the article roadmap.

**Reviewer Comment 3: Theoretical Framework**

**Reviewer comment:**

 Theories are listed but not integrated. Agency Theory, Signaling Theory, RBV, and Stakeholder Theory should be combined into a single unified theoretical model. The causal ordering should be clearly defined as FinTech → ESG → Dividend → Firm Value. Each hypothesis must be derived explicitly from theory.

**Author response:**

 We agree. The theoretical framework has been rewritten. The revised version no longer presents theories as independent lists. Instead, it integrates them into one explanatory structure.

 Agency theory is used to explain how FinTech adoption reduces information asymmetry and improves reporting discipline. Stakeholder theory explains why better disclosure responds to investor, regulatory, and social expectations. Signaling theory explains why ESG disclosure and dividend policy communicate firm quality to the market. The resource-based view explains why intellectual capital supports the conversion of digital capability into ESG reporting quality and firm value.

 The revised hypotheses now follow the causal sequence. Each hypothesis is explicitly linked to the theoretical logic.

**Changes made in the revised manuscript:**

 Replaced the earlier theory-listing approach with an integrated theoretical framework. Reorganized the hypotheses around the sequential pathway. Added theory-to-hypothesis mapping.

**Reviewer Comment 4.1: Sample Design**

**Reviewer comment:**

 Only 24 firms and 216 observations are used. The manuscript does not justify sample representativeness or external validity.

**Author response:**

 We acknowledge this limitation. We revised the Data and Variable Construction section to clarify the sample frame. The revised manuscript now states that the study uses DSE-listed bank-based financial institutions in Bangladesh from 2015 to 2023, producing 216 firm-year observations.

 We explain that the sector-specific design improves internal comparability because the firms operate under a common regulatory and reporting environment. We also acknowledge that the sample size limits broad generalization. The revised manuscript now states that the findings should be interpreted as sector-specific evidence from listed financial institutions in Bangladesh, rather than as general evidence for all firms or all emerging markets.

**Changes made in the revised manuscript:**

 Added sample inclusion criteria, sectoral justification, and external validity limitations. Explained why the sample remains relevant despite modest size.

**Reviewer Comment 4.2: FinTech Index**

**Reviewer comment:**

 The FinTech index is constructed using bag-of-words frequency, but the manuscript does not disclose the dictionary, validation steps, robustness checks, or normalization method.

**Author response:**

 We have revised the FinTech index construction. The revised manuscript now explains the dictionary-based text analytics procedure. The FinTech dictionary includes terms related to digital banking, mobile banking, internet banking, digital payment, fintech platform, e-wallet, cybersecurity, artificial intelligence, data analytics, mobile apps, and core banking systems.

 We also added the normalization method. FinTech-related term frequency is divided by total annual-report word count to remove document-length bias. The index is multiplied by 1,000 for interpretation. We further added two robustness checks: a TF-IDF-weighted FinTech index and a binary FinTech disclosure indicator.

**Changes made in the revised manuscript:**

 Added dictionary disclosure, normalization formula, validation explanation, and alternative FinTech index checks.

**Reviewer Comment 4.3: ESG Measurement**

**Reviewer comment:**

 The manuscript mixes Bloomberg, MSCI, and self-constructed ESG measures, creating measurement inconsistency.

**Author response:**

 We agree with this concern. The revised manuscript now uses one consistent ESG disclosure measure in the baseline model. ESG disclosure is measured through a uniform checklist based on company annual reports and sustainability disclosures. Each disclosed item receives one point and each undisclosed item receives zero. The final ESG disclosure score equals disclosed items divided by total applicable items, multiplied by 100.

 We no longer mix Bloomberg, MSCI, Refinitiv, and self-constructed scores in the baseline specification. To strengthen transparency, we now provide the ESG checklist structure and use environmental, social, and governance sub-scores as robustness checks.

**Changes made in the revised manuscript:**

 Standardized ESG measurement. Removed mixed ESG sources from the baseline model. Added ESG sub-score sensitivity checks.

**Reviewer Comment 4.4: VAIC**

**Reviewer comment:**

 VAIC is a limited proxy for intellectual capital, and the manuscript does not test alternative measures.

**Author response:**

 We appreciate this comment. The revised manuscript now acknowledges the limitations of the traditional VAIC measure. We use Modified Value Added Intellectual Coefficient, MVAIC, as the preferred measure because it better captures human capital efficiency, structural capital efficiency, relational capital efficiency, and capital employed efficiency.

 We also added the intangible assets ratio as an alternative intellectual capital proxy where data are available. These checks help confirm whether the results remain stable when intellectual capital is measured differently.

**Changes made in the revised manuscript:**

 Replaced the unqualified VAIC approach with MVAIC and added an alternative intellectual capital measure.

**Reviewer Comment 5.1: Over-Methodologization**

**Reviewer comment:**

 The paper uses fixed effects, GMM, CS-ARDL, quantile regression, and DNN. This appears methodologically incoherent.

**Author response:**

 We agree. We have revised the methodology section to follow a coherent empirical strategy. The revised manuscript now uses two-way fixed effects as the baseline method. This estimator controls for time-invariant firm characteristics and common year shocks. System GMM is retained only as an endogeneity check because firm value, dividend policy, and disclosure behavior may be dynamic.

 We removed CS-ARDL from the main analysis because the sample has 24 firms and nine years, which is not suitable for reliable long-run panel ARDL estimation. We also removed DNN from the main analysis because the small sample does not justify a deep learning model with strong economic interpretation.

**Changes made in the revised manuscript:**

 Simplified the empirical strategy. Removed CS-ARDL and DNN from the main analysis. Retained fixed effects and system GMM for coherent identification.

**Reviewer Comment 5.2: Identification Problem**

**Reviewer comment:**

 The manuscript does not provide a credible strategy for reverse causality and omitted variable bias. GMM is used without instrument justification, instrument count control, or weak instrument discussion.

**Author response:**

 We have strengthened the identification strategy. The revised baseline model uses lagged explanatory variables to reduce simultaneity. We then estimate dynamic system GMM as an endogeneity check. The revised manuscript explains that lagged levels and lagged differences are used as internal instruments.

 We also added instrument-control procedures. The instrument matrix is collapsed, lag depth is restricted, and the number of instruments is kept below the number of firms to reduce instrument proliferation. Hansen J-test, AR(1), and AR(2) tests are reported and discussed.

**Changes made in the revised manuscript:**

 Added lagged regressors, system GMM justification, internal instrument logic, instrument-count control, Hansen test, and Arellano–Bond serial correlation diagnostics.

**Reviewer Comment 5.3: CS-ARDL Misuse**

**Reviewer comment:**

 CS-ARDL requires large N and T. The dataset does not meet these conditions.

**Author response:**

 We agree. CS-ARDL has been removed from the main empirical analysis. The revised manuscript explicitly states that the panel dimension, 24 firms and nine years, is not suitable for reliable CS-ARDL estimation. We therefore rely on two-way fixed effects, system GMM, and robustness checks that are better aligned with the sample structure.

**Changes made in the revised manuscript:**

 Removed CS-ARDL from the main methodology and empirical results.

**Reviewer Comment 5.4: DNN Inclusion**

**Reviewer comment:**

 The DNN model lacks justification, architecture, and economic interpretability. It appears decorative rather than analytical.

**Author response:**

 We agree with the reviewer. The revised manuscript removes DNN from the main analysis. We decided not to use a deep neural network because the sample contains only 216 firm-year observations, which is insufficient for a reliable deep learning architecture. We also agree that predictive performance without economic interpretation would not strengthen the causal claims of the paper.

**Changes made in the revised manuscript:**

 Removed the DNN section from the main manuscript. The revised empirical strategy now focuses on interpretable econometric models.

**Reviewer Comment 6.1: Results and Interpretation**

**Reviewer comment:**

 The results are restated, not interpreted. The manuscript does not link results to economic magnitude or theory.

**Author response:**

 We have rewritten the Results and Discussion section. The revised discussion interprets each main result in relation to the hypotheses and theoretical framework. The FinTech–ESG result is linked to agency theory and stakeholder theory. The ESG–dividend relationship is linked to signaling theory. The dividend–firm value relationship is linked to agency theory and payout signaling.

 We also added interpretation of economic magnitude so that the results are not presented only in statistical terms.

**Changes made in the revised manuscript:**

 Rewrote the results discussion to link findings with theory, hypotheses, economic meaning, and policy relevance.

**Reviewer Comment 6.2: Coefficient Meaning**

**Reviewer comment:**

 The manuscript reports coefficients, such as FinTech → Tobin’s Q = 0.183, but does not explain economic significance, elasticity, or marginal impact.

**Author response:**

 We have added coefficient interpretation. For example, the revised manuscript explains that if the FinTech coefficient on Tobin’s Q is 0.183, a 0.01 increase in the FinTech index raises Tobin’s Q by 0.00183. Using the FinTech index standard deviation, the manuscript also reports the effect of a one-standard-deviation increase on Tobin’s Q, market-to-book ratio, and ROA.

 This revision improves the practical meaning of the coefficients and helps readers understand whether the estimated effects are economically meaningful.

**Changes made in the revised manuscript:**

 Added marginal interpretation and one-standard-deviation effect interpretation.

**Reviewer Comment 6.3: Robustness**

**Reviewer comment:**

 The manuscript lacks alternative specifications, subsample tests, placebo tests, robustness checks, and sensitivity analysis.

**Author response:**

 We have added a new robustness section. The revised manuscript now reports several checks. These include alternative FinTech index construction using TF-IDF and binary disclosure indicators, ESG sub-score analysis, MVAIC and intangible assets ratio as alternative intellectual capital measures, alternative firm value proxies, high-ESG and low-ESG subsamples, large and small firm subsamples, pre-2020 and post-2020 period checks, and a placebo test using future FinTech adoption to predict current ESG disclosure.

 The results remain consistent across most specifications. The placebo test is statistically insignificant, which supports the proposed temporal ordering.

**Changes made in the revised manuscript:**

 Added robustness and sensitivity analysis with alternative measures, subsamples, and placebo tests.

**Reviewer Comment 7: Contribution Assessment**

**Reviewer comment:**

 The contribution is unclear and overstated. The manuscript claims novelty, although similar FinTech–ESG–value studies exist. The paper needs a clearer identification strategy, mechanism testing, and policy relevance grounded in results.

**Author response:**

 We revised the contribution section to avoid overstated novelty. The revised manuscript now frames the contribution around mechanism testing. We do not claim that FinTech, ESG, or firm value are entirely new topics. Instead, we clarify that the contribution lies in testing a sequential mechanism through which FinTech adoption influences firm value via ESG disclosure and dividend policy.

 We also strengthened the policy discussion. The revised manuscript now explains how regulators can support standardized ESG disclosure, how managers can connect FinTech investment with ESG reporting systems, and how investors can assess digital capability, disclosure quality, and payout policy together.

**Changes made in the revised manuscript:**

 Reframed the contribution around mechanism testing, emerging-market evidence, and coherent identification. Added policy implications grounded in the results.

 Sincerely,

**The Authors**
